# Supplementary material for: Multiplexing cell‐cell communication
Source: Mol Syst Biol. 2020 Jul 16;16(7):e9618. doi: 10.15252/msb.20209618 (PMC7365139; doi:10.15252/msb.20209618)
Supplement: Supplementary file 1 — Appendix [file MSB-16-e9618-s001.pdf]

APPENDIX for:

# Multiplexing cell-cell communication

John T. Sexton and Jeffery J. Tabor

## Contents

**Appendix Text** **2**

Appendix Text S1. Relating transcriptional signals to sfGFP fluorescence. ....2
Appendix Text S2. NOT gate models. ....4
Appendix Text S3. NOR gate models. ....5
Appendix Text S4. MUX model. ....6
Appendix Text S5. DEMUX model. ....7
Appendix Text S6. SENSOR-MUX-AHL model. ....8
Appendix Text S7. AHL-DEMUX model. ....9
Appendix Text S8. Gene expression dynamics models. ....10
Appendix Text S9. AHL production model. ....14
Appendix Text S10. Coculture simulations. ....16
Appendix Text S11. CS scaling laws. ....20

**Appendix Figures** **21**

Appendix Figure S1. Design of MUX and DEMUX sub-circuits. ....21
Appendix Figure S2. Probing NOT gate inputs and outputs. ....23
Appendix Figure S3. Comparisons of NOT gate models. ....24
Appendix Figure S4. NOR gate models. ....25
Appendix Figure S5. Sensor transfer functions. ....26
Appendix Figure S6. Characterization of the AHL cell-cell communication system. ....27
Appendix Figure S7. Faults in preliminary SENSOR-MUX-AHL. ....28
Appendix Figure S8. Design and characterization of NOT6*. ....29
Appendix Figure S9. DAPG sensor is too weak to control SELECT in preliminary AHL-DEMUX. ....30
Appendix Figure S10. Stronger DAPG sensor 2 correctly controls SELECT in AHL-DEMUX variant. ....31
Appendix Figure S11. Strong induction of AHL sensor causes output faults in AHL-DEMUX variant. ....32
Appendix Figure S12. Design and characterization of a reduced-strength AHL sensor. ....34
Appendix Figure S13. Reduced-strength AHL sensor recovers robust activation of single AHL-DEMUX output in response to AHL. ....35
Appendix Figure S14. Measuring the AHL production rate of LuxI in SENSOR-MUX-AHL cells. ....37
Appendix Figure S15. Dynamical CS response to DAPG induction. ....38
Appendix Figure S16. CS scaling laws. ....39
Appendix Figure S17. Plasmid maps. ....40
Appendix Figure S18. Genetic device schematics. ....41

**Appendix Tables** **42**

Appendix Table S1. Gate transfer function model parameters. ....42
Appendix Table S2. Sensor transfer function model parameters. ....43
Appendix Table S3. DAPG sensor variants. ....44
Appendix Table S4. Sequences of parts used in this study. ....45
Appendix Table S5. Plasmids used in this study. ....54
Appendix Table S6. Bacterial strains used in this study. ....56

**Appendix References** **61**

# Appendix Text

## Appendix Text S1. Relating transcriptional signals to sfGFP fluorescence.

Throughout this study, we used probe plasmids to report transcriptional signals as sfGFP fluorescence. To understand this relationship, we considered the following sfGFP expression and fluorescence model:

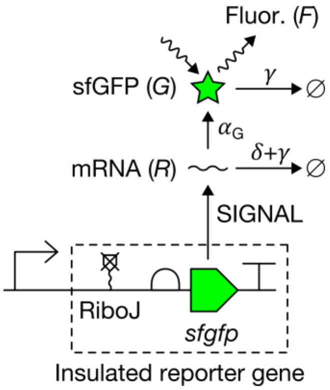


**Diagram of sfGFP expression and fluorescence model.**

Here, *R*, *G*, and *F* are sfGFP mRNA, protein, and fluorescence, respectively, SIGNAL is promoter transcription rate (mRNA time$^{-1}$), $\delta$ is mRNA degradation rate (time$^{-1}$), $\alpha_G$ is sfGFP translation rate (sfGFP mRNA$^{-1}$ time$^{-1}$), and $\gamma$ is dilution rate due to cell growth (time$^{-1}$). We assumed RiboJ renders $\alpha_G$ independent of the promoter sequence because it removes promoter-specific portions of the mRNA (Lou *et al*, 2012). We also assumed all sfGFP proteins mature instantaneously to their fluorescent state because we consistently employed a fluorophore maturation protocol (**Materials and Methods**). As a result, we expect sfGFP fluorescence to be proportional to number of sfGFP molecules:

$$F = \eta G \tag{1}$$

where $\eta$ is fluorescence per molecule (MEFL sfGFP$^{-1}$). To model expression dynamics, we used the following differential equations:

$$\frac{dR}{dt} = \mathrm{SIGNAL} - (\delta + \gamma)R \tag{2}$$

$$\frac{dG}{dt} = \alpha_G R - \gamma G \tag{3}$$

sfGFP fluorescence at steady state ($F_{\mathrm{SIGNAL}}^{SS}$) is therefore:

$$F_{\mathrm{SIGNAL}}^{SS} = \mathrm{SIGNAL}^{SS} \cdot \frac{\alpha_G}{\gamma^{SS}(\delta + \gamma^{SS})} \cdot \eta \tag{4}$$

$$= \mathrm{SIGNAL}^{SS} \cdot \left(k_{\mathrm{sfgfp}} \cdot \eta\right) \tag{5}$$

Here, $k_{\mathrm{sfgfp}}$ is a promoter-independent steady-state expression constant (sfGFP (mRNA time$^{-1}$)$^{-1}$). In the text, the ($k_{\mathrm{sfgfp}} \cdot \eta$) term is often omitted for simplicity and fluorescence signals are referred to by the transcriptional signals they report.

**Appendix Text S2. NOT gate models.**

We modeled our NOT gates by fitting the following Hill model to the measured NOT1-NOT9 and NOT6* transfer functions:

$$\mathrm{NOT}i(\mathrm{IN}_{\mathrm{NOT}i}) = \mathrm{OUT}_{\mathrm{NOT}i} = \mathrm{GATE}i_{\min} + \frac{(\mathrm{P}i_{\max} - \mathrm{GATE}i_{\min})}{1 + \left(\frac{\mathrm{IN}_{\mathrm{NOT}i}}{K}\right)^{n}} \tag{6}$$

Here, $\mathrm{P}i_{\max}$ is mean sfGFP fluorescence produced by P$i$ in the absence of S$i$ (MEFL), $\mathrm{GATE}i_{\min}$ is minimum gate output (MEFL), $K$ is the $\mathrm{IN}_{\mathrm{NOT}i}$ value at which $\mathrm{OUT}_{\mathrm{NOT}i}$ is half repressed (MEFL), and $n$ is the Hill coefficient (dimensionless), which describes the steepness of the transfer function. While $\mathrm{IN}_{\mathrm{NOT}i}$ and $\mathrm{OUT}_{\mathrm{NOT}i}$ are actually sfGFP fluorescence signals produced by their namesake transcriptional signals, it can be shown that $n$ is unaffected by the ($k_{\mathrm{sfgfp}} \cdot \eta$) sfGFP expression and fluorescence constant and the underlying $\mathrm{GATE}i_{\min}$, $\mathrm{P}i_{\max}$, and $K$ parameters can be calculated by dividing by ($k_{\mathrm{sfgfp}} \cdot \eta$). Thus, our transfer functions capture fundamental gate behavior and can be expected to accurately predict gate output given gate input.

To fit these models, we performed a constrained least-squares fit using the Lmfit Python package (Newville *et al*, 2014). $\mathrm{GATE}i_{\min}$, $K$, and $n$ were fit to pairs of $\mathrm{IN}_{\mathrm{NOT}i}$ and $\mathrm{OUT}_{\mathrm{NOT}i}$ sfGFP fluorescence values using the default Levenberg-Marquardt fitting algorithm. $\mathrm{P}i_{\max}$ was fixed as mean sfGFP fluorescence produced by P$i$ in the absence of the gate sgRNA. $\mathrm{GATE}i_{\min}$ was constrained to $[0,\infty)$, $K$ was constrained to $[1\mathrm{e}{-5},\infty)$, and $n$ was constrained to $[1\mathrm{e}{-3},\infty)$. Initial parameter values were $\mathrm{GATE}i_{\min}$=50 MEFL, $K$=350 MEFL, and $n$=3.0. As with the RMSE calculation, the residual for each fit was calculated in $\log_{10}$ MEFL space (i.e. residual = $\log_{10}(\mathrm{OUT}_{\mathrm{NOT}i}^{\mathrm{measured}}) - \log_{10}(\mathrm{OUT}_{\mathrm{NOT}i}^{\mathrm{predicted}})$). Plots are shown in **Fig. 2E** and **Appendix Fig. S8B**, and the resulting fit parameters are listed in **Appendix Table S1**. Python data structures representing these models are included in **Code EV1**. Two data points were discarded from the NOT2 fit because $\mathrm{IN}_{\mathrm{NOT2}}$ was negative.

**Appendix Text S3. NOR gate models.**

We modeled NOR1-NOR9 and NOR6* by extending each corresponding NOT gate model with a second transcriptional input term ($\mathrm{IN}_{\mathrm{NOR}i}2$):

$$\mathrm{NOR}i(\mathrm{IN}_{\mathrm{NOR}i}1, \mathrm{IN}_{\mathrm{NOR}i}2) = \mathrm{OUT}_{\mathrm{NOR}i} = \mathrm{GATE}i_{\min} + \frac{(\mathrm{P}i_{\max} - \mathrm{GATE}i_{\min})}{1 + \left(\frac{\mathrm{IN}_{\mathrm{NOR}i}1 + \mathrm{IN}_{\mathrm{NOR}i}2}{K}\right)^{n}} \quad (7)$$

Here, $\mathrm{IN}_{\mathrm{NOR}i}1$ and $\mathrm{IN}_{\mathrm{NOR}i}2$ are mean sfGFP fluorescence (MEFL) reporting two independent transcriptional inputs. Plots are shown in **Appendix Fig. S4** and fit parameters are listed in **Appendix Table S1**.

**Appendix Text S4. MUX model.**

We modeled the MUX output by composing models of its component gates:

$$\mathrm{OUT_{MUX}} = \mathrm{NOR3(NOR5(IN_1, SELECT), NOR6(NOT2(SELECT), IN_2))} \quad (8)$$

$IN_1$, $IN_2$, and SELECT were modeled as {0, $P1_{max}$}, {0, $P9_{max}$}, and {0, $P4_{max}$}, respectively. $Pi_{max}$ values are listed in **Appendix Table S1**. Mean sfGFP fluorescence was summed with mean autofluorescence (145 MEFL) to simulate mean cellular fluorescence, which is shown in **Fig. 3A**. Simulations are also listed in **Dataset EV1**.

**Appendix Text S5. DEMUX model.**

We modeled the DEMUX outputs by composing models of its component gates:

$$\mathrm{OUT_{DEMUX}1} = \mathrm{NOR7(NOT8(IN_{DEMUX}), SELECT)} \quad (9)$$

$$\mathrm{OUT_{DEMUX}2} = \mathrm{NOR2\big(NOT8(IN_{DEMUX}), NOT9(SELECT)\big)} \quad (10)$$

$IN_{DEMUX}$ and SELECT were modeled as {0, $P_{R,max}$} and {0, $P3_{max}$}, respectively. $P3_{max}$ is listed in **Appendix Table S1**, and $P_{R,max}$ was measured to be 5864 MEFL. Mean sfGFP fluorescence was summed with mean autofluorescence (145 MEFL) to simulate mean cellular fluorescence, which is shown in **Fig. 3B**. Simulations are also listed in **Dataset EV1**.

**Appendix Text S6. SENSOR-MUX-AHL model.**

We constructed the SENSOR-MUX-AHL model from a modified MUX model, NOT1, NOT9, and NOT4 gate models, and all-or-none aTc, IPTG, and DAPG sensor models. The SENSOR-MUX-AHL output is described by:

$$\mathrm{OUT}_{\text{SENSOR-MUX-AHL}} = \mathrm{OUT}_{\text{MUX*}}(\mathrm{IN}_1, \mathrm{IN}_2, \mathrm{SELECT}) \tag{11}$$

where

$$\mathrm{OUT}_{\text{MUX*}} = \mathrm{NOR3}(\mathrm{NOR5}(\mathrm{IN}_1, \mathrm{SELECT}), \mathrm{NOR6*}(\mathrm{NOT2}(\mathrm{SELECT}), \mathrm{IN}_2)) \tag{12}$$

and

$$\mathrm{IN}_1 = \mathrm{NOT1}(\mathrm{OUT}_{\text{aTc sensor}}) \tag{13}$$
$$\mathrm{IN}_2 = \mathrm{NOT9}(\mathrm{OUT}_{\text{IPTG sensor}}) \tag{14}$$
$$\mathrm{SELECT} = \mathrm{NOT4}(\mathrm{OUT}_{\text{DAPG sensor}}) \tag{15}$$

The MUX model was modified to replace NOR6 with NOR6*, as described in the text. $\mathrm{OUT}_{\text{aTc sensor}}$, $\mathrm{OUT}_{\text{IPTG sensor}}$, and $\mathrm{OUT}_{\text{DAPG sensor}}$ were modeled as $\{0, P_{\text{tet,induced}}\}$, $\{0, P_{\text{tac,induced}}\}$, and $\{0, P_{\text{PhlF,induced}}\}$, respectively. $P_{\text{tet,induced}}$, $P_{\text{tac,induced}}$, and $P_{\text{PhlF,induced}}$ are mean sfGFP fluorescence produced by the aTc, IPTG, and DAPG sensors upon induction with 20 ng/mL aTc, 0.3 mM IPTG, and 100 µM DAPG (**Appendix Fig. S5**) and are 1146 MEFL, 8176 MEFL, and 3098 MEFL, respectively. Mean sfGFP fluorescence was summed with mean autofluorescence (219 MEFL) to simulate mean cellular fluorescence, which is shown in **Fig. 4**. Autofluorescence was slightly increased during this time period due to cytometer variability. Simulations are also listed in **Dataset EV1**.

**Appendix Text S7. AHL-DEMUX model.**

We constructed the AHL-DEMUX model from DEMUX, NOT3, and all-or-none AHL and DAPG sensor 2 models. The AHL-DEMUX outputs are described by:

$$\mathrm{OUT_{AHL\text{-}DEMUX}1} = \mathrm{OUT_{DEMUX}1}(\mathrm{IN_{DEMUX}}, \mathrm{SELECT}) \quad (16)$$
$$\mathrm{OUT_{AHL\text{-}DEMUX}2} = \mathrm{OUT_{DEMUX}2}(\mathrm{IN_{DEMUX}}, \mathrm{SELECT}) \quad (17)$$

where

$$\mathrm{IN_{DEMUX}} = \mathrm{OUT_{AHL\ sensor}} \quad (18)$$
$$\mathrm{SELECT} = \mathrm{NOT3}(\mathrm{OUT_{DAPG\ sensor\ 2}}) \quad (19)$$

Thus,

$$\mathrm{OUT_{AHL\text{-}DEMUX}1} = \mathrm{NOR7}\big(\mathrm{NOT8}(\mathrm{OUT_{AHL\ sensor}}), \mathrm{NOT3}(\mathrm{OUT_{DAPG\ sensor\ 2}})\big) \quad (20)$$
$$\mathrm{OUT_{AHL\text{-}DEMUX}2} = \mathrm{NOR2}\Big(\mathrm{NOT8}(\mathrm{OUT_{AHL\ sensor}}), \mathrm{NOT9}\big(\mathrm{NOT3}(\mathrm{OUT_{DAPG\ sensor\ 2}})\big)\Big) \quad (21)$$

$\mathrm{OUT_{AHL\ sensor}}$ and $\mathrm{OUT_{DAPG\ sensor\ 2}}$ were modeled as $\{0, \mathrm{P_{lux*,induced}}\}$ and $\{0, \mathrm{P_{PhlF,induced\ 2}}\}$, respectively. $\mathrm{P_{lux*,induced}}$ is mean sfGFP fluorescence produced by $\mathrm{RECEIVER_{J23115*}}$ upon induction with 100 nM AHL and is 4269 MEFL (**Appendix Fig. S12B**), and $\mathrm{P_{PhlF,induced\ 2}}$ is mean sfGFP fluorescence produced by DAPG sensor 2 upon induction with 100 µM DAPG and is 4932 MEFL. Mean sfGFP fluorescence was summed with mean autofluorescence (219 MEFL) to simulate mean cellular fluorescence, which is shown in **Fig. 5**. Autofluorescence was slightly increased during this time period due to cytometer variability. Simulations are also listed in **Dataset EV1**.

**Appendix Text S8. Gene expression dynamics models.**

To model the gene expression dynamics of our circuits, we first modeled the dynamics of individual gates and then linked those models to describe circuits. Each gate model describes production of the gate dCas9:sgRNA complex (C$i$) from a transcriptional input ($\mathrm{IN}_{\mathrm{GATE}i}$), and how C$i$ in turn affects the gate's transcriptional output ($\mathrm{OUT}_{\mathrm{GATE}i}$):

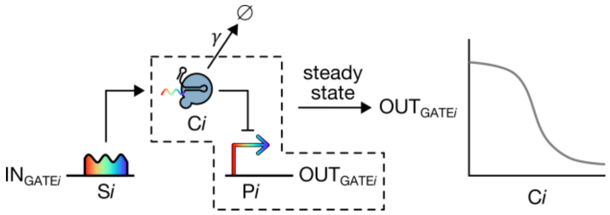


**Diagram of dynamical gate model.**

To start, we made some simplifying assumptions: (1) minimal sgRNA degradation occurs, due either to quick uptake and protection by dCas9 or low active mRNA degradation, (2) dCas9:sgRNA complex formation is fast relative to sgRNA synthesis, and (3) dCas9 is in excess. We also assumed C$i$ is stable and relies on dilution by cell growth for elimination. Based on these assumptions, we modeled the dynamics of C$i$ as follows:

$$\frac{d\mathrm{C}i}{dt} = \mathrm{IN}_{\mathrm{GATE}i} - \gamma \mathrm{C}i \tag{22}$$

where $\mathrm{IN}_{\mathrm{GATE}i}$ is total transcriptional input to the gate ($\mathrm{IN}_{\mathrm{NOT}i}$ for a NOT gate and $\mathrm{IN}_{\mathrm{NOR}i}1$+$\mathrm{IN}_{\mathrm{NOR}i}2$ for a NOR gate) (mRNA time$^{-1}$) and $\gamma$ is dilution rate of C$i$ due to cell growth (time$^{-1}$).

To relate $\mathrm{OUT}_{\mathrm{GATE}i}$ to C$i$, we invoked our steady-state transfer functions under the assumption that C$i$ and P$i$ rapidly equilibrate to steady state. First, we mapped C$i$ to $Fi^{TF}$, the sfGFP fluorescence that $\mathrm{IN}_{\mathrm{GATE}i}$ would concurrently generate from a probe plasmid under transfer function conditions:

$$Fi^{TF} = \gamma^{TF} \cdot \mathrm{C}i \cdot \left(k_{\mathrm{sfgfp}}^{TF} \cdot \eta\right) \tag{23}$$

Here, $\gamma^{TF}$ is cell growth rate and $\left(k_{\mathrm{sfgfp}}^{TF} \cdot \eta\right)$ is a steady-state sfGFP expression and fluorescence constant, each under transfer function conditions. Next, we used our transfer functions to calculate $F_{\mathrm{OUT}_{\mathrm{GATE}i}}^{TF}$, the sfGFP fluorescence that $\mathrm{OUT}_{\mathrm{GATE}i}$ would generate in response to $\mathrm{IN}_{\mathrm{GATE}i}$ under transfer function conditions:

$$F_{\mathrm{OUT}_{\mathrm{GATE}i}}^{TF} = \mathrm{NOT}i\left(Fi^{TF}\right) \tag{24}$$

Finally, we mapped $F_{\mathrm{OUT}_{\mathrm{GATE}i}}^{TF}$ to its underlying transcriptional signal:

$$\text{OUT}_{\text{GATE}i}^{TF} = \frac{1}{\left(k_{\text{sfgfp}}^{TF} \cdot \eta\right)} \cdot F_{\text{OUT}_{\text{GATE}i}}^{TF} \tag{25}$$

We then assumed, if C$i$ and P$i$ rapidly equilibrate, that gate output under transfer function conditions would match gate output in general as long as C$i$ was the same (i.e. $\text{OUT}_{\text{GATE}i}$ = $\text{OUT}_{\text{GATE}i}^{TF}$). Together, these relationships allow us to relate $\text{OUT}_{\text{GATE}i}$ to C$i$:

$$\text{OUT}_{\text{GATE}i} = \frac{1}{\left(k_{\text{sfgfp}}^{TF} \cdot \eta\right)} \cdot \text{NOT}i\left(\gamma^{TF} \cdot \text{C}i \cdot \left(k_{\text{sfgfp}}^{TF} \cdot \eta\right)\right) \tag{26}$$

In practice, we could not model our gates in this form because doing so would require estimating several unknown parameters (e.g. sfGFP translation rate, $\alpha_G$, and fluorescence per sfGFP, η). Instead, we used proxy sfGFP fluorescence signals derived from C$i$, $\text{IN}_{\text{GATE}i}$, and $\text{OUT}_{\text{GATE}i}$. $\text{IN}_{\text{GATE}i}$ and $\text{OUT}_{\text{GATE}i}$ were simply converted to the sfGFP fluorescence signals they would generate under transfer function conditions ($F_{\text{IN}_{\text{GATE}i}}^{TF}$ and $F_{\text{OUT}_{\text{GATE}i}}^{TF}$), and C$i$ was converted, as before, to $Fi^{TF}$. In this form, $F_{\text{IN}_{\text{GATE}i}}^{TF}$ represents the time-varying transcriptional input to the gate, $Fi^{TF}$ represents C$i$, and $F_{\text{OUT}_{\text{GATE}i}}^{TF}$ represents the transcriptional output from the gate, which can be calculated from $Fi^{TF}$ as NOT$i$($Fi^{TF}$).

To relate $Fi^{TF}$ to $F_{\text{IN}_{\text{GATE}i}}^{TF}$, we considered the dynamics of $Fi^{TF}$:

$$\frac{dFi^{TF}}{dt} = \frac{d}{dt}\left[\gamma^{TF} \cdot \text{C}i \cdot \left(k_{\text{sfgfp}}^{TF} \cdot \eta\right)\right] \tag{27}$$

$$= \gamma^{TF} \cdot \left(k_{\text{sfgfp}}^{TF} \cdot \eta\right) \cdot \frac{d\text{C}i}{dt} \tag{28}$$

Here, we incorporated the dynamical behavior of C$i$ and then expressed C$i$ and $\text{IN}_{\text{GATE}i}$ in terms of their proxy signals ($Fi^{TF}$ and $F_{\text{IN}_{\text{GATE}i}}^{TF}$, respectively):

$$\frac{dFi^{TF}}{dt} = \gamma^{TF} \cdot \left(k_{\text{sfgfp}}^{TF} \cdot \eta\right) \cdot \left[\text{IN}_{\text{GATE}i} - \gamma \text{C}i\right] \tag{29}$$

$$= \gamma^{TF} \cdot \left(k_{\text{sfgfp}}^{TF} \cdot \eta\right) \cdot \left[\left(\frac{F_{\text{IN}_{\text{GATE}i}}^{TF}}{\left(k_{\text{sfgfp}}^{TF} \cdot \eta\right)}\right) - \gamma \cdot \left(\frac{Fi^{TF}}{\gamma^{TF} \cdot \left(k_{\text{sfgfp}}^{TF} \cdot \eta\right)}\right)\right] \tag{30}$$

$$= \gamma^{TF} \cdot F_{\text{IN}_{\text{GATE}i}}^{TF} - \gamma \cdot Fi^{TF} \tag{31}$$

Rearranging this expression, we can interpret the dynamics of $Fi^{TF}$ as being driven to a growth-rate-corrected set point generated by $\text{IN}_{\text{GATE}i}$ with dynamics governed by γ, similar to previous models (Olson *et al*, 2014; Ramakrishnan & Tabor, 2016; Gander *et al*, 2017; Shin *et al*, 2020):

$$\frac{dFi^{TF}}{dt} = \gamma\left(\frac{\gamma^{TF}}{\gamma} \cdot F_{\text{IN}_{\text{GATE}i}}^{TF} - Fi^{TF}\right) \tag{32}$$

Together, $F_{\mathrm{IN}_{\mathrm{GATE}i}}^{TF}$, $Fi^{TF}$, and $F_{\mathrm{OUT}_{\mathrm{GATE}i}}^{TF}$ comprise a dynamical gate model. A time-varying $Fi^{TF}(t)$ signal can be calculated by numerically integrating $\frac{dFi^{TF}}{dt}$, and a gate output signal can be calculated as $F_{\mathrm{OUT}_{\mathrm{GATE}i}}^{TF}(t) = \mathrm{NOT}i\left(Fi^{TF}(t)\right)$.

To realize circuit models, we connected gate models together and to sensor outputs. First, we collected all $Fi^{TF}$ and $F_{\mathrm{OUT}_{\mathrm{GATE}i}}^{TF}$ terms into vectors:

$$\boldsymbol{c}(t) = [F1^{TF}(t), \cdots, F9^{TF}(t), F6^{*TF}(t)] \tag{33}$$

$$\boldsymbol{o}_{\mathbf{GATES}}(t) = \left[F_{\mathrm{OUT}_{\mathrm{GATE1}}}^{TF}\left(F1^{TF}(t)\right), \cdots, F_{\mathrm{OUT}_{\mathrm{GATE9}}}^{TF}\left(F9^{TF}(t)\right), F_{\mathrm{OUT}_{\mathrm{GATE6^*}}}^{TF}\left(F6^{*TF}(t)\right)\right] \tag{34}$$

To incorporate sensor output signals, we augmented $\boldsymbol{o}_{\mathbf{GATES}}$ with $F_{\mathrm{OUT}_{\mathrm{SENSOR}\ i}}^{TF}$, the sfGFP fluorescence that sensor *i* would generate from a probe plasmid under transfer function conditions:

$$\boldsymbol{o}(t) = \left[F_{\mathrm{OUT}_{\mathrm{aTc\ sensor}}}^{TF}(t), F_{\mathrm{OUT}_{\mathrm{IPTG\ sensor}}}^{TF}(t), F_{\mathrm{OUT}_{\mathrm{AHL\ sensor}}}^{TF}(t), F_{\mathrm{OUT}_{\mathrm{DAPG\ sensor}}}^{TF}(t) \mid \boldsymbol{o}_{\mathbf{GATES}}(t)\right] \tag{35}$$

We then linked gates and sensors together using a circuit connectivity matrix **M**, wherein $m_{ij}$ is 1 if transcriptional signal *i* expresses sgRNA S*j* and 0 otherwise. It follows that total transcriptional input to each gate can be calculated as the dot product of **o** and **M** (therein represented by sfGFP fluorescence proxy signals), which allows us to succinctly describe the dynamics of **c** as follows:

$$\frac{d\boldsymbol{c}}{dt} = \gamma^{TF} \cdot \boldsymbol{oM} - \gamma\boldsymbol{c} \tag{36}$$

Using this framework, different circuits can be described simply by changing **M**.

Lastly, we simulated sfGFP expression from every gate and sensor. Ideally, this would be done by decoding the transcription rates from $F_{\mathrm{OUT}_{\mathrm{GATE}i}}^{TF}$ and $F_{\mathrm{OUT}_{\mathrm{SENSOR}\ i}}^{TF}$ and using them to simulate detailed sfGFP expression models. However, this would again require estimating unknown parameters. Instead, we used a simple expression model like the one used for C*i*:

$$\frac{dG_i}{dt} = \mathrm{OUT}_i - \gamma G_i \tag{37}$$

Here, $\mathrm{OUT}_i$ is transcriptional output from gate or sensor *i*, $G_i$ is resulting sfGFP, and $\gamma$ is dilution rate due to cell growth. sfGFP fluorescence is therefore $F_{G_i} = G_i \cdot \eta$. While this model elides details like sfGFP translation and maturation, our consistent use of probe plasmids (which standardize the translation rate) and a fluorophore maturation protocol (**Materials and Methods**) minimize their impact. We then considered the dynamics of $F_{G_i}$:

$$\frac{dF_{G_i}}{dt} = \frac{d}{dt}[G_i \cdot \eta] \tag{38}$$

$$= \eta \cdot \frac{dG_i}{dt} \tag{39}$$

As with C$i$, we incorporated the dynamical behavior of $G_i$ and expressed $\mathrm{OUT}_i$ in terms of the proxy signals used in the circuit models:

$$\frac{dF_{G_i}}{dt} = \eta \cdot [\mathrm{OUT}_i - \gamma G_i] \tag{40}$$

$$= \eta \cdot \left[\frac{F_{\mathrm{OUT}_i}^{TF}}{\left(k_{\mathrm{sfgfp}}^{TF} \cdot \eta\right)} - \gamma \cdot \frac{F_{G_i}}{\eta}\right] \tag{41}$$

$$= \frac{1}{k_{\mathrm{sfgfp}}^{TF}} \cdot F_{\mathrm{OUT}_i}^{TF} - \gamma \cdot F_{G_i} \tag{42}$$

We then recognized that $k_{\mathrm{sfgfp}}^{TF} = \frac{1}{\gamma^{TF}}$ for our simple sfGFP expression model and arrived at the following expression for $F_{G_i}$ dynamics:

$$\frac{dF_{G_i}}{dt} = \gamma^{TF} \cdot F_{\mathrm{OUT}_i}^{TF} - \gamma \cdot F_{G_i} \tag{43}$$

As expected, this expression resembles the expression for the C$i$ proxy signal $Fi^{TF}$. As such, we can incorporate sfGFP expression into our circuit models simply by augmenting **c** and the columns of **M** with $F_{G_i}$ terms.

Our gene expression dynamics models therefore consist of a circuit connectivity matrix linking sensors, gates, and sfGFP expression (**M**), gate transfer functions (NOT$i$), cell growth rate ($\gamma$) (which may vary in time), cell growth rate under transfer function conditions ($\gamma^{TF}$), initial values for the C$i$ proxy and sfGFP fluorescence signals (**c**($t$=0)) (e.g. a steady-state solution), and externally driven transcriptional signals (e.g. sensor outputs). A model is then simulated by numerically integrated $\frac{d\mathbf{c}}{dt}$ (eqn. (36)) over a set of time points while simultaneously updating gate and sensor outputs (**o**). Python scripts performing these simulations are included in **Code EV1**.

**Appendix Text S9. AHL production model.**

To model AHL production in culture, we first modeled cellular LuxI expression and then total AHL accumulation. To model LuxI expression in the context of SENSOR-MUX-AHL, we used a simple expression model like those used for C*i* and sfGFP in the previous section:

$$\frac{dL}{dt} = \mathrm{OUT}_{\mathrm{NOR3}} - \gamma L \tag{44}$$

Here, LuxI ($L$) is produced from the output of NOR3, and cell growth (γ) governs its elimination. While LuxI bears an LVA *ssrA* degradation tag (Andersen *et al*, 1998) in our system, explicitly modeling its proteolysis would require significant additional characterization. Instead, we chose to use the model proposed above, wherein LuxI is synonymous with sfGFP expressed from NOR3 ($G_{\mathrm{NOR3}}$).

To model total AHL accumulation, we assumed each LuxI molecule synthesizes AHL at a constant rate *s* and that AHL degrades at rate $\kappa$:

$$\frac{dQ}{dt} = XLs - \kappa Q \tag{45}$$

Here, Q is AHL concentration (AHL volume$^{-1}$), $X$ is biomass concentration (cells volume$^{-1}$), $s$ is AHL synthesis rate per LuxI molecule (AHL volume$^{-1}$ time$^{-1}$ cell$^{-1}$ LuxI$^{-1}$), and $\kappa$ is AHL degradation rate (time$^{-1}$). At pH 6.6, where our experiments were performed, κ = 0.0115 hour$^{-1}$ (Tabor *et al*, 2009; Flagan *et al*, 2003; Schaefer *et al*, 2000). In practice, we could not directly measure *s* because we could not directly measure *L*. Instead, we considered $s'$, the AHL synthesis rate per LuxI expression (AHL volume$^{-1}$ time$^{-1}$ cell$^{-1}$ MEFL$^{-1}$), based on the assumption that concomitantly expressed sfGFP is a good proxy for LuxI:

$$\frac{dQ}{dt} = X \cdot F_{G_{\mathrm{NOR3}}} \cdot s' - \kappa Q \tag{46}$$

We then devised an experiment to measure $s'$, wherein SENSOR-MUX-AHL cells were grown expressing LuxI and their supernatant was periodically assayed for AHL (**Appendix Fig. S14**, **Materials and Methods**). To calculate $s'$, we first assumed an exponential growth biomass model ($X(t) = X_0 e^{\mu t}$) and then integrated $\frac{dQ}{dt}$ using an integrating factor:

$$Q(t) = e^{-\kappa t}\left[F_{G_{\mathrm{NOR3}}} \cdot s' \cdot X_0 \cdot \frac{1}{\kappa + \mu} \cdot \left[e^{(\kappa+\mu)t} - 1\right] + Q_0\right] \tag{47}$$

Here, $Q_0$ is initial AHL concentration (AHL volume$^{-1}$), $X_0$ is initial biomass concentration (cells volume$^{-1}$), $\mu$ is growth rate of SENSOR-MUX-AHL cells (time$^{-1}$), and $F_{G_{\mathrm{NOR3}}}$ is assumed to be constant. This expression can then be rearranged to expose the AHL production rate per cell ($p = F_{G_{\mathrm{NOR3}}} \cdot s'$) as the slope of a fit line:

$$Q(t) \cdot e^{\kappa t} = \left(F_{G_{\text{NOR3}}} \cdot s'\right) \cdot \left[X_0 \cdot \frac{1}{\kappa + \mu} \cdot \left[e^{(\kappa+\mu)t} - 1\right]\right] + Q_0 \quad (48)$$

Here, the left-hand side of the expression is a decay-corrected AHL term, and the right-hand side calculates decay-corrected biomass accumulation and scales it by $p$ before summing it with initial AHL. We fit $p$ and calculated $s'$ in **Appendix Fig. S14E**. To simulate AHL accumulation in culture, we then numerically integrated $\frac{dQ}{dt}$ (eqn. (46)) over a set of time points.

**Appendix Text S10. Coculture simulations.**

Bacterial cocultures were simulated for the CS linking (**Fig. 6**), multiplexing (**Fig. 7**), and DAPG response dynamics (**Appendix Fig. S15**) experiments per their descriptions in the **Materials and Methods**. All three experiments began with preconditioning monocultures, from which cocultures were made and then regularly diluted. For all cultures, cell growth, gene expression, and AHL accumulation were simulated. 1-min. time steps were used. Python scripts performing these simulations are included in **Code EV1**.

To simulate CS linking (**Fig. 6**), we first simulated the SENSOR-MUX-AHL and AHL-DEMUX preconditioning monocultures. Two sets of eight cultures were simulated, one set for each cell type induced with all eight combinations of aTc, IPTG, and DAPG. To start, sensor response to inducer was calculated. Sensor transcriptional outputs were assumed to respond instantaneously to inducer in all-or-none fashion and were modeled using appropriate values from the steady-state models (**Appendix Texts S6 and S7**). We then simulated gene expression (**Appendix Text S8**), wherein circuits were initialized to their no-inducer steady-state solutions and then simulated by numerically integrating equation (36) for all time points using a default cell growth rate of $\gamma = \gamma^{TF} = 0.85$ hour$^{-1}$. This growth rate was determined empirically over multiple experiments and used to calculate coculture dilution factors (**Materials and Methods**). Cell growth was then simulated assuming exponential growth with starting densities, growth durations, and final densities described in the **Materials and Methods**. Lastly, AHL accumulation was simulated in SENSOR-MUX-AHL cultures by numerically integrating equation (46) (**Appendix Text S9**). While minor growth rate inconsistencies existed between gene expression and cell growth, we chose to prioritize accurate final cell density because it significantly affects the resulting AHL concentration of the culture.

We then simulated cocultures derived from the preconditioned monocultures. Five successive sets of eight cocultures were simulated, with each set spanning 1.5 h. For each coculture, we began as before by calculating SENSOR-MUX-AHL sensor response. We then simulated SENSOR-MUX-AHL gene expression using the final state of the appropriate monoculture simulation as the initial state for the coculture simulation. A default growth rate of 0.85 hour$^{-1}$ was again used. Next, we simulated SENSOR-MUX-AHL cell growth. For the first set of cocultures, we assumed a SENSOR-MUX-AHL:AHL-DEMUX ratio of 10:1 and that cells grew exponentially for 1.5 h at a rate of 0.85 hour$^{-1}$ to a final total cell density of $OD_{600}$ = 0.3 (the **Materials and Methods** describe starting $OD_{600}$ values that factor in an additional ~15 min. to prepare the cocultures at the lab bench). For the remaining four sets of cocultures, starting SENSOR-MUX-AHL cell density was calculated by multiplying the cell density at the end of the previous simulation by the coculture dilution factor (0.28). AHL accumulation was then simulated as before by numerically integrating equation (46). For the first set of cocultures, initial AHL concentration was calculated by scaling the final AHL concentration of the appropriate SENSOR-MUX-AHL monoculture by the dilution factor needed to achieve the desired initial SENSOR-MUX-AHL cell density. For the remaining four sets of cocultures, starting AHL concentration was calculated from the previous simulation using the coculture dilution factor. AHL-DEMUX sensor response was then calculated. DAPG sensor 2 was simulated like the SENSOR-MUX-AHL sensors, and the AHL sensor output was simulated using the $RECEIVER_{J23115*}$ transfer function

and the accumulated AHL concentration. AHL-DEMUX gene expression was then simulated as described for SENSOR-MUX-AHL. Finally, AHL-DEMUX cell growth was simulated assuming exponential growth at a rate of 0.85 $hour^{-1}$. For the first set of cocultures, starting AHL-DEMUX cell density was again calculated based on a final total cell density of $OD_{600}$ = 0.3 and a SENSOR-MUX-AHL:AHL-DEMUX ratio of 10:1. For the remaining cocultures, initial cell density was calculated from the previous simulation using the coculture dilution factor. sfGFP fluorescence signals from the end of the simulation were then summed with mean autofluorescence (145 MEFL) and reported as model predictions in **Fig. 6** and **Dataset EV1**.

For multiplexing (**Fig. 7**), the first conversation was simulated like the CS linking simulation. Minor changes were made to the cell growth trajectories of the preconditioning monocultures, consistent with the **Materials and Methods**, and a 50:1 SENSOR-MUX-AHL:AHL-DEMUX ratio was used for the cocultures. Only the +IPTG +DAPG condition was simulated. To simulate conversation 2, we first applied a 1:100,000 dilution and switched inducers (+aTc −IPTG −DAPG) and then simulated one 13 h 45 min. coculture followed by five successive 1.5 h cocultures. Upon switching, we assumed IPTG and DAPG were completely eliminated and that aTc was fully induced. Initial cell densities and AHL concentrations were calculated from previous simulations using appropriate dilution factors, and cell growth rate was assumed to be 0.85 $hour^{-1}$ for growth and gene expression. sfGFP fluorescence signals from the ends of conversation 1 and conversation 2 simulations were again summed with mean autofluorescence (145 MEFL) and reported as model predictions in **Fig. 7** and **Dataset EV1**.

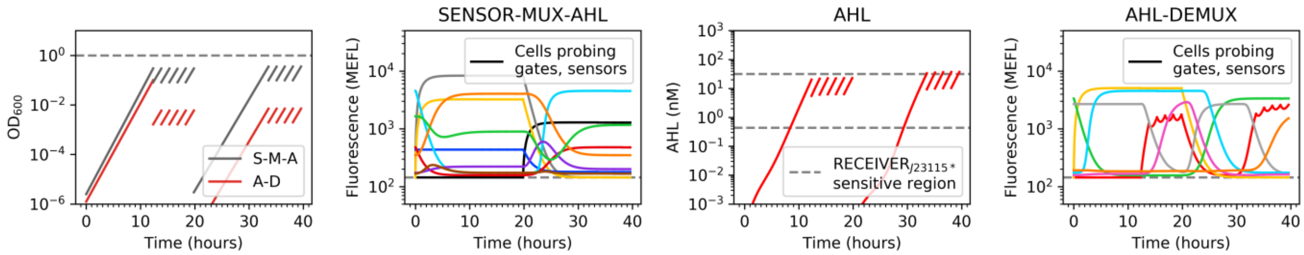


**Simulation of multiplexing experiment (Fig. 7).**

To simulate DAPG response dynamics (**Appendix Fig. S15**), we first developed a fixed-growth-rate simulation, like those developed for CS linking and multiplexing, and then we improved it by incorporating dynamical growth rate corrections calculated from experimental data. The fixed-growth-rate simulation assumed cell growth in coculture was exponential with a rate of 0.85 $hour^{-1}$. +IPTG preconditioning monocultures were simulated first, as described above and consistent with the **Materials and Methods**, followed by thirteen successive 1-hour cocultures. A 10:1 SENSOR-MUX-AHL:AHL-DEMUX ratio was assumed, a coculture dilution factor of 0.428 was used, and complete DAPG induction was assumed after the first coculture. While the resulting simulation reasonably predicted gene expression, it failed to capture changes in the observed SENSOR-MUX-AHL:AHL-DEMUX ratio, which increased from 10:1 to ~12:1 over the first ~5 hours of the experiment and then decreased to ~7.5:1 over next ~7 hours.

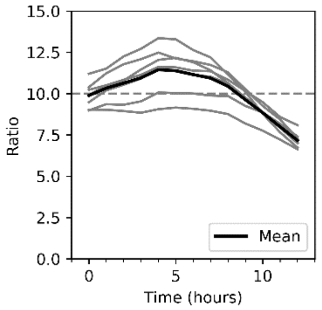


**Observed SENSOR-MUX-AHL:AHL-DEMUX ratio from the seven cocultures of the DAPG response dynamics experiment (Appendix Fig. S15).**

These changes are also evident in the numbers of SENSOR-MUX-AHL and AHL-DEMUX cells detected at each time point, which are comparable because cytometry sample acquisition time was standardized to 1 min. These changes likely reflect variations in cell growth rate; inflection points coincide with LuxI expression and AHL production, suggesting LuxI burdens SENSOR-MUX-AHL cells and possibly that AHL stimulates AHL-DEMUX cell growth. To account for this variability, we first calculated growth rate correction terms (ε) for both strains. Correction terms were calculated as the slope of the natural logarithm of the total cell count for the first 5 and last 3 hours of the SENSOR-MUX-AHL cell count data and for the last 5 hours of the AHL-DEMUX cell count data. Correction terms for each strain were then averaged across all 7 cocultures.

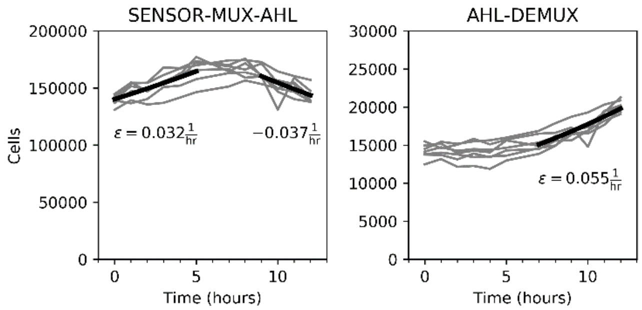


**Total cells detected and resulting growth rate corrections (ε).**
**(Time point t=6 h omitted due to minor transient cytometer blockage.)**

Next, we summed ε with 0.85 hour$^{-1}$, the growth rate that matches the coculture dilution rate, to calculate corrected growth rates. Dynamically corrected growth rates were then modeled using cubic splines to transition smoothly from 0.88 to 0.81 hour$^{-1}$ between t=5 h and t=9 h for SENSOR-MUX-AHL cells and from 0.85 to 0.90 hour$^{-1}$ between t=5 h and t=7 h for AHL-DEMUX cells.

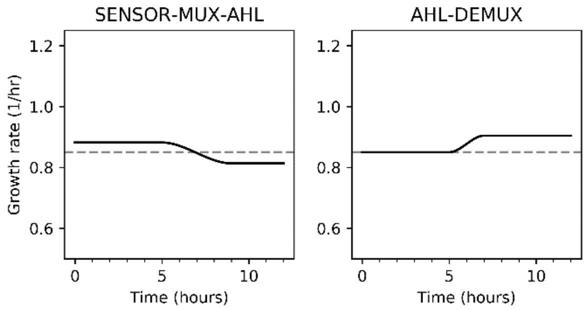


**Dynamically corrected growth rates.**

The simulation was then run again using the dynamically corrected growth rates. Exponential growth was still assumed, and biomass concentration was calculated by numerical integrating $\frac{dX}{dt} = \mu X$ where $X$ is biomass concentration and μ is growth rate. The simulated SENSOR-MUX-AHL:AHL-DEMUX ratio closely matched the observed ratio, and changes in simulated gene expression were minor.

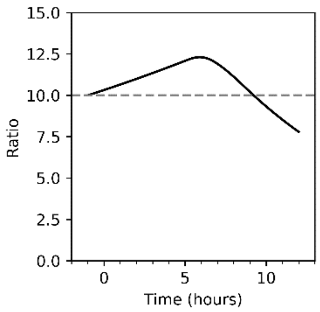


**Simulated SENSOR-MUX-AHL:AHL-DEMUX ratio.**

Simulated sfGFP fluorescence signals were then summed with mean autofluorescence (145 MEFL) and reported as model predictions in **Appendix Fig. S15** and **Dataset EV1**.

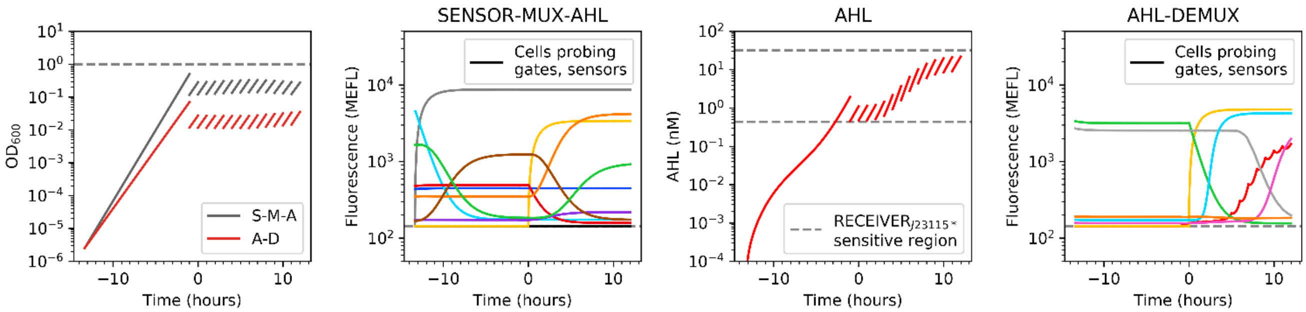


**Simulation of DAPG response dynamics experiment (Appendix Fig. S15).**

**Appendix Text S11. CS scaling laws.**

Mathematical descriptions of the CS scaling laws shown in **Appendix Fig. S16**. $c$ is number of channels and $s$ is number of bits required for the SELECT signal, which is $\lceil \log_2 c \rceil$.

For gates:

$$\text{gates}_{\text{MUX}}^{\text{2-layer}}(c) = c + s + 1 \tag{49}$$
$$\text{gates}_{\text{DEMUX}}^{\text{2-layer}}(c) = c + s + 1 \tag{50}$$
$$\text{gates}_{\text{CS}}^{\text{2-layer}}(c) = c + s + 1 \tag{51}$$
$$\text{gates}_{\text{MUX}}^{\text{Recursive}}(c) = 4(c - 1) \tag{52}$$
$$\text{gates}_{\text{DEMUX}}^{\text{Recursive}}(c) = 4(c - 1) \tag{53}$$
$$\text{gates}_{\text{CS}}^{\text{Recursive}}(c) = 4(c - 1) \tag{54}$$

For layers:

$$\text{layers}_{\text{MUX}}^{\text{2-layer}}(c) = 3 \tag{55}$$
$$\text{layers}_{\text{DEMUX}}^{\text{2-layer}}(c) = 2 \tag{56}$$
$$\text{layers}_{\text{CS}}^{\text{2-layer}}(c) = 6 \tag{57}$$
$$\text{layers}_{\text{MUX}}^{\text{Recursive}}(c) = 3s \tag{58}$$
$$\text{layers}_{\text{DEMUX}}^{\text{Recursive}}(c) = 2s \tag{59}$$
$$\text{layers}_{\text{CS}}^{\text{Recursive}}(c) = 5s + 1 \tag{60}$$

For maximum gate fan-in:

$$\text{max fan-in}_{\text{MUX}}^{\text{2-layer}}(c) = c \tag{61}$$
$$\text{max fan-in}_{\text{DEMUX}}^{\text{2-layer}}(c) = s + 1 \tag{62}$$
$$\text{max fan-in}_{\text{CS}}^{\text{2-layer}}(c) = c \tag{63}$$
$$\text{max fan-in}_{\text{MUX}}^{\text{Recursive}}(c) = 2 \tag{64}$$
$$\text{max fan-in}_{\text{DEMUX}}^{\text{Recursive}}(c) = 2 \tag{65}$$
$$\text{max fan-in}_{\text{CS}}^{\text{Recursive}}(c) = 2 \tag{66}$$

For maximum gate fan-out:

$$\text{max fan-out}_{\text{MUX}}^{\text{2-layer}}(c) = 2^{s-1} \tag{67}$$
$$\text{max fan-out}_{\text{DEMUX}}^{\text{2-layer}}(c) = c \tag{68}$$
$$\text{max fan-out}_{\text{CS}}^{\text{2-layer}}(c) = c \tag{69}$$
$$\text{max fan-out}_{\text{MUX}}^{\text{Recursive}}(c) = 1 \tag{70}$$
$$\text{max fan-out}_{\text{DEMUX}}^{\text{Recursive}}(c) = 2 \tag{71}$$
$$\text{max fan-out}_{\text{CS}}^{\text{Recursive}}(c) = 2 \tag{72}$$

## Appendix Figures

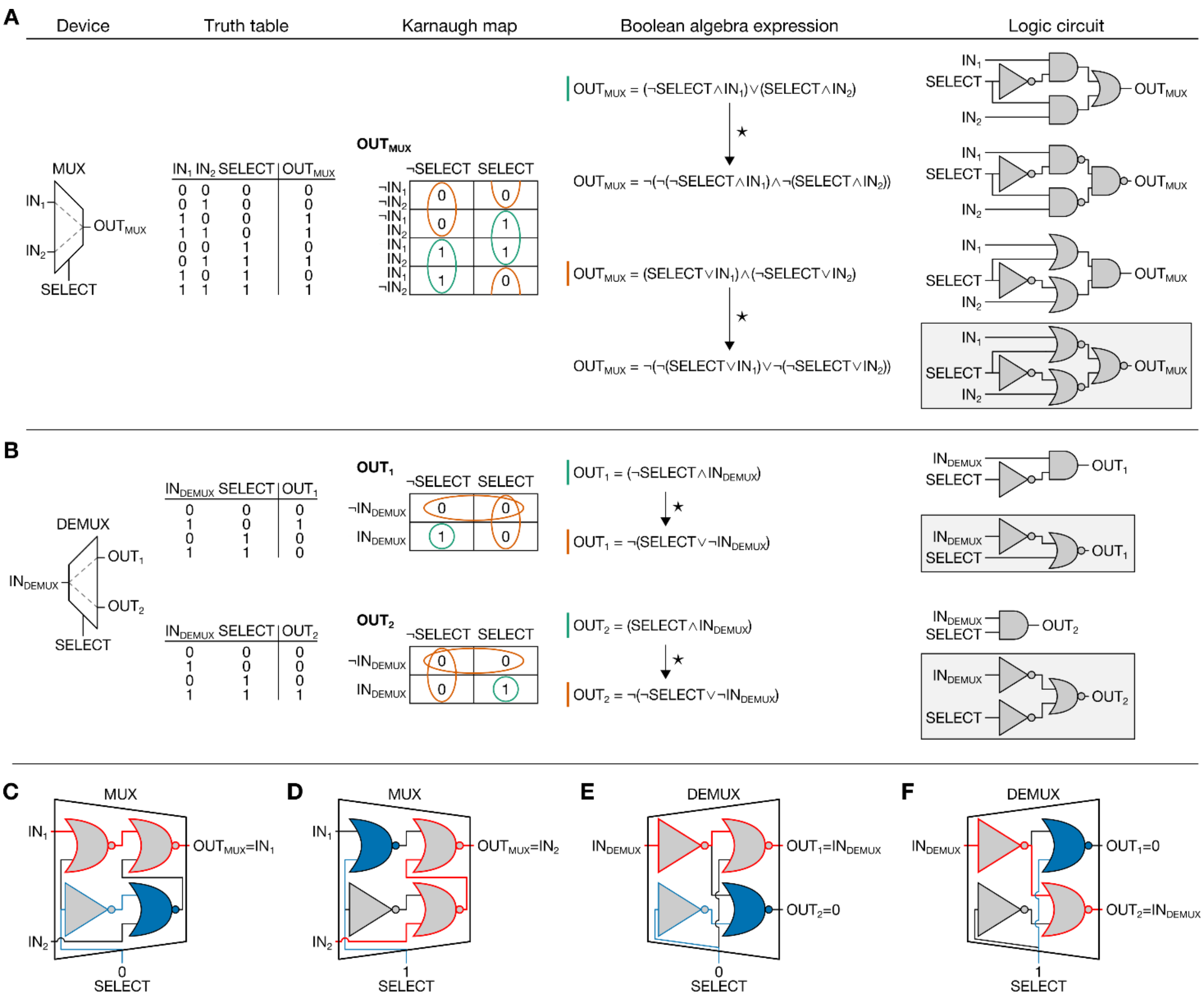


**Appendix Figure S1. Design of MUX and DEMUX sub-circuits.** Logic synthesis was used to design a (**A**) 2-input/1-output MUX and (**B**) 1-input/2-output DEMUX. First, the function of each circuit was described via a truth table, where all possible digital input combinations are listed alongside expected outputs. Karnaugh maps, which are graphical representations of truth tables organized to identify logic patterns, were then used to derive simplified Boolean algebra expressions for $OUT_{MUX}$, $OUT_1$, and $OUT_2$. Multiple synonymous minimized logic circuits were then created by reading each Boolean algebra expression. We selected circuits that only use NOR and NOT gates. The $OUT_1$ and $OUT_2$ circuits were combined to implement the DEMUX. ¬ specifies logical negation (NOT), ∧ specifies logical conjunction (AND), ∨ specifies logical disjunction (OR), and ⋆ specifies algebraic manipulation via De Morgan's law. (**C-F**) Gate-level illustrations of how the MUX and DEMUX select one input for propagation to the appropriate output. If either input to a NOR gate is a logical 1, the output will be a logical 0 independent of the other input. The MUX and DEMUX SELECT signals use this mechanism to halt the

propagation of $IN_2$ (**C**) or $IN_1$ (**D**) in the MUX and to deactivate $OUT_2$ (**E**) or $OUT_1$ (**F**) in the DEMUX. Solid blue gates have an input of 1 originating from SELECT (path highlighted in blue), and red highlights the path of the propagated input signal.

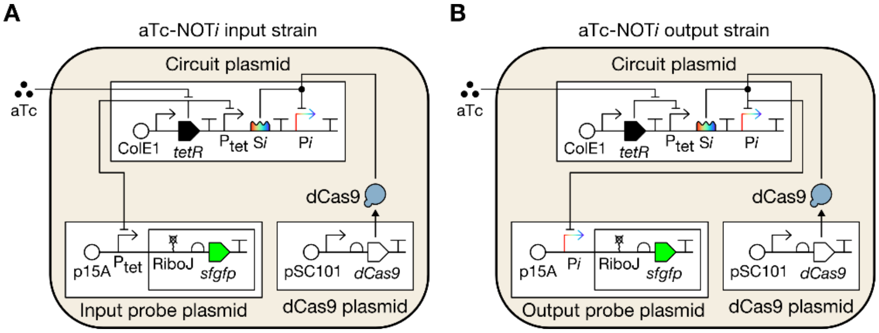


**Appendix Figure S2. Probing NOT gate inputs and outputs.** Schematics of aTc-NOT*i* (**A**) input and (**B**) output probe strains.

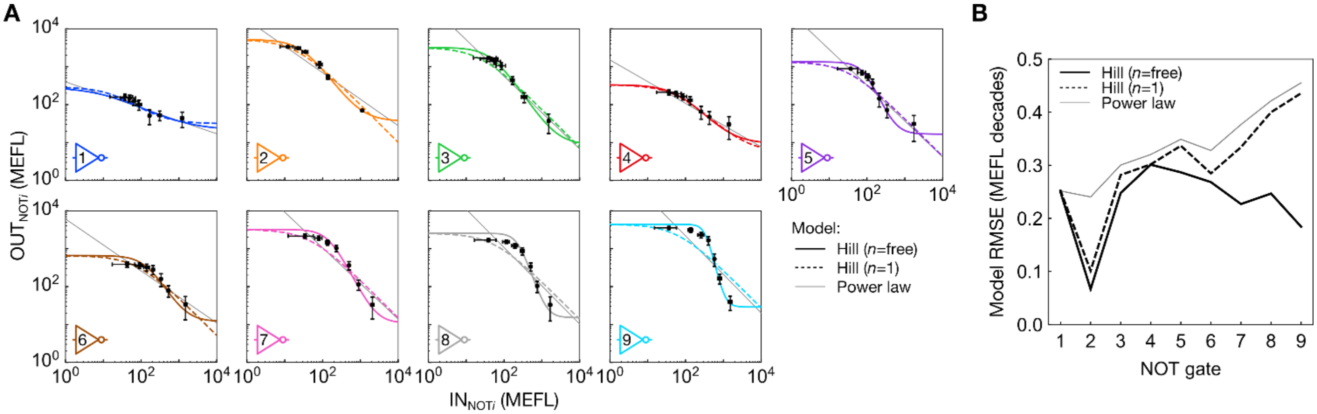


**Appendix Figure S3. Comparisons of NOT gate models.** (**A**) Unconstrained Hill (*n* = free), constrained Hill (*n* = 1), and power law models fit to each gate transfer function. Fit parameters for the unconstrained model are listed in **Appendix Table S1**. (**B**) Performance of each model for each gate. The constrained and unconstrained Hill models perform similarly for NOT1-6. The unconstrained model performs better for NOT7-9 as their transfer functions are more sigmoidal. The power law models perform similarly to the constrained Hill models.

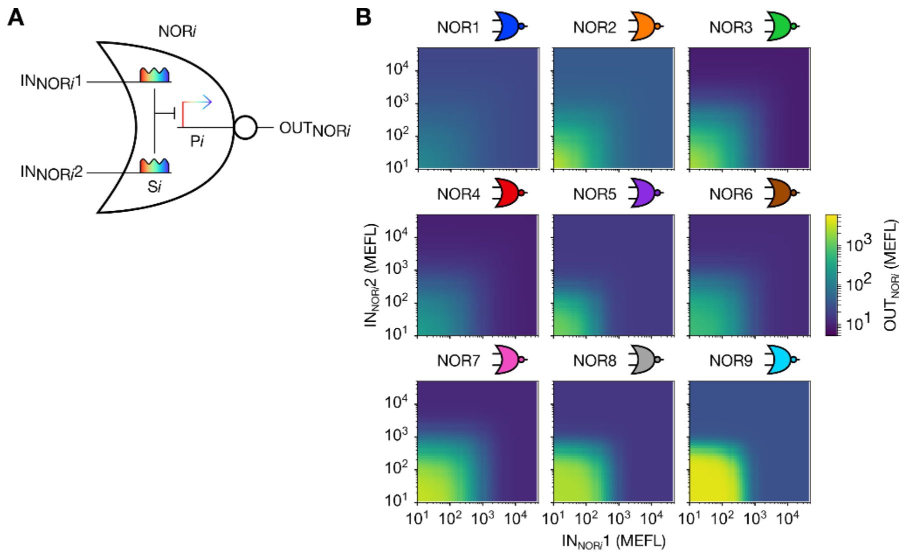


**Appendix Figure S4. NOR gate models.** (**A**) General schematic for a CRISPRi-based NOR gate. (**B**) Transfer function model simulations for NOR1-NOR9 (**Appendix Text S3**).

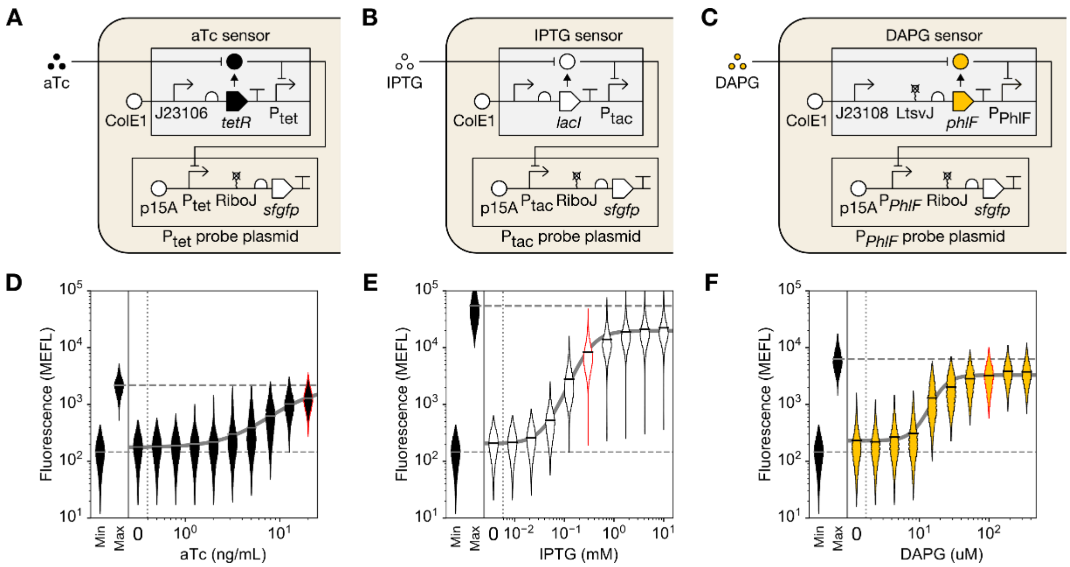


**Appendix Figure S5. Sensor transfer functions.** (**A-C**) Schematics of the strains used to measure sensor transfer functions. pSC31_3 is present in all strains but not pictured. (**D-F**) Sensor transfer functions. Violin plots (**Materials and Methods**) show mean and variation in sensor output over a range of input ligand concentrations. Min shows cellular autofluorescence and max shows sensor output when the repressor is absent (aTc and DAPG sensors) or expressed from the genome only (IPTG sensor). Solid gray lines show activating Hill model fits to mean sfGFP fluorescence (**Appendix Table S2**) summed with mean autofluorescence. The ligand concentrations chosen to activate each sensor in all main text experiments is highlighted in red (aTc = 20 ng/mL, IPTG = 0.3 mM, DAPG = 100 μM). All violins represent data combined from experiments on three separate days except for the max and DAPG sensor violins, where only one replicate was measured.

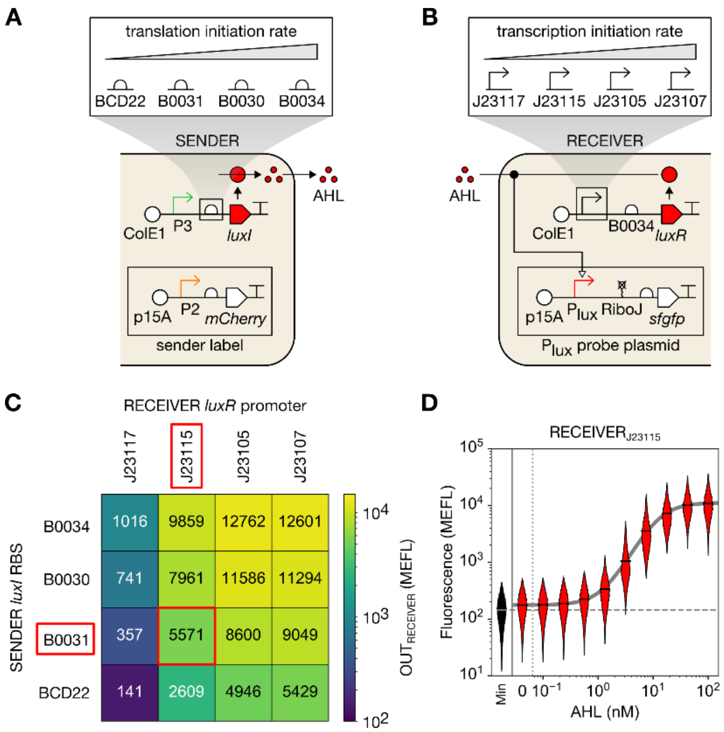


**Appendix Figure S6. Characterization of the AHL cell-cell communication system.** (**A**, **B**) Schematics of strains used to characterize the AHL cell-cell communication system. Four AHL SENDER strains were created expressing the AHL-biosynthetic enzyme LuxI from ribosome binding sites (RBSs) of varying strengths, and four AHL RECEIVER strains were created expressing the LuxR transcription factor from constitutive promoters of varying strengths. The MUX output promoter (P3) drives *luxI* to simulate its use in our CS, where it will transmit the output of the MUX to the DEMUX. SENDERs constitutively express mCherry so they can be identified by flow cytometry, and RECEIVERs express sfGFP from the AHL-responsive $P_{lux}$ promoter to report communication. pSC31_3 is present in all strains but not pictured. (**C**) Heatmap of mean sfGFP fluorescence produced by RECEIVERs after co-culturing them with all combinations of SENDERs for 5.75 h (**Materials and Methods**). Red box shows configuration chosen to connect the MUX to the DEMUX. In this configuration, $P_{lux}$ output closely matches $P_R$ output (5864 MEFL), which was previously used to generate $IN_{DEMUX}$ (**Fig. 3B**). (**D**) Transfer function of the selected RECEIVER strain. A violin plot shows cellular fluorescence as a function of exogenous AHL (**Materials and Methods**). All violins represent data combined from experiments on three separate days.

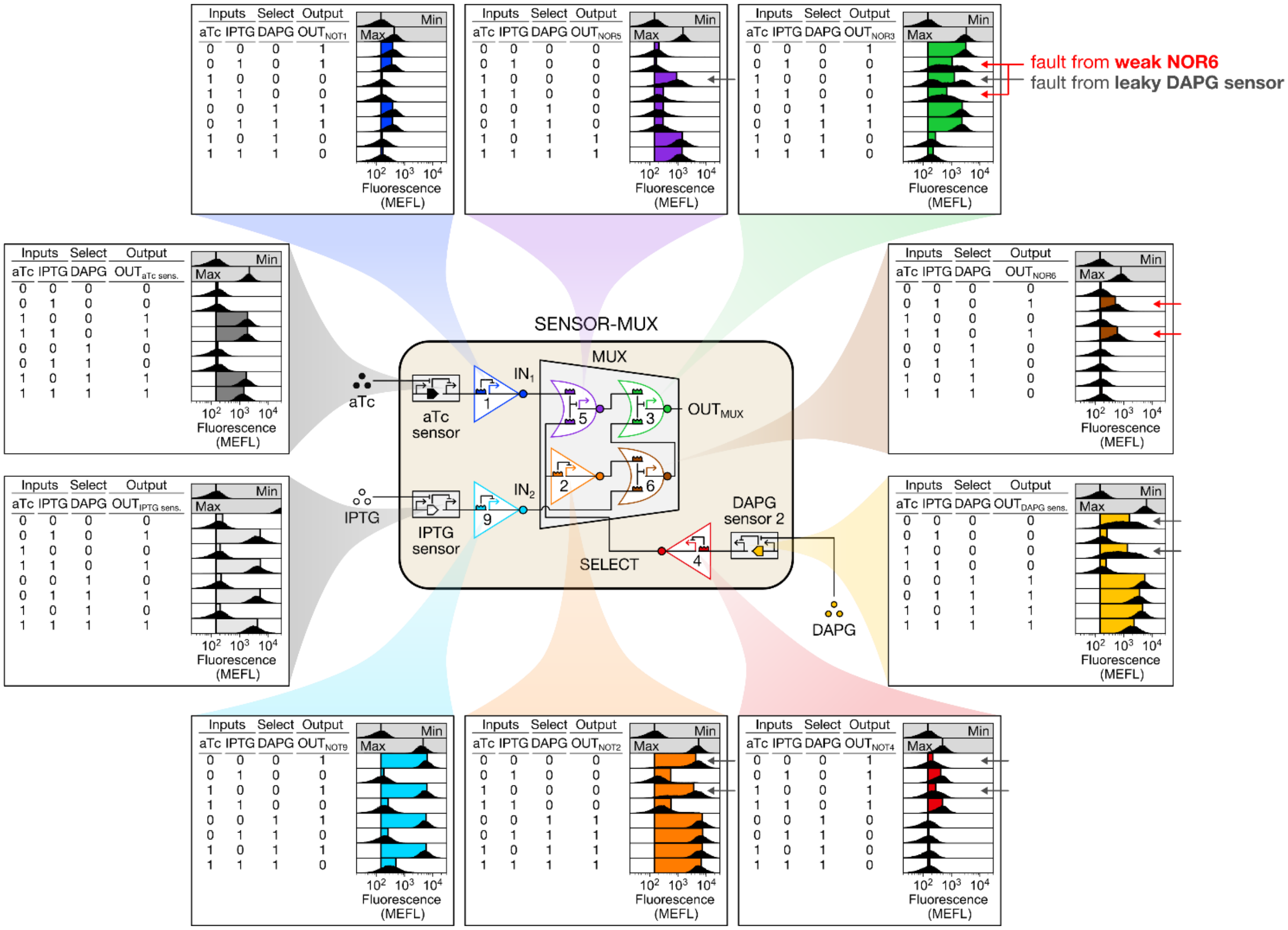


**Appendix Figure S7. Faults in preliminary SENSOR-MUX-AHL.** A preliminary SENSOR-MUX-AHL design ("SENSOR-MUX") was constructed by expressing S1, S9, and S4 from aTc, IPTG, and DAPG sensors in the context of the MUX. The SENSOR-MUX circuit plasmid was then separately co-transformed with the $P_{tet}$, $P_{tac}$, $P_{PhlF}$, P1, P2, P3, P4, P5, P6, and P9 probe plasmids, and the resulting ten strains were incubated with the eight possible binary combinations of aTc, IPTG, and DAPG (**Materials and Methods**). When DAPG was present (SELECT = 0), SENSOR-MUX recapitulated the MUX, but two faults arose when DAPG was absent (SELECT = 1). First, when IPTG was present ($IN_2$ = 0) $OUT_{MUX}$ reached only intermediate, rather than low, levels (red arrows), indicating that NOR6 was too weak to fully repress NOR3. Second, leaky transcription from DAPG sensor 2 caused only ~50% of cells to propagate $IN_2$ to $OUT_{MUX}$ when aTc was present ($IN_1$ = 0) and IPTG was absent ($IN_2$ = 1) (gray arrows). This second fault was corrected by replacing DAPG sensor 2 with a non-leaky DAPG sensor (**Appendix Table S3**, **Appendix Fig. S5**, **Fig. 4**). Min was measured in triplicate on three separate days, max was measured once on a fourth day, and all other measurements were performed on a fifth day. For sensors, max shows sensor output when the repressor is absent (aTc and DAPG sensors) or expressed from the genome only (IPTG sensor). Inducer concentrations: 0 (0); 20 ng/mL aTc, 0.3 mM IPTG, and 100 µM DAPG (1).

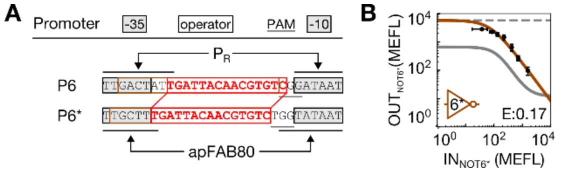


**Appendix Figure S8. Design and characterization of NOT6*.** (**A**) The NOT6 output promoter (P6) was originally designed by adding a PAM site to a randomly generated pre-sequence (red) and then flanking that sequence with -35 and -10 sequences from $P_R$ (**Materials and Methods**). To design P6*, we moved the P6 pre-sequence into the strong promoter apFAB80 (Mutalik *et al*, 2013) and insulated it with the original P6 insulator sequence (not shown). The NOT6* gate was then created by designing a new cognate sgRNA (S6*). (**B**) NOT6* transfer function. NOT6* was characterized exactly as NOT1-9 were; data are presented as in **Fig. 2E**. The solid gray line shows the original NOT6 transfer function for reference. NOT6* achieved almost 10-fold higher outputs relative to NOT6 but could still be repressed to low outputs (< 100 MEFL), suggesting it would appropriately repress NOR3 in SENSOR-MUX-AHL.

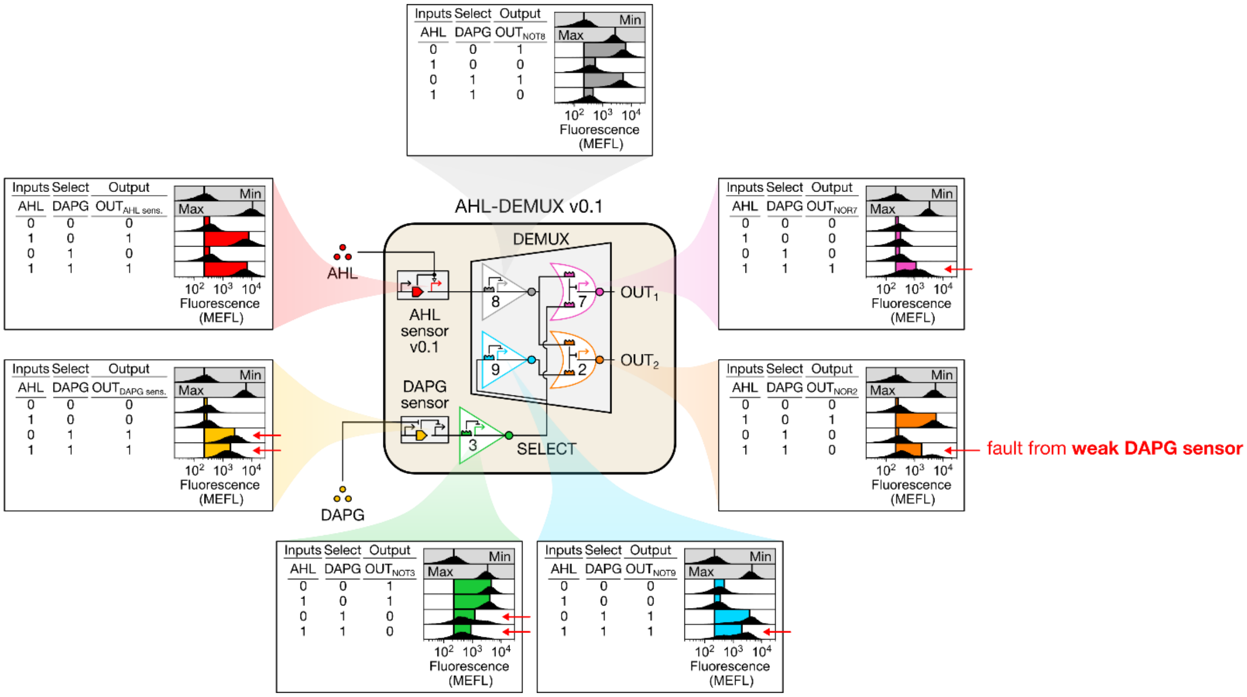


**Appendix Figure S9. DAPG sensor is too weak to control SELECT in preliminary AHL-DEMUX.** A preliminary AHL-DEMUX design (“AHL-DEMUX v0.1”) was constructed from the DEMUX by replacing the $P_R$ input promoter with an AHL sensor and expressing S3 from the DAPG sensor. A constitutive mCherry expression cassette was also incorporated (not pictured) to fluorescently distinguish AHL-DEMUX cells. The AHL-DEMUX v0.1 circuit plasmid was then separately co-transformed with the $P_{lux}$, $P_{PhlF}$, P2, P3, P7, P8, and P9 probe plasmids, and the resulting seven strains were incubated with the four possible binary combinations of AHL and DAPG (**Materials and Methods**). When DAPG is present, the activated DAPG sensor is unable to effectively repress NOT3 and generate SELECT = 0 (red arrows). This contradicts the NOT3 model, which predicts that both the max and observed DAPG sensor outputs should drive NOT3 low (e.g. NOT3(1500 MEFL) = 27 MEFL). Incomplete NOR3 repression leads to erroneous partial repression of $OUT_1$ and incomplete repression of $OUT_2$ in response to AHL (red arrows). Notably, similar faults do not arise in SENSOR-MUX-AHL, where the DAPG sensor effectively controls SELECT because it can repress NOT4 (**Fig. 4**), as predicted by the NOT4 model (e.g. NOT4(500 MEFL) = 38 MEFL). Min was measured once on one day, max was measured once on a second day, and all other measurements were performed on a third day. For sensors, max shows sensor output when the repressor is absent (DAPG sensor) or at maximum induction (AHL sensor). Inducer concentrations: 0 (0); 3.5 nM AHL, 100 µM DAPG (1).

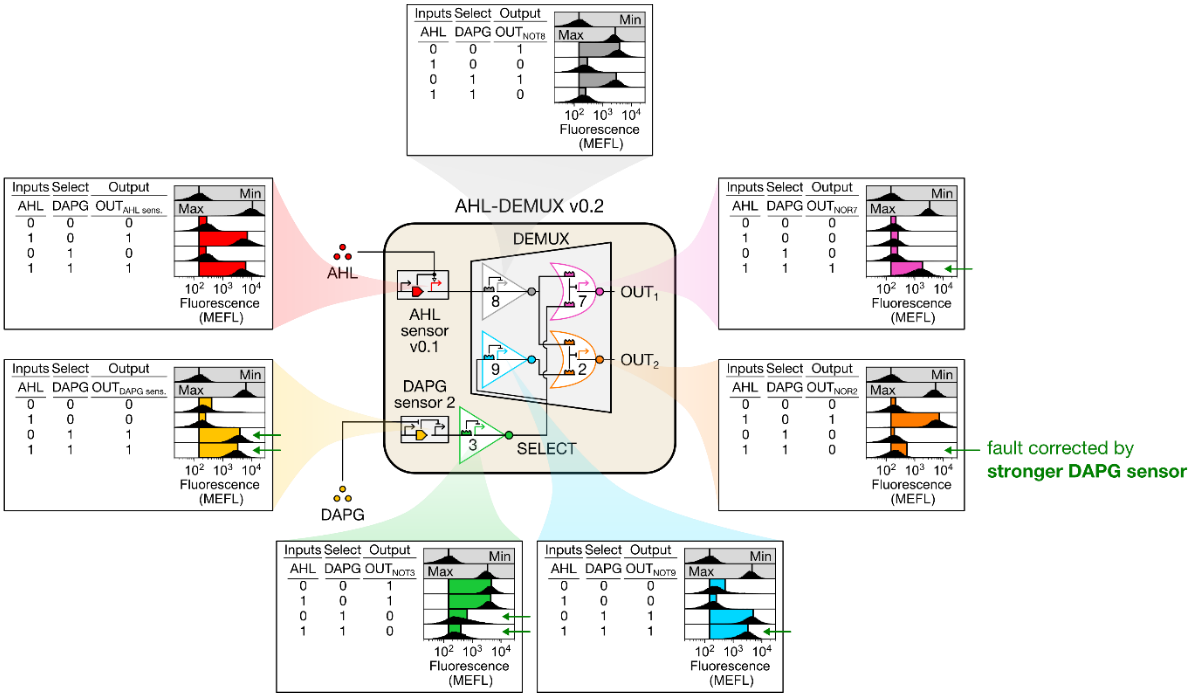


**Appendix Figure S10. Stronger DAPG sensor 2 correctly controls SELECT in AHL-DEMUX variant.** AHL-DEMUX v0.2 was constructed from AHL-DEMUX v0.1 by replacing the DAPG sensor with DAPG sensor 2 (**Appendix Table S3**). The AHL-DEMUX v0.2 circuit plasmid was then separately co-transformed with the $P_{lux}$, $P_{PhlF}$, P2, P3, P7, P8, and P9 probe plasmids, and the resulting seven strains were incubated with the four possible binary combinations of AHL and DAPG (**Materials and Methods**). When DAPG is present, DAPG sensor 2, whose output is ~1500 MEFL stronger than that of the previous DAPG sensor under the same conditions, achieves ~450 MEFL greater repression of NOT3, which proved sufficient to recover proper activation of $OUT_1$ and deactivation of $OUT_2$ in all cells (green arrows). Min was measured in triplicate on three separate days, max was measured once on a fourth day, and all other measurements were performed on a fifth day. For sensors, max shows sensor output when the repressor is absent (DAPG sensor) or at maximum induction (AHL sensor). Inducer concentrations: 0 (0); 3.5 nM AHL, 100µM DAPG (1).

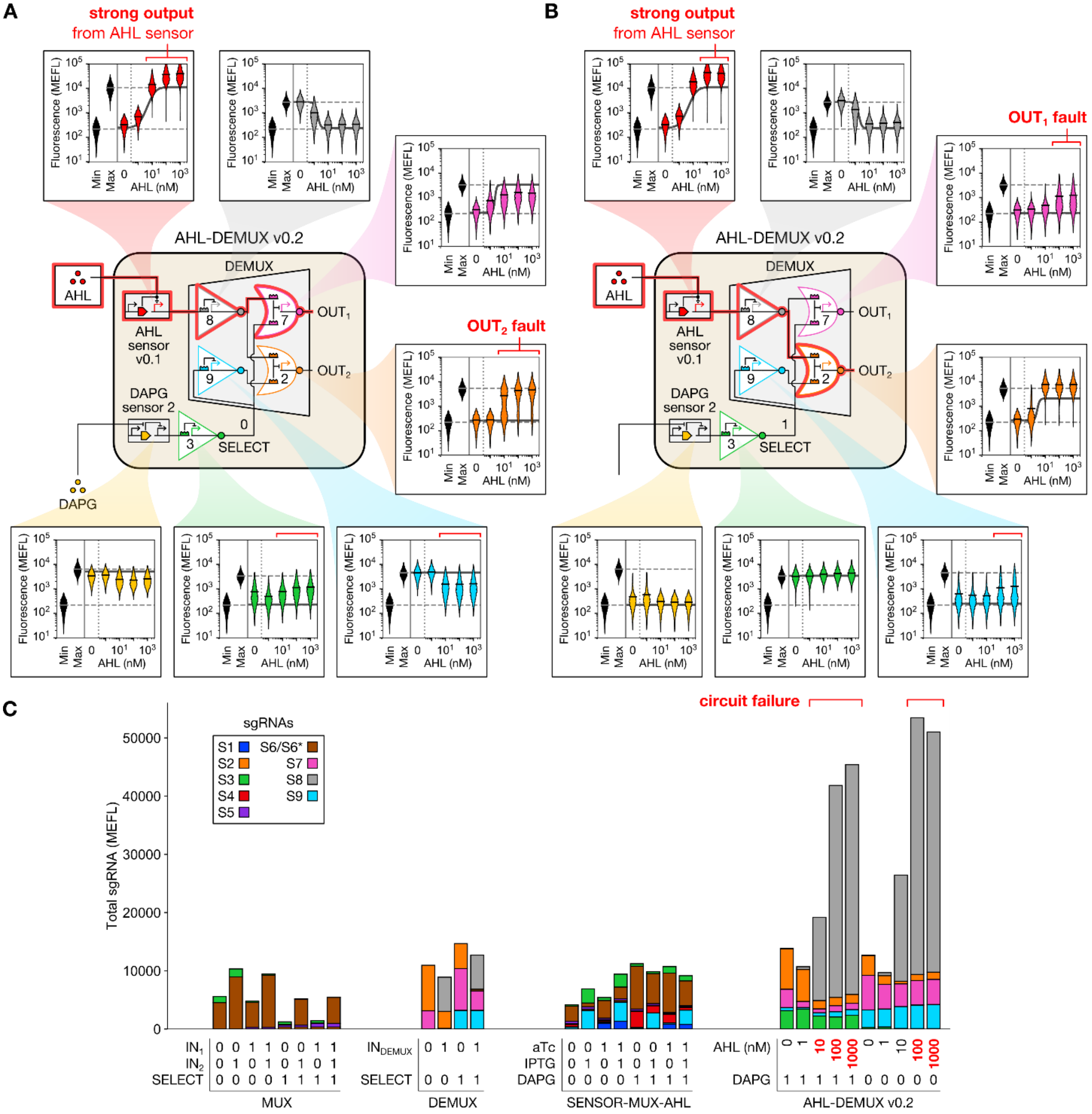


**Appendix Figure S11. Strong induction of AHL sensor causes output faults in AHL-DEMUX variant.** (**A**, **B**) Outputs of sensors and gates in AHL-DEMUX v0.2 upon induction with different AHL concentrations (0, 1, 10, 100, and 1000 nM) in the presence and absence of DAPG. In both cases, the correct AHL-DEMUX output activates in response to AHL, but at high AHL concentrations the incorrect output also activates ($OUT_2$ at 10, 100, and 1000 nM AHL when DAPG is present, and $OUT_1$ at 100 and 1000 nM AHL when DAPG is absent). Other gates also fail at high AHL concentrations (NOT3 and NOT9 when DAPG is present), and some failures are logically inconsistent (NOT9 and NOR7 relative to NOT3 when DAPG is absent). Min was measured once on one day, max was measured once on a second day, and all other measurements were performed on a third day. Solid dark gray lines show model predictions (made using the

$RECEIVER_{J23115}$ transfer function and the AHL concentration to predict $OUT_{AHL\ sensor}$, **Appendix Text S7**). For sensors, max shows sensor output when the repressor is absent (DAPG sensor) or at maximum induction (AHL sensor). (**C**) Total sgRNA produced by MUX, DEMUX, SENSOR-MUX-AHL, and AHL-DEMUX v0.2 under different input conditions. Total S*i* was calculated by summing the outputs of all sensors, gates, and constitutive promoters expressing S*i* (e.g. MUX S3 = $OUT_{NOR5}$ + $OUT_{NOR6}$) (**Table EV1**). Circuits expressing less than ~20,000 MEFL sgRNA function correctly, whereas circuits expressing more than ~25,000 MEFL exhibit systematic failure. Moreover, overexpressed S8 still effectively represses NOT8 in failing AHL-DEMUX v0.2 cells, while gates with lower but unchanging sgRNA levels suffer systematic activation as total expressed sgRNA increases (NOT3 when DAPG is present, and NOT9 and NOR7 when DAPG is absent). This suggests S8 may overwhelm dCas9 upon overexpression and that relative sgRNA abundance is an important factor influencing the effectiveness of multi-sgRNA CRISPRi.

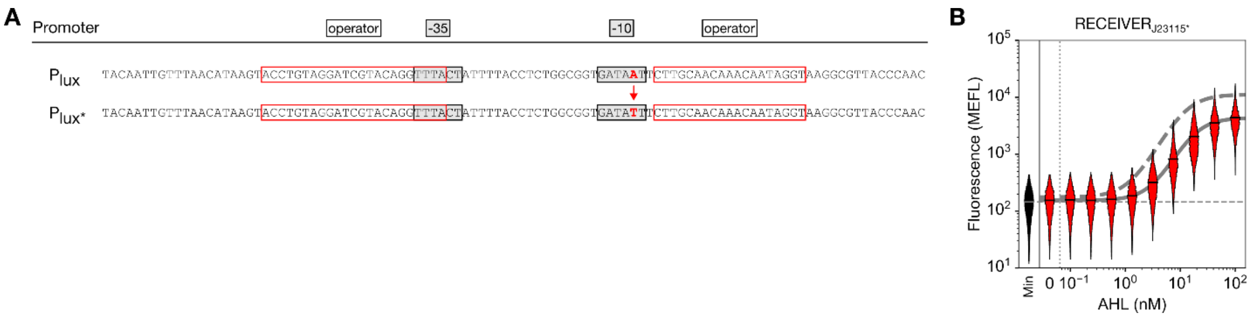


**Appendix Figure S12. Design and characterization of a reduced-strength AHL sensor.** (**A**) Sequence of $P_{lux}$ and its weaker variant $P_{lux*}$. (**B**) Transfer function of $RECEIVER_{J23115*}$. $RECEIVER_{J23115*}$ is identical to $RECEIVER_{J23115}$ except $P_{lux}$ was replaced with $P_{lux*}$ (**Appendix Fig. S6**). $P_{lux*}$ was selected from a small library of $P_{lux}$ variants containing single point mutations in their -35 or -10 regions based on its mildly reduced maximum output (~2.6-fold reduced) relative to $RECEIVER_{J23115}$ (dashed gray line). Min shows data combined from experiments on three separate days, and all other violins show data measured on a fourth day.

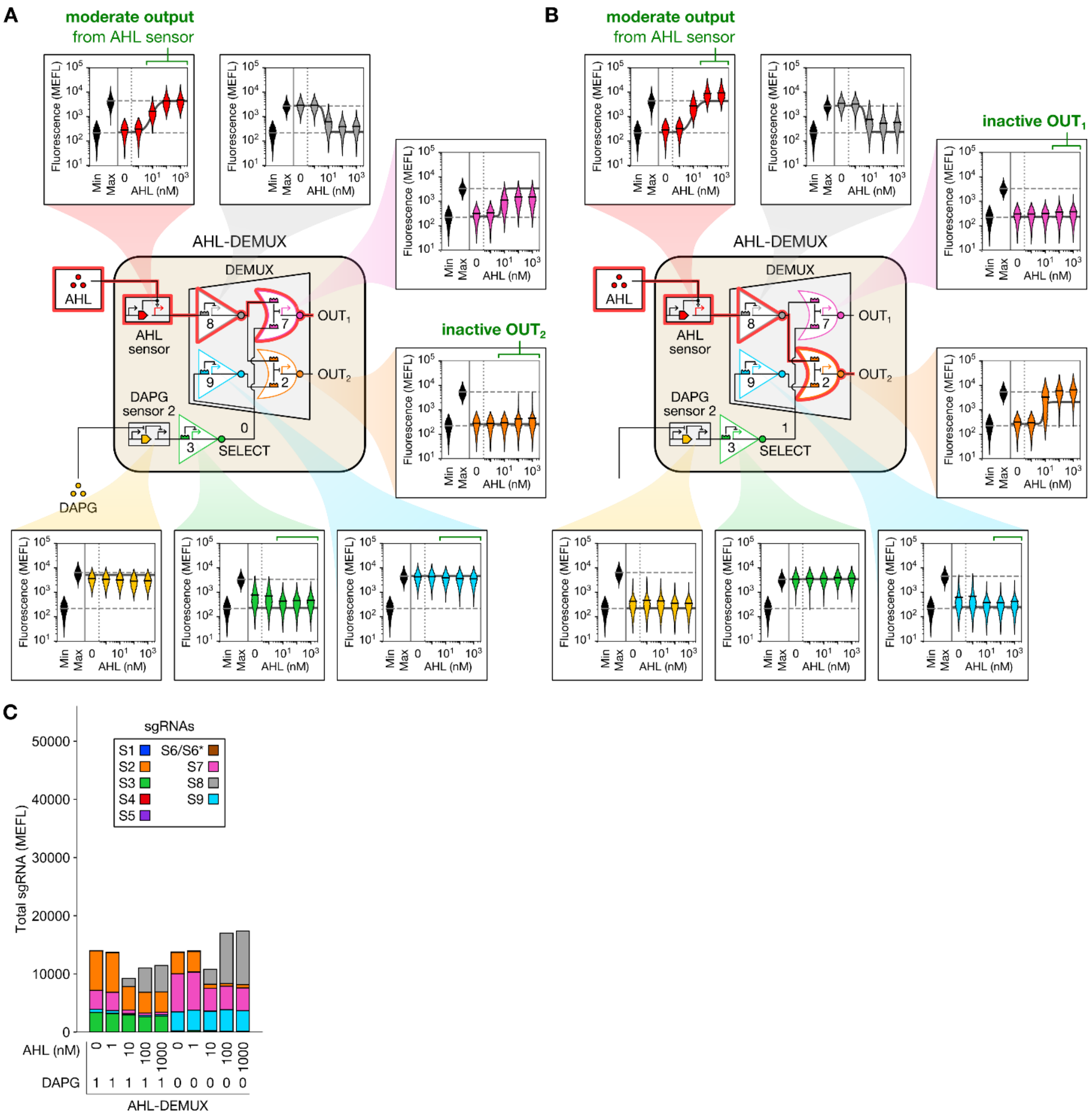


**Appendix Figure S13. Reduced-strength AHL sensor recovers robust activation of single AHL-DEMUX output in response to AHL.** (**A**, **B**) Outputs of sensors and gates in AHL-DEMUX upon induction with different AHL concentrations (0, 1, 10, 100, and 1000 nM) in the presence and absence of DAPG. AHL sensor output is reduced, faults identified with AHL-DEMUX v0.2 (**Appendix Fig. S11**) are corrected, and only the correct AHL-DEMUX output actives in response to AHL. Min was measured once on one day, max was measured once on a second day, and all other measurements were performed on a third day. Solid dark gray lines show model predictions (made using the RECEIVER$_{J23115*}$ transfer function and the AHL concentration to predict OUT$_{AHL\ sensor}$, **Appendix Text S7**). For sensors, max shows sensor output when the repressor is absent (DAPG sensor) or at maximum induction (AHL sensor). (**C**) Total sgRNA

produced by AHL-DEMUX under different input conditions. Total S*i* was calculated as in **Appendix Fig. S11C** (**Table EV1**). Total AHL-DEMUX sgRNA remains below ~20,000 MEFL and the circuit functions properly.

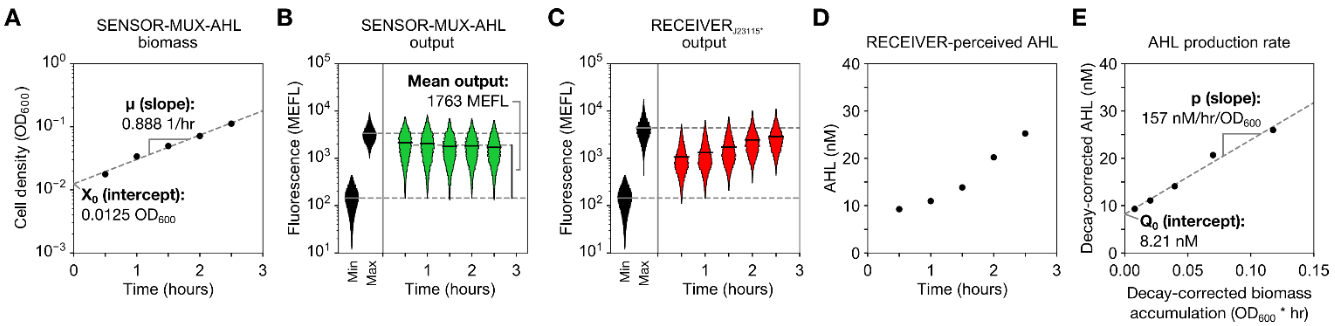


**Appendix Figure S14. Measuring the AHL production rate of LuxI in SENSOR-MUX-AHL cells.** SENSOR-MUX-AHL cells were (**A**) grown (**B**) expressing LuxI, and (**C**, **D**) their supernatant was periodically assayed with RECEIVER$_{J23115*}$ cells to measure AHL accumulation (**Materials and Methods**). The RECEIVER$_{J23115*}$ transfer function was used to calculate perceived AHL from mean RECEIVER$_{J23115*}$ sfGFP fluorescence (**Appendix Fig. S12B**, **Appendix Table S2**). (**E**) A model of AHL production and decay was assumed and then used with an exponential cell growth model to relate decay-corrected AHL concentration to decay-corrected biomass accumulation (**Appendix Text S9**). The slope of this plot (*p*) describes AHL production rate per cell (157 nM/hour/$OD_{600}$). *p* was then divided by mean SENSOR-MUX-AHL output (1763 MEFL) to calculate AHL production rate per LuxI expression ($s' = 0.089$ nM/hour/$OD_{600}$/MEFL, **Appendix Text S9**). Min shows data combined from experiments on three separate days, max shows data measured on a fourth day, and all other data were measured on a fifth day. For RECEIVER$_{J23115*}$, max shows output at maximum induction.

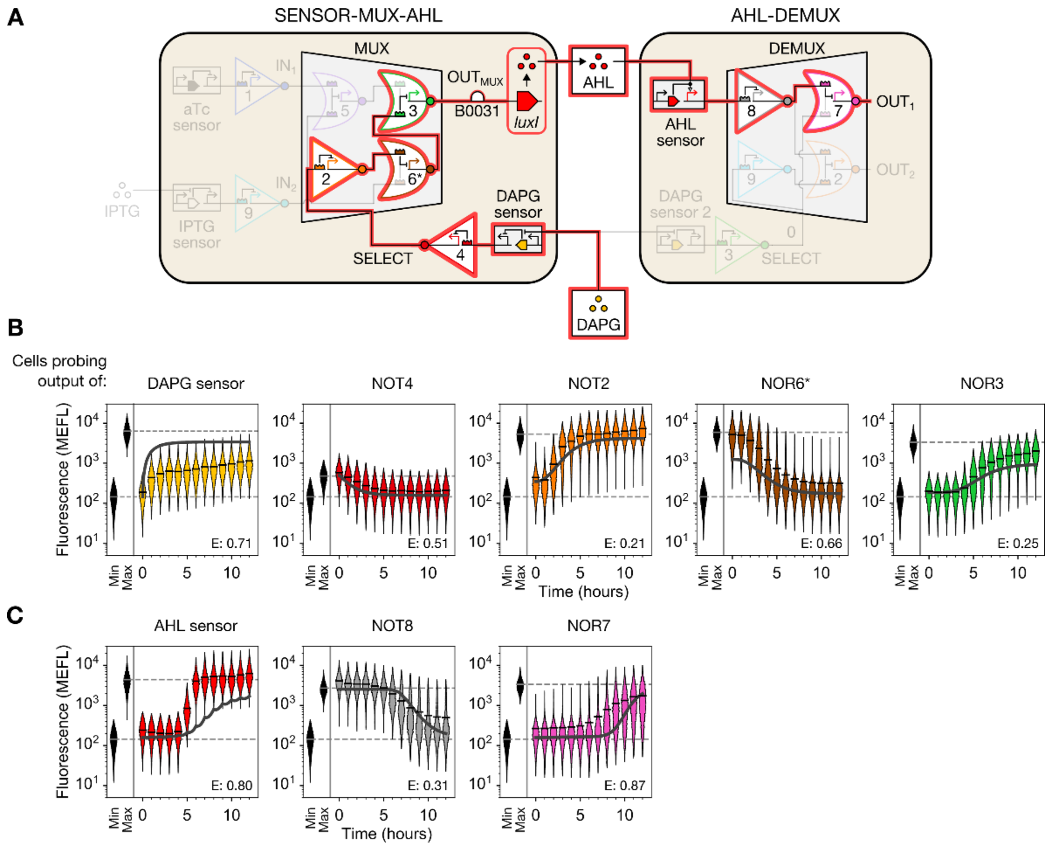


**Appendix Figure S15. Dynamical CS response to DAPG induction.** (**A**) Longest computation path through the CS. Dynamical responses of SENSOR-MUX-AHL (**B**) and AHL-DEMUX (**C**) sensors and gates to induction with DAPG in co-culture (**Materials and Methods**). Min shows data combined from experiments on three separate days, max shows data measured on a fourth day, and all other data were measured on a fifth day. For sensors, max shows sensor output when the repressor is absent (DAPG sensor) or at maximum induction (AHL sensor). Solid dark gray lines show model simulations (**Appendix Text S10**).

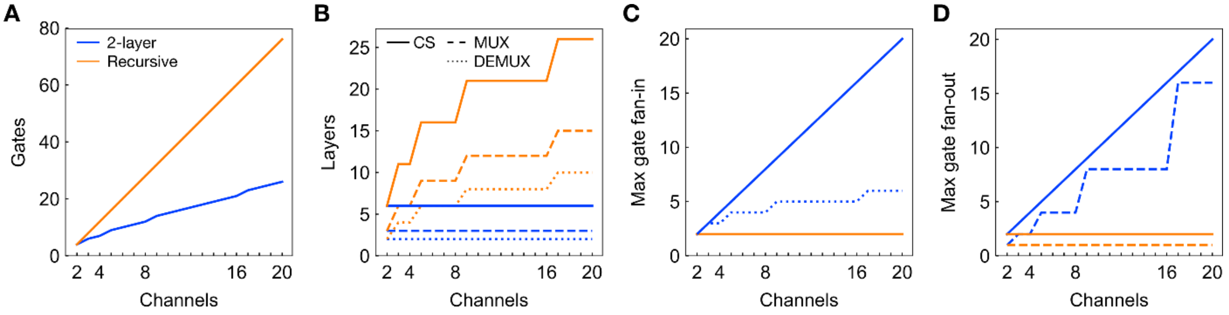


**Appendix Figure S16. CS scaling laws.** Larger MUX and DEMUX circuits can be constructed in many ways, each subject to different engineering tradeoffs. Two general approaches emerge, however, wherein larger circuits are constructed by linking smaller circuits of the same type ("Recursive", orange) or by connecting one layer of NOT gates to one (DEMUX) or two (MUX) layers of NOR gates ("2-layer", blue). While considerably fewer (**A**) gates (and therefore promoter:repressor pairs) and (**B**) layers are required for the 2-layer approach, the (**C**, **D**) maximum number of incoming (fan-in) and outgoing (fan-out) gate connections required quickly extends beyond those currently demonstrated for repressor-based gates in synthetic biology (which is three (Andrews *et al*, 2018) and two (Nielsen *et al*, 2016; Gander *et al*, 2017; Shin *et al*, 2020), respectively). While nothing fundamentally precludes higher gate fan-in and fan-out, larger CSs would likely combine both approaches (e.g. an 8-channel MUX via two 2-layer 4-channel MUXs connected to one 2-channel MUX, requiring 18 gates, 6 layers, and max gate fan-in and fan-out of 4 and 2, respectively). To achieve large circuits, multiple orthogonal gate technologies could be combined to increase the number of gates available in a single cell (e.g. CRISPRi, TetR homologs, transcription activator-like effector repressors (TALERs), and zinc-finger nucleases (ZFNs)), with upwards of 30 gates theoretically possible using libraries already published (Stanton *et al*, 2014; Zhang & Voigt, 2018). For plots, MUX and DEMUX relationships are only shown if they don't match the corresponding CS relationship.

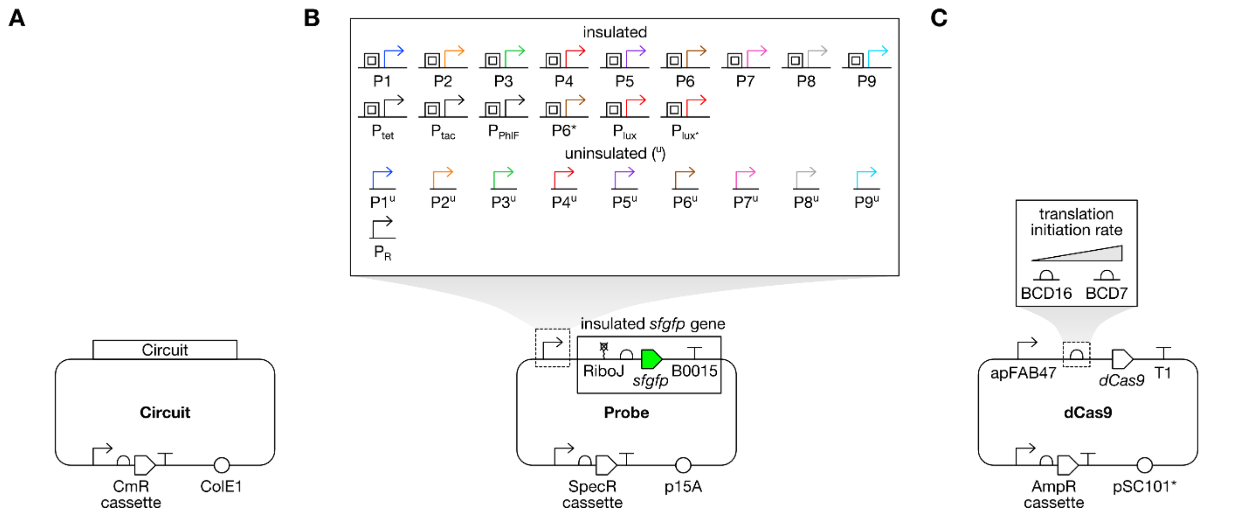


**Appendix Figure S17. Plasmid maps.** Schematics of (**A**) circuit, (**B**) probe, and (**C**) dCas9 plasmids used in this study. Probe plasmids harboring uninsulated P1-P9 and the dCas9 plasmid pSC31_1 harboring the weak RBS BCD16 were only used in the orthogonality assay. Not pictured: empty circuit and probe plasmids, which only contain antibiotic resistance cassettes and origins of replication, and a constitutive mCherry expression plasmid (pJS0205), wherein mCherry was expressed by P2 and a synthetic RBS. Circuit schematics are listed in **Appendix Fig. S18**.

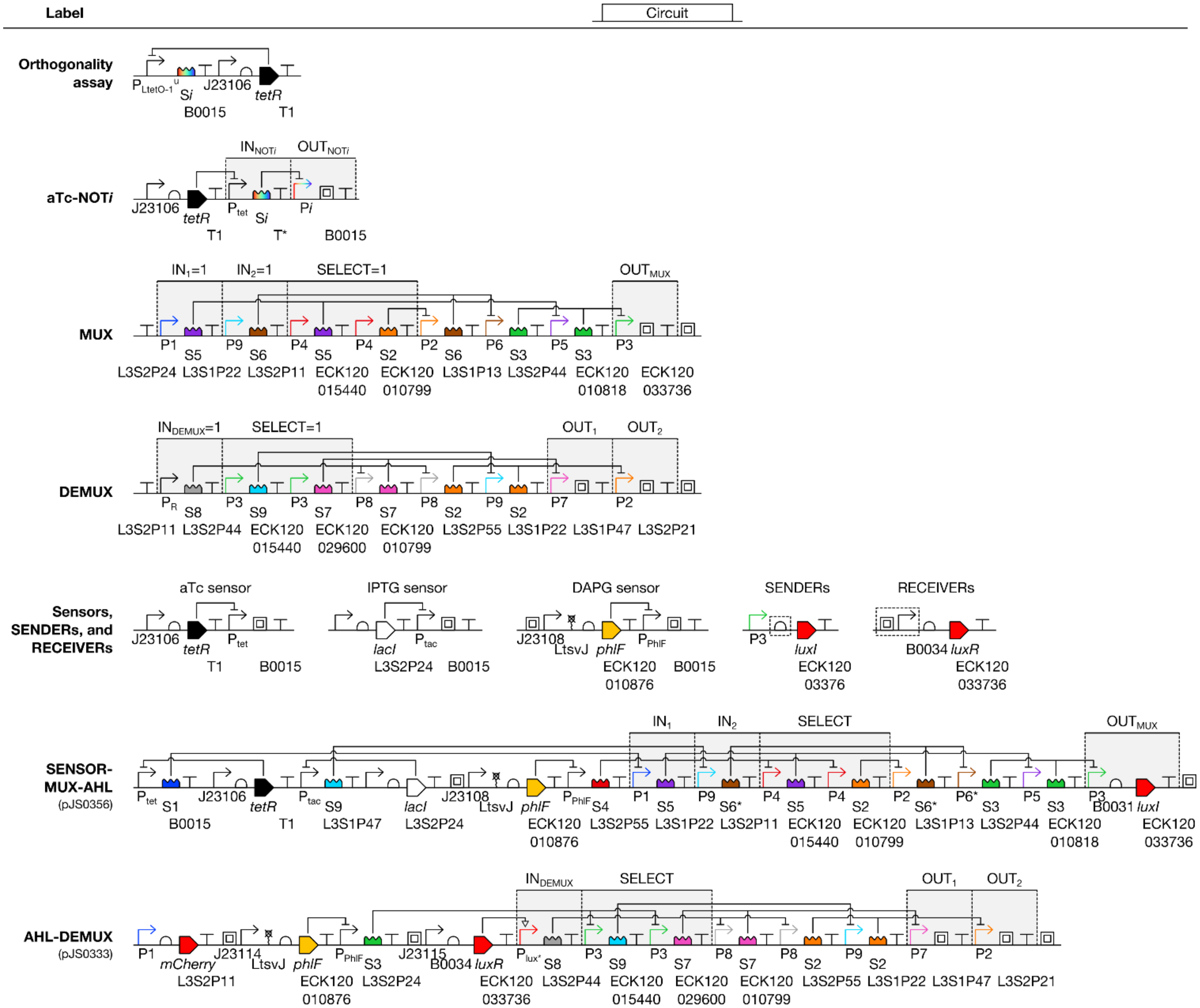


**Appendix Figure S18. Genetic device schematics.** Genetic circuits created in this study. Circuits were carried on circuit plasmids (**Appendix Fig. S17**). Transcription units relevant to signals discussed in the text are highlighted in gray. Additional MUX and DEMUX plasmids were constructed by omitting transcription units corresponding to different input signals. Additional preliminary SENSOR-MUX-AHL and AHL-DEMUX variants are listed in **Appendix Table S5**. SENDER RBSs and RECEIVER promoters (dashed boxes) were varied as discussed in **Appendix Fig. S6**. T* denotes a unique terminator from (Chen *et al*, 2013). $P_{tet}$ differs from $P_{LtetO\text{-}1}$ by an insulator.

# Appendix Tables

**Appendix Table S1. Gate transfer function model parameters.** Unconstrained Hill model parameter fits, standard errors (s.e.), and gate dynamic ranges.

| Gate | $Pi_{max}$ (MEFL) | $GATEi_{min}$ (MEFL) | s.e. (MEFL) | $K$ (MEFL) | s.e. (MEFL) | $n$ (unitless) | s.e. (unitless) | Dynamic range (fold change) |
|---|---|---|---|---|---|---|---|---|
| 1 | 292 | 21 | 36 | 17 | 14 | 0.69 | 0.54 | 13.9 |
| 2 | 5194 | 37 | 13 | 29 | 2.5 | 1.39 | 0.11 | 141.2 |
| 3 | 3208 | 8.7 | 20 | 48 | 13 | 1.49 | 0.36 | 364.0 |
| 4 | 335 | 9.2 | 18 | 65 | 28 | 1.15 | 0.57 | 35.8 |
| 5 | 1339 | 17 | 7.8 | 82 | 18 | 2.31 | 0.60 | 80.2 |
| 6 | 659 | 12 | 18 | 136 | 36 | 1.70 | 0.63 | 55.0 |
| 7 | 3213 | 11 | 16 | 146 | 30 | 1.98 | 0.37 | 294.8 |
| 8 | 2549 | 15 | 11 | 234 | 39 | 2.99 | 0.67 | 167.5 |
| 9 | 4398 | 29 | 12 | 339 | 32 | 4.29 | 0.75 | 151.3 |
| 6* | 5756 | 0 | † | 77 | † | 1.34 | † | Inf † |

† A constraint was encountered fitting NOT6*'s data ($GATE6*_{min}$=0), so no standard error estimates were calculated.

**Appendix Table S2. Sensor transfer function model parameters.** Activating Hill models ($y(x) = \min + ((\max - \min)/(1 + (K/x)^n))$) were fit to aTc, IPTG, DAPG, and AHL (RECEIVER$_{J23115}$ and RECEIVER$_{J23115*}$) sensor transfer function data (**Appendix Figs. S5, S6D, S12B**). Max and min are maximum and minimum mean sfGFP fluorescence produced by the sensor (MEFL), $K$ is ligand concentration at which the sensor is half activated (units depend on the ligand), and $n$ is the Hill coefficient (dimensionless), which describes the steepness of the transfer function. Parameter fits, their standard errors (s.e.), and sensor dynamic ranges are listed here.

| Sensor | max (MEFL) | s.e. | min (MEFL) | s.e. | $K$ | s.e. | units | $n$ (unitless) | s.e. | Dynamic range (fold change) |
|---|---|---|---|---|---|---|---|---|---|---|
| aTc | 1875 | 919 | 32 | 4.5 | 15 | 7.6 | ng/mL | 1.81 | 0.29 | 58.7 |
| IPTG | 19628 | 892 | 62 | 4.0 | 0.33 | 0.023 | mM | 2.14 | 0.081 | 317.1 |
| DAPG | 3147 | 355 | 86 | 13 | 20 | 2.8 | μM | 3.57 | 0.72 | 36.7 |
| RECEIVER$_{J23115}$ | 11037 | 510 | 32 | 1.3 | 12 | 0.80 | nM | 1.82 | 0.047 | 349.0 |
| RECEIVER$_{J23115*}$ | 4197 | 507 | 11 | 0.92 | 18 | 2.8 | nM | 1.88 | 0.11 | 390.6 |

**Appendix Table S3. DAPG sensor variants.** List of DAPG sensor variants characterized in this study. Sensor schematics match **Appendix Fig. S5C**.

| Label | *phlF* promoter | *phlF* promoter sequence | Efficacy |
|---|---|---|---|
| DAPG sensor | insulated BBa_J23108 | TGTAGAGTTATCCGCCTACGGC<br>GCCGTCGTATCGGTAATCCGTA<br>CGGGAATCGAAACGACGTCTAC<br>GAGCCTGACAGCTAGCTCAGTC<br>CTAGGTATAATGCTAGC | DAPG-responsive, near-maximally activating, not leaky |
| DAPG sensor 2 | insulated BBa_J23114 | TGTAGAGTTATCCGCCTACGGC<br>GCCGTCGTATCGGTAATCCGTA<br>CGGGAATCGAAACGACGTCTAC<br>GAGCTTTATGGCTAGCTCAGTC<br>CTAGGTACAATGCTAGC | DAPG-responsive, maximally activating, leaky in some contexts |
| none | insulated BBa_J23101 | TGTAGAGTTATCCGCCTACGGC<br>GCCGTCGTATCGGTAATCCGTA<br>CGGGAATCGAAACGACGTCTAC<br>GAGCTTTACAGCTAGCTCAGTC<br>CTAGGTATTATGCTAGC | unresponsive to DAPG, inactive |

**Appendix Table S4. Sequences of parts used in this study.** For compound parts, promoter = green, RBS = purple, protein coding sequence = blue, terminator = red. Terminator sequences can be found in (Chen *et al*, 2013). Annotated sequences are also available online through the Benchling platform (https://www.benchling.com/) and as GenBank files in **Dataset EV2**.

| Label | Type | Sequence | Reference |
|---|---|---|---|
| P1 | promoter | ACGTAGGGTAAGGAAGCGTAGTCGGGTCCGTTAAGGTTCG GTACGGACGGACCGCGTCGGTAGAGACTTCGACAATCGAT CATGCGATTGGTATAATAGATTCAT | This work |
| P2 | promoter | CGTTTTCGGGACGGATAAGGATTTCCTCCCGCGTAACCGT TTAATAACGGCGACCGTTACGCGAAGACTCGACAACAACG TGACAACACGGTATAATAGATTCAT | This work |
| P3 | promoter | GTTACCTTCCCGGAGGTAGCCGCGTTCCGCCCGAGTCGGA CGAGAACCGGGAGTCTTCGAAGGCTCTATCGACAATGTTG TGTTACGTTGGTATAATAGATTCAT | This work |
| P4 | promoter | GGTTCCCTTTTCCTCGTAAGGGCCGGGAACCGATTACGTC TCGGAGGGCCTCCGAGTCCTCGCGGTCTTCGACATCGATA ATGACACGCGGTATAATAGATTCAT | This work |
| P5 | promoter | GTTACGCGAAGGTAGGGAGAAGCGGTTTCCGCTTTAACCC GCCGGGACGTCTCGTAAACGTCGCGGGTTCGACATACGAC ACACGCAATGGTATAATAGATTCAT | This work |
| P6 | promoter | TATCGCTTCCGCGTTCCGTCTACTCCCTATTACGCTCCGG AATCTCCGACGCCTACGGCCGACGTCGGATTGACTATTGA TTACAACGTGTCGGATAATGGTTGC | This work |
| P7 | promoter | CTCCTTCGTAGATATCGTCCGGGTCCGCTTAAGCTCTTAC GTTCGTAGGAGTAAGACGACCCTCGGTATCGACATTGTGC GCGATCGACGGTATAATAGATTCAT | This work |
| P8 | promoter | CCTCTCCTTCGTCCCGAGAGTCCCGTTCGCGAAACGCCTC GATAACGAAAACGGAGCGAGGACTTCCCTCGACACGTGCG TTCACGTGTGGTATAATAGATTCAT | This work |
| P9 | promoter | TTTATTTATCGTCCGCCGGCCGACGGACTTCGTATTTCGG AGCTACTCGGGCGTATACGTATCGGGCCTCGACATGTCGT GCGTGTATTGGTATAATAGATTCAT | This work |
| P6* | promoter | TCGCTTCCGCGTTCCGTCTACTCCCTATTACGCTCCGGAA TCTCCGACGCCTACGGCCGACGTCGGAAAAAAATTTATTT GCTTTGATTACAACGTGTCTGGTATAATGTGTGGAT | This work |
| $P_R$ | promoter | TAACACCGTGCGTGTTGACTATTTTACCTCTGGCGGTGAT AATGGTTGC | (Ptashne, 2004) |
| $P_{tet}$ | promoter | GGTAGTAGCTCGGGAGTCCTTTTCCGGATTTCTATCCGGG CCTTCGACGGATCCCTATCAGTGATAGAGATTGACATCCC TATCAGTGATAGAGATACTGAGCAC | Modified from (Lutz & Bujard, 1997) |
| $P_{tac}$ | promoter | TTTCGATAAACTTCGGGACGGGATAGCTCTCCTACCTTTC TCTTAAGCTCGAATACCTCGAGGCCCGCCCTTGACAATTA ATCATCGGCTCGTATAATGTGTGGAATTGTGAGCGGATAA CAATTTCACACA | Modified from (de Boer *et al*, 1983) |
| $P_{PhlF}$ | promoter | CGACGTACGGTGGAATCTGATTCGTTACCAATTGACATGA TACGAAACGTACCGTATCGTTAAGGT | (Nielsen & Voigt, 2014) |
| $P_{lux}$ | promoter | TACAATTGTTTAACATAAGTACCTGTAGGATCGTACAGGT TTACTATTTTACCTCTGGCGGTGATAATTCTTGCAACAAA CAATAGGTAAGGCGTTACCCAAC | D49 from (Cox *et al*, 2007) |
| $P_{lux*}$ | promoter | TACAATTGTTTAACATAAGTACCTGTAGGATCGTACAGGT TTACTATTTTACCTCTGGCGGTGATATTTCTTGCAACAAA CAATAGGTAAGGCGTTACCCAAC | Modified from (Cox *et al*, 2007) |

| | | | |
|---|---|---|---|
| S1 | sgRNA | TCGACAATCGATCATGCGATGTTTTAGAGCTAGAAATAGC<br>AAGTTAAAATAAGGCTAGTCCGTTATCAACTTGAAAAAGT<br>GGCACCGAGTCGGTGCTTTTTT | This work |
| S2 | sgRNA | TCGACAACAACGTGACAACAGTTTTAGAGCTAGAAATAGC<br>AAGTTAAAATAAGGCTAGTCCGTTATCAACTTGAAAAAGT<br>GGCACCGAGTCGGTGCTTTTTT | This work |
| S3 | sgRNA | TCGACAATGTTGTGTTACGTGTTTTAGAGCTAGAAATAGC<br>AAGTTAAAATAAGGCTAGTCCGTTATCAACTTGAAAAAGT<br>GGCACCGAGTCGGTGCTTTTTT | This work |
| S4 | sgRNA | TCGACATCGATAATGACACGGTTTTAGAGCTAGAAATAGC<br>AAGTTAAAATAAGGCTAGTCCGTTATCAACTTGAAAAAGT<br>GGCACCGAGTCGGTGCTTTTTT | This work |
| S5 | sgRNA | TCGACATACGACACACGCAAGTTTTAGAGCTAGAAATAGC<br>AAGTTAAAATAAGGCTAGTCCGTTATCAACTTGAAAAAGT<br>GGCACCGAGTCGGTGCTTTTTT | This work |
| S6 | sgRNA | GACTATTGATTACAACGTGTGTTTTAGAGCTAGAAATAGC<br>AAGTTAAAATAAGGCTAGTCCGTTATCAACTTGAAAAAGT<br>GGCACCGAGTCGGTGCTTTTTT | This work |
| S7 | sgRNA | TCGACATTGTGCGCGATCGAGTTTTAGAGCTAGAAATAGC<br>AAGTTAAAATAAGGCTAGTCCGTTATCAACTTGAAAAAGT<br>GGCACCGAGTCGGTGCTTTTTT | This work |
| S8 | sgRNA | TCGACACGTGCGTTCACGTGGTTTTAGAGCTAGAAATAGC<br>AAGTTAAAATAAGGCTAGTCCGTTATCAACTTGAAAAAGT<br>GGCACCGAGTCGGTGCTTTTTT | This work |
| S9 | sgRNA | TCGACATGTCGTGCGTGTATGTTTTAGAGCTAGAAATAGC<br>AAGTTAAAATAAGGCTAGTCCGTTATCAACTTGAAAAAGT<br>GGCACCGAGTCGGTGCTTTTTT | This work |
| S6* | sgRNA | TGCTTTGATTACAACGTGTCGTTTTAGAGCTAGAAATAGC<br>AAGTTAAAATAAGGCTAGTCCGTTATCAACTTGAAAAAGT<br>GGCACCGAGTCGGTGCTTTTTT | This work |
| *dcas9* (weak) | transcription unit | AAAAAGAGTATTGACTTCGCATCTTTTTGTACCCATAATT<br>ATTTCATGGGCCCAAGTTCACTTAAAAAGGAGATCAACAA<br>TGAAAGCAATTTTCGTACTGAAACATCTTAATCATGCTTA<br>GGAGTCTTTCTAATGGATAAGAAATACTCAATAGGCTTAG<br>CTATCGGCACAAATAGCGTCGGATGGGCGGTGATCACTGA<br>TGAATATAAGGTTCCGTCTAAAAAGTTCAAGGTTCTGGGA<br>AATACAGACCGCCACAGTATCAAAAAAAATCTTATAGGGG<br>CTCTTTTATTTGACAGTGGAGAGACAGCGGAAGCGACTCG<br>TCTCAAACGGACAGCTCGTAGAAGGTATACACGTCGGAAG<br>AATCGTATTTGTTATCTACAGGAGATTTTTTCAAATGAGA<br>TGGCGAAAGTAGATGATAGTTTCTTTCATCGACTTGAAGA<br>GTCTTTTTTGGTGGAAGAAGACAAGAAGCATGAACGTCAT<br>CCTATTTTTGGAAATATAGTAGATGAAGTTGCTTATCATG<br>AGAAATATCCAACTATCTATCATCTGCGAAAAAAATTGGT<br>AGATTCTACTGATAAAGCGGATTTGCGCTTAATCTATTTG<br>GCCTTAGCGCATATGATTAAGTTTCGTGGTCATTTTTTGA<br>TTGAGGGAGATTTAAATCCTGATAATAGTGATGTGGACAA<br>ACTATTTATCCAGTTGGTACAAACCTACAATCAATTATTT<br>GAAGAAAACCCTATTAACGCAAGTGGAGTAGATGCTAAAG<br>CGATTCTTTCTGCACGATTGAGTAAATCAAGACGATTAGA<br>AAATCTCATTGCTCAGCTCCCCGGTGAGAAGAAAAATGGC<br>TTATTTGGGAATCTCATTGCTTTGTCATTGGGTTTGACCC<br>CTAATTTTAAATCAAATTTTGATTTGGCAGAAGATGCTAA<br>ATTACAGCTTTCAAAAGATACTTACGATGATGATTTAGAT<br>AATTTATTGGCGCAAATTGGAGATCAATATGCTGATTTGT<br>TTTTGGCAGCTAAGAATTTATCAGATGCTATTTTACTTTC<br>AGATATCCTAAGAGTAAATACTGAAATAACTAAGGCTCCC | Modified from (Qi *et al*, 2013) |

CTATCAGCTTCAATGATTAAACGCTACGATGAACATCATC
AAGACTTGACTCTTTTAAAAGCTTTAGTTCGACAACAACT
TCCAGAAAAGTATAAAGAAATCTTTTTTGATCAATCAAAA
AACGGATATGCAGGTTATATTGATGGGGGAGCTAGCCAAG
AAGAATTTTATAAATTTATCAAACCAATTTTAGAAAAAAT
GGATGGTACTGAGGAATTATTGGTGAAACTAAATCGTGAA
GATTTGCTGCGCAAGCAACGGACCTTTGACAACGGCTCTA
TTCCCCATCAAATTCACTTGGGTGAGCTGCATGCTATTTT
GAGAAGACAAGAAGACTTTTATCCATTTTTAAAAGACAAT
CGTGAGAAGATTGAAAAAATCTTGACTTTTCGAATTCCTT
ATTATGTTGGTCCATTGGCGCGTGGCAATAGTCGTTTTGC
ATGGATGACTCGGAAGTCTGAAGAAACAATTACCCCATGG
AATTTTGAAGAAGTTGTCGATAAAGGTGCTTCAGCTCAAT
CATTTATTGAACGCATGACAAACTTTGATAAAAATCTTCC
AAATGAAAAAGTACTACCAAAACATAGTTTGCTTTATGAG
TATTTTACGGTTTATAACGAATTGACAAAGGTCAAATATG
TTACTGAAGGAATGCGAAAACCAGCATTTCTTTCAGGTGA
ACAGAAGAAAGCCATTGTTGATTTACTCTTCAAAACAAAT
CGAAAAGTAACCGTTAAGCAATTAAAAGAAGATTATTTCA
AAAAAATAGAATGTTTTGATAGTGTTGAAATTTCAGGAGT
TGAAGATAGATTTAATGCTTCATTAGGTACCTACCATGAT
TTGCTAAAAATTATTAAAGATAAAGATTTTTTGGATAATG
AAGAAAATGAAGATATCTTAGAGGATATTGTTTTAACATT
GACCTTATTTGAAGATAGGGAGATGATTGAGGAAAGACTT
AAAACATATGCTCACCTCTTTGATGATAAGGTGATGAAAC
AGCTTAAACGTCGCCGTTATACTGGTTGGGGACGTTTGTC
TCGAAAATTGATTAATGGTATTAGGGATAAGCAATCTGGC
AAAACAATATTAGATTTTTTGAAATCAGATGGTTTTGCCA
ATCGCAATTTTATGCAGCTGATCCATGATGATAGTTTGAC
ATTTAAAGAAGACATTCAAAAAGCACAAGTGTCTGGACAA
GGCGATAGTTTACATGAACATATTGCAAATTTAGCTGGTA
GCCCTGCTATTAAAAAAGGTATTTTACAGACTGTAAAAGT
TGTTGATGAATTGGTCAAAGTAATGGGGCGGCATAAGCCA
GAAAATATCGTTATTGAAATGGCACGTGAAAATCAGACAA
CTCAAAAGGGCCAGAAAAATTCGCGAGAGCGTATGAAACG
AATCGAAGAAGGTATCAAAGAATTAGGAAGTCAGATTCTT
AAAGAGCATCCTGTTGAAAATACTCAATTGCAAAATGAAA
AGCTCTATCTCTATTATCTCCAAAATGGAAGAGACATGTA
TGTGGACCAAGAATTAGATATTAATCGTTTAAGTGATTAT
GATGTCGATGCCATTGTTCCACAAAGTTTCCTTAAAGACG
ATTCAATAGACAATAAGGTCTTAACGCGTTCTGATAAAAA
TCGTGGTAAATCGGATAACGTTCCAAGTGAAGAAGTAGTC
AAAAAGATGAAAAACTATTGGAGACAACTTCTAAACGCCA
AGTTAATCACTCAACGTAAGTTTGATAATTTAACGAAAGC
TGAACGTGGAGGTTTGAGTGAACTTGATAAAGCTGGTTTT
ATCAAACGCCAATTGGTTGAAACTCGCCAAATCACTAAGC
ATGTGGCACAAATTTTGGATAGTCGCATGAATACTAAATA
CGATGAAAATGATAAACTTATTCGAGAGGTTAAAGTGATT
ACCTTAAAATCTAAATTAGTTTCTGACTTCCGAAAAGATT
TCCAATTCTATAAAGTACGTGAGATTAACAATTACCATCA
TGCCCATGATGCGTATCTAAATGCCGTCGTTGGAACTGCT
TTGATTAAGAAATATCCAAAACTTGAATCGGAGTTTGTCT
ATGGTGATTATAAAGTTTATGATGTTCGTAAAATGATTGC
TAAGTCTGAGCAAGAAATAGGCAAAGCAACCGCAAAATAT
TTCTTTTACTCTAATATCATGAACTTCTTCAAAACAGAAA
TTACACTTGCAAATGGAGAGATTCGCAAACGCCCTCTAAT
CGAAACTAATGGGGAAACTGGAGAAATTGTCTGGGATAAA

| | | | |
|---|---|---|---|
| | | GGGCGAGATTTTGCCACAGTGCGCAAAGTATTGTCCATGC<br>CCCAAGTCAATATTGTCAAGAAAACAGAAGTACAGACAGG<br>CGGATTCTCCAAGGAGTCAATTTTACCAAAAAGAAATTCG<br>GACAAGCTTATTGCTCGTAAAAAAGACTGGGATCCAAAAA<br>AATATGGTGGTTTTGATAGTCCAACGGTAGCTTATTCAGT<br>CCTAGTGGTTGCTAAGGTGGAAAAAGGGAAATCGAAGAAG<br>TTAAAATCCGTTAAAGAGTTACTAGGGATCACAATTATGG<br>AAAGAAGTTCCTTTGAAAAAAATCCGATTGACTTTTTAGA<br>AGCTAAAGGATATAAGGAAGTTAAAAAAGACTTAATCATT<br>AAACTACCTAAATATAGTCTTTTTGAGTTAGAAAACGGTC<br>GTAAACGGATGCTGGCTAGTGCCGGAGAATTACAAAAAGG<br>AAATGAGCTGGCTCTGCCAAGCAAATATGTGAATTTTTTA<br>TATTTAGCTAGTCATTATGAAAAGTTGAAGGGTAGTCCAG<br>AAGATAACGAACAAAAACAATTGTTTGTGGAGCAGCATAA<br>GCATTATTTAGATGAGATTATTGAGCAAATCAGTGAATTT<br>TCTAAGCGTGTTATTTTAGCAGATGCCAATTTAGATAAAG<br>TTCTTAGTGCATATAACAAACATAGAGACAAACCAATACG<br>TGAACAAGCAGAAAATATTATTCATTTATTTACGTTGACG<br>AATCTTGGAGCTCCCGCTGCTTTTAAATATTTTGATACAA<br>CAATTGATCGTAAACGATATACGTCTACAAAAGAAGTTTT<br>AGATGCCACTCTTATCCATCAATCCATCACTGGTCTTTAT<br>GAAACACGCATTGATTTGAGTCAGCTAGGAGGTGACTGAG<br>GCATCAAATAAAACGAAAGGCTCAGTCGAAAGACTGGGCC<br>TTTCGTTTTATCTGTTGTTTGTCGGTGAACGCTCTCCTGA<br>GTAGGACAAATCCGCCGCCCTAGA | |
| *dcas9* (strong) | transcription unit | AAAAAGAGTATTGACTTCGCATCTTTTTGTACCCATAATT<br>ATTTCATGGGCCCAAGTTCACTTAAAAAGGAGATCAACAA<br>TGAAAGCAATTTTCGTACTGAAACATCTTAATCATGCTGG<br>GGAGGGTTTCTAATGGATAAGAAATACTCAATAGGCTTAG<br>CTATCGGCACAAATAGCGTCGGATGGGCGGTGATCACTGA<br>TGAATATAAGGTTCCGTCTAAAAAGTTCAAGGTTCTGGGA<br>AATACAGACCGCCACAGTATCAAAAAAAATCTTATAGGGG<br>CTCTTTTATTTGACAGTGGAGAGACAGCGGAAGCGACTCG<br>TCTCAAACGGACAGCTCGTAGAAGGTATACACGTCGGAAG<br>AATCGTATTTGTTATCTACAGGAGATTTTTTCAAATGAGA<br>TGGCGAAAGTAGATGATAGTTTCTTTCATCGACTTGAAGA<br>GTCTTTTTTGGTGGAAGAAGACAAGAAGCATGAACGTCAT<br>CCTATTTTTGGAAATATAGTAGATGAAGTTGCTTATCATG<br>AGAAATATCCAACTATCTATCATCTGCGAAAAAAATTGGT<br>AGATTCTACTGATAAAGCGGATTTGCGCTTAATCTATTTG<br>GCCTTAGCGCATATGATTAAGTTTCGTGGTCATTTTTTGA<br>TTGAGGGAGATTTAAATCCTGATAATAGTGATGTGGACAA<br>ACTATTTATCCAGTTGGTACAAACCTACAATCAATTATTT<br>GAAGAAAACCCTATTAACGCAAGTGGAGTAGATGCTAAAG<br>CGATTCTTTCTGCACGATTGAGTAAATCAAGACGATTAGA<br>AAATCTCATTGCTCAGCTCCCCGGTGAGAAGAAAAATGGC<br>TTATTTGGGAATCTCATTGCTTTGTCATTGGGTTTGACCC<br>CTAATTTTAAATCAAATTTTGATTTGGCAGAAGATGCTAA<br>ATTACAGCTTTCAAAAGATACTTACGATGATGATTTAGAT<br>AATTTATTGGCGCAAATTGGAGATCAATATGCTGATTTGT<br>TTTTGGCAGCTAAGAATTTATCAGATGCTATTTTACTTTC<br>AGATATCCTAAGAGTAAATACTGAAATAACTAAGGCTCCC<br>CTATCAGCTTCAATGATTAAACGCTACGATGAACATCATC<br>AAGACTTGACTCTTTTAAAAGCTTTAGTTCGACAACAACT<br>TCCAGAAAAGTATAAAGAAATCTTTTTTGATCAATCAAAA<br>AACGGATATGCAGGTTATATTGATGGGGGAGCTAGCCAAG<br>AAGAATTTTATAAATTTATCAAACCAATTTTAGAAAAAAT | Modified from (Qi *et al*, 2013) |

GGATGGTACTGAGGAATTATTGGTGAAACTAAATCGTGAA
GATTTGCTGCGCAAGCAACGGACCTTTGACAACGGCTCTA
TTCCCCATCAAATTCACTTGGGTGAGCTGCATGCTATTTT
GAGAAGACAAGAAGACTTTTATCCATTTTTAAAAGACAAT
CGTGAGAAGATTGAAAAAATCTTGACTTTTCGAATTCCTT
ATTATGTTGGTCCATTGGCGCGTGGCAATAGTCGTTTTGC
ATGGATGACTCGGAAGTCTGAAGAAACAATTACCCCATGG
AATTTTGAAGAAGTTGTCGATAAAGGTGCTTCAGCTCAAT
CATTTATTGAACGCATGACAAACTTTGATAAAAATCTTCC
AAATGAAAAAGTACTACCAAAACATAGTTTGCTTTATGAG
TATTTTACGGTTTATAACGAATTGACAAAGGTCAAATATG
TTACTGAAGGAATGCGAAAACCAGCATTTCTTTCAGGTGA
ACAGAAGAAAGCCATTGTTGATTTACTCTTCAAAACAAAT
CGAAAAGTAACCGTTAAGCAATTAAAAGAAGATTATTTCA
AAAAAATAGAATGTTTTGATAGTGTTGAAATTTCAGGAGT
TGAAGATAGATTTAATGCTTCATTAGGTACCTACCATGAT
TTGCTAAAAATTATTAAAGATAAAGATTTTTTGGATAATG
AAGAAAATGAAGATATCTTAGAGGATATTGTTTTAACATT
GACCTTATTTGAAGATAGGGAGATGATTGAGGAAAGACTT
AAAACATATGCTCACCTCTTTGATGATAAGGTGATGAAAC
AGCTTAAACGTCGCCGTTATACTGGTTGGGGACGTTTGTC
TCGAAAATTGATTAATGGTATTAGGGATAAGCAATCTGGC
AAAACAATATTAGATTTTTTGAAATCAGATGGTTTTGCCA
ATCGCAATTTTATGCAGCTGATCCATGATGATAGTTTGAC
ATTTAAAGAAGACATTCAAAAAGCACAAGTGTCTGGACAA
GGCGATAGTTTACATGAACATATTGCAAATTTAGCTGGTA
GCCCTGCTATTAAAAAAGGTATTTTACAGACTGTAAAAGT
TGTTGATGAATTGGTCAAAGTAATGGGGCGGCATAAGCCA
GAAAATATCGTTATTGAAATGGCACGTGAAAATCAGACAA
CTCAAAAGGGCCAGAAAAATTCGCGAGAGCGTATGAAACG
AATCGAAGAAGGTATCAAAGAATTAGGAAGTCAGATTCTT
AAAGAGCATCCTGTTGAAAATACTCAATTGCAAAATGAAA
AGCTCTATCTCTATTATCTCCAAAATGGAAGAGACATGTA
TGTGGACCAAGAATTAGATATTAATCGTTTAAGTGATTAT
GATGTCGATGCCATTGTTCCACAAAGTTTCCTTAAAGACG
ATTCAATAGACAATAAGGTCTTAACGCGTTCTGATAAAAA
TCGTGGTAAATCGGATAACGTTCCAAGTGAAGAAGTAGTC
AAAAAGATGAAAAACTATTGGAGACAACTTCTAAACGCCA
AGTTAATCACTCAACGTAAGTTTGATAATTTAACGAAAGC
TGAACGTGGAGGTTTGAGTGAACTTGATAAAGCTGGTTTT
ATCAAACGCCAATTGGTTGAAACTCGCCAAATCACTAAGC
ATGTGGCACAAATTTTGGATAGTCGCATGAATACTAAATA
CGATGAAAATGATAAACTTATTCGAGAGGTTAAAGTGATT
ACCTTAAAATCTAAATTAGTTTCTGACTTCCGAAAAGATT
TCCAATTCTATAAAGTACGTGAGATTAACAATTACCATCA
TGCCCATGATGCGTATCTAAATGCCGTCGTTGGAACTGCT
TTGATTAAGAAATATCCAAAACTTGAATCGGAGTTTGTCT
ATGGTGATTATAAAGTTTATGATGTTCGTAAAATGATTGC
TAAGTCTGAGCAAGAAATAGGCAAAGCAACCGCAAAATAT
TTCTTTTACTCTAATATCATGAACTTCTTCAAAACAGAAA
TTACACTTGCAAATGGAGAGATTCGCAAACGCCCTCTAAT
CGAAACTAATGGGGAAACTGGAGAAATTGTCTGGGATAAA
GGGCGAGATTTTGCCACAGTGCGCAAAGTATTGTCCATGC
CCCAAGTCAATATTGTCAAGAAAACAGAAGTACAGACAGG
CGGATTCTCCAAGGAGTCAATTTTACCAAAAAGAAATTCG
GACAAGCTTATTGCTCGTAAAAAAGACTGGGATCCAAAAA
AATATGGTGGTTTTGATAGTCCAACGGTAGCTTATTCAGT

| | | | |
|---|---|---|---|
| | | CCTAGTGGTTGCTAAGGTGGAAAAAGGGAAATCGAAGAAG<br>TTAAAATCCGTTAAAGAGTTACTAGGGATCACAATTATGG<br>AAAGAAGTTCCTTTGAAAAAAATCCGATTGACTTTTTAGA<br>AGCTAAAGGATATAAGGAAGTTAAAAAAGACTTAATCATT<br>AAACTACCTAAATATAGTCTTTTTGAGTTAGAAAACGGTC<br>GTAAACGGATGCTGGCTAGTGCCGGAGAATTACAAAAAGG<br>AAATGAGCTGGCTCTGCCAAGCAAATATGTGAATTTTTTA<br>TATTTAGCTAGTCATTATGAAAAGTTGAAGGGTAGTCCAG<br>AAGATAACGAACAAAAACAATTGTTTGTGGAGCAGCATAA<br>GCATTATTTAGATGAGATTATTGAGCAAATCAGTGAATTT<br>TCTAAGCGTGTTATTTTAGCAGATGCCAATTTAGATAAAG<br>TTCTTAGTGCATATAACAAACATAGAGACAAACCAATACG<br>TGAACAAGCAGAAAATATTATTCATTTATTTACGTTGACG<br>AATCTTGGAGCTCCCGCTGCTTTTAAATATTTTGATACAA<br>CAATTGATCGTAAACGATATACGTCTACAAAAGAAGTTTT<br>AGATGCCACTCTTATCCATCAATCCATCACTGGTCTTTAT<br>GAAACACGCATTGATTTGAGTCAGCTAGGAGGTGACTGAG<br>GCATCAAATAAAACGAAAGGCTCAGTCGAAAGACTGGGCC<br>TTTCGTTTTATCTGTTGTTTGTCGGTGAACGCTCTCCTGA<br>GTAGGACAAATCCGCCGCCCTAGA | |
| *tetR* | transcription unit | GGTACCCGGGGATCCTCTAGAGTCGACCTGCAGGCATGCA<br>AGCTTTTTACGGCTAGCTCAGTCCTAGGTATAGTGCTAGC<br>CCAGCCAGAGAAACACTCTTTAACAGGGGGTAGTATGATG<br>TCTCGTTTAGATAAAAGTAAAGTGATTAACAGCGCATTAG<br>AGCTGCTTAATGAGGTCGGAATCGAAGGTTTAACAACCCG<br>TAAACTCGCCCAGAAGCTAGGTGTAGAGCAGCCTACATTG<br>TATTGGCATGTAAAAAATAAGCGGGCTTTGCTCGACGCCT<br>TAGCCATTGAGATGTTAGATAGGCACCATACTCACTTTTG<br>CCCTTTAGAAGGGGAAAGCTGGCAAGATTTTTTACGTAAT<br>AACGCTAAAAGTTTTAGATGTGCTTTACTAAGTCATCGCG<br>ATGGAGCAAAAGTACATTTAGGTACACGGCCTACAGAAAA<br>ACAGTATGAAACTCTCGAAAATCAATTAGCCTTTTTATGC<br>CAACAAGGTTTTTCACTAGAGAATGCATTATATGCACTCA<br>GCGCAGTGGGGCATTTTACTTTAGGTTGCGTATTGGAAGA<br>TCAAGAGCATCAAGTCGCTAAAGAAGAAAGGGAAACACCT<br>ACTACTGATAGTATGCCGCCATTATTACGACAAGCTATCG<br>AATTATTTGATCACCAAGGTGCAGAGCCAGCCTTCTTATT<br>CGGCCTTGAATTGATCATATGCGGATTAGAAAAACAACTT<br>AAATGTGAAAGTGGGTCTTAAGGCATCAAATAAAACGAAA<br>GGCTCAGTCGAAAGACTGGGCCTTTCGTTTTATCTGTTGT<br>TTGTCGGTGAACGCTCTCCTGAGTAGGACAAATCCGCCGC<br>CCTAGA | (Ramakrishnan & Tabor, 2016) |
| *lacI* | transcription unit | CGAAGCGGCATGCATTTACGTTGACACCATCGAATGGTGC<br>AAAACCTTTCGCGGTATGGCATGATAGCGCCCGGAAGAGA<br>GTCAATTCAGGGTGGTGAATGTGAAACCAGTAACGTTATA<br>CGATGTCGCAGAGTATGCCGGTGTCTCTTATCAGACCGTT<br>TCCCGCGTGGTGAACCAGGCCAGCCACGTTTCTGCGAAAA<br>CGCGGGAAAAAGTGGAAGCGGCGATGGCGGAGCTGAATTA<br>CATTCCCAACCGCGTGGCACAACAACTGGCGGGCAAACAG<br>TCGTTGCTGATTGGCGTTGCCACCTCCAGTCTGGCCCTGC<br>ACGCGCCGTCGCAAATTGTCGCGGCGATTAAATCTCGCGC<br>CGATCAACTGGGTGCCAGCGTGGTGGTGTCGATGGTAGAA<br>CGAAGCGGCGTCGAAGCCTGTAAAGCGGCGGTGCACAATC<br>TTCTCGCGCAACGCGTCAGTGGGCTGATCATTAACTATCC<br>GCTGGATGACCAGGATGCCATTGCTGTGGAAGCTGCCTGC<br>ACTAATGTTCCGGCGTTATTTCTTGATGTCTCTGACCAGA<br>CACCCATCAACAGTATTATTTTCTCCCATGAAGACGGTAC | Modified from (Ramakrishnan & Tabor, 2016) |

| | | | |
|---|---|---|---|
| | | GCGACTGGGCGTGGAGCATCTGGTCGCATTGGGTCACCAG<br>CAAATCGCGCTGTTAGCGGGCCCATTAAGTTCTGTCTCGG<br>CGCGTCTGCGTCTGGCTGGCTGGCATAAATATCTCACTCG<br>CAATCAAATTCAGCCGATAGCGGAACGGGAAGGCGACTGG<br>AGTGCCATGTCCGGTTTTCAACAAACCATGCAAATGCTGA<br>ATGAGGGCATCGTTCCCACTGCGATGCTGGTTGCCAACGA<br>TCAGATGGCGCTGGGCGCAATGCGCGCCATTACCGAGTCC<br>GGGCTGCGCGTTGGTGCGGATATCTCGGTAGTGGGATACG<br>ACGATACCGAAGACAGCTCATGTTATATCCCGCCGTTAAC<br>CACCATCAAACAGGATTTTCGCCTGCTGGGGCAAACCAGC<br>GTGGACCGCTTGCTGCAACTCTCTCAGGGCCAGGCGGTGA<br>AGGGCAATCAGCTGTTGCCCGTCTCACTGGTGAAAAGAAA<br>AACCACCCTGGCGCCCAATACGCAAACCGCCTCTCCCCGC<br>GCGTTGGCCGATTCATTAATGCAGCTGGCACGACAGGTTT<br>CCCGACTGGAAAGCGGGCAGTGACTCGGTACCAAATTCCA<br>GAAAAGACACCCGAAAGGGTGTTTTTTCGTTTTGGTCC | |
| *phlF* (DAPG sensor) | transcription unit | TGTAGAGTTATCCGCCTACGGCGCCGTCGTATCGGTAATC<br>CGTACGGGAATCGAAACGACGTCTACGAGCCTGACAGCTA<br>GCTCAGTCCTAGGTATAATGCTAGCCTGAAGTACGTCTGA<br>GCGTGATACCCGCTCACTGAAGATGGCCCGGTAGGGCCGA<br>AACGTACCTCTACAAATAATTTTGTTTAACTATGGACTAT<br>GTTTGAAAGGGAGAAATACTAGATGGCACGTACCCCGAGC<br>CGTAGCAGCATTGGTAGCCTGCGTAGTCCGCATACCCATA<br>AAGCAATTCTGACCAGCACCATTGAAATCCTGAAAGAATG<br>TGGTTATAGCGGTCTGAGCATTGAAAGCGTTGCACGTCGT<br>GCCGGTGCAAGCAAACCGACCATTTATCGTTGGTGGACCA<br>ATAAAGCAGCACTGATTGCCGAAGTGTATGAAAATGAAAG<br>CGAACAGGTGCGTAAATTTCCGGATCTGGGTAGCTTTAAA<br>GCCGATCTGGATTTTCTGCTGCGTAATCTGTGGAAAGTTT<br>GGCGTGAAACCATTTGTGGTGAAGCATTTCGTTGTGTTAT<br>TGCAGAAGCACAGCTGGACCCTGCAACCCTGACCCAGCTG<br>AAAGATCAGTTTATGGAACGTCGTCGTGAGATGCCGAAAA<br>AACTGGTTGAAAATGCCATTAGCAATGGTGAACTGCCGAA<br>AGATACCAATCGTGAACTGCTGCTGGATATGATTTTTGGT<br>TTTTGTTGGTATCGCCTGCTGACCGAACAGCTGACCGTTG<br>AACAGGATATTGAAGAATTTACCTTCCTGCTGATTAATGG<br>TGTTTGTCCGGGTACACAGCGTTAATAAGGTTGAAAAATA<br>AAAACGGCGCTAAAAAGCGCCGTTTTTTTTGACGGTGGTA | Modified from (Nielsen & Voigt, 2014) |
| *phlF* (DAPG sensor 2) | transcription unit | TGTAGAGTTATCCGCCTACGGCGCCGTCGTATCGGTAATC<br>CGTACGGGAATCGAAACGACGTCTACGAGCTTTATGGCTA<br>GCTCAGTCCTAGGTACAATGCTAGCCTGAAGTACGTCTGA<br>GCGTGATACCCGCTCACTGAAGATGGCCCGGTAGGGCCGA<br>AACGTACCTCTACAAATAATTTTGTTTAACTATGGACTAT<br>GTTTGAAAGGGAGAAATACTAGATGGCACGTACCCCGAGC<br>CGTAGCAGCATTGGTAGCCTGCGTAGTCCGCATACCCATA<br>AAGCAATTCTGACCAGCACCATTGAAATCCTGAAAGAATG<br>TGGTTATAGCGGTCTGAGCATTGAAAGCGTTGCACGTCGT<br>GCCGGTGCAAGCAAACCGACCATTTATCGTTGGTGGACCA<br>ATAAAGCAGCACTGATTGCCGAAGTGTATGAAAATGAAAG<br>CGAACAGGTGCGTAAATTTCCGGATCTGGGTAGCTTTAAA<br>GCCGATCTGGATTTTCTGCTGCGTAATCTGTGGAAAGTTT<br>GGCGTGAAACCATTTGTGGTGAAGCATTTCGTTGTGTTAT<br>TGCAGAAGCACAGCTGGACCCTGCAACCCTGACCCAGCTG<br>AAAGATCAGTTTATGGAACGTCGTCGTGAGATGCCGAAAA<br>AACTGGTTGAAAATGCCATTAGCAATGGTGAACTGCCGAA<br>AGATACCAATCGTGAACTGCTGCTGGATATGATTTTTGGT<br>TTTTGTTGGTATCGCCTGCTGACCGAACAGCTGACCGTTG | Modified from (Nielsen & Voigt, 2014) |

| | | | |
|---|---|---|---|
| | | AACAGGATATTGAAGAATTTACCTTCCTGCTGATTAATGG<br>TGTTTGTCCGGGTACACAGCGTTAATAAGGTTGAAAAATA<br>AAAACGGCGCTAAAAAGCGCCGTTTTTTTTGACGGTGGTA | |
| *luxI* | transcription unit | GTTACCTTCCCGGAGGTAGCCGCGTTCCGCCCGAGTCGGA<br>CGAGAACCGGGAGTCTTCGAAGGCTCTATCGACAATGTTG<br>TGTTACGTTGGTATAATAGATTCATCGCTGATAGTGCTAG<br>TGTAGATCGCTACTAGAGTCACACAGGAAACCTACTAGAT<br>GACTATAATGATAAAAAAATCGGATTTTTTGGCAATTCCA<br>TCGGAGGAGTATAAAGGTATTCTAAGTCTTCGTTATCAAG<br>TGTTTAAGCAAAGACTTGAGTGGGACTTAGTTGTAGAAAA<br>TAACCTTGAATCAGATGAGTATGATAACTCAAATGCAGAA<br>TATATTTATGCTTGTGATGATACTGAAAATGTAAGTGGAT<br>GCTGGCGTTTATTACCTACAACAGGTGATTATATGCTGAA<br>AAGTGTTTTTCCTGAATTGCTTGGTCAACAGAGTGCTCCC<br>AAAGATCCTAATATAGTCGAATTAAGTCGTTTTGCTGTAG<br>GTAAAAATAGCTCAAAGATAAATAACTCTGCTAGTGAAAT<br>TACAATGAAACTATTTGAAGCTATATATAAACACGCTGTT<br>AGTCAAGGTATTACAGAATATGTAACAGTAACATCAACAG<br>CAATAGAGCGATTTTTAAAGCGTATTAAAGTTCCTTGTCA<br>TCGTATTGGAGACAAAGAAATTCATGTATTAGGTGACACT<br>AAATCGGTTGTATTGTCTATGCCTATTAATGAACAGTTTA<br>AAAAAGCAGTCTTAAATGCTGCAAACGACGAAAACTACGC<br>TTTAGTAGCTTAATAAAACGCATGAGAAAGCCCCCGGAAG<br>ATCACCTTCCGGGGGCTTTTTTATTGCGC | Modified from (Tabor *et al*, 2009) |
| *luxR* | transcription unit | TGCCCGCTCGCGAGCGCGTCCTCTATAGATTCCTCGAGGA<br>GCGGATACTTCGTAGGGTAGACTCGGGTCCTTTATAGCTA<br>GCTCAGCCCTTGGTACAATGCTAGCTACTAGAGAAAGAGG<br>AGAAATACTAGATGAAAAACATAAATGCCGACGACACATA<br>CAGAATAATTAATAAAATTAAAGCTTGTAGAAGCAATAAT<br>GATATTAATCAATGCTTATCTGATATGACTAAAATGGTAC<br>ATTGTGAATATTATTTACTCGCGATCATTTATCCTCATTC<br>TATGGTTAAATCTGATATTTCAATCCTAGATAATTACCCT<br>AAAAAATGGAGGCAATATTATGATGACGCTAATTTAATAA<br>AATATGATCCTATAGTAGATTATTCTAACTCCAATCATTC<br>ACCAATTAATTGGAATATATTTGAAAACAATGCTGTAAAT<br>AAAAAATCTCCAAATGTAATTAAAGAAGCGAAAACATCAG<br>GTCTTATCACTGGGTTTAGTTTCCCTATTCATACGGCTAA<br>CAATGGCTTCGGAATGCTTAGTTTTGCACATTCAGAAAAA<br>GACAACTATATAGATAGTTTATTTTTACATGCGTGTATGA<br>ACATACCATTAATTGTTCCTTCTCTAGTTGATAATTATCG<br>AAAAATAAATATAGCAAATAATAAATCAAACAACGATTTA<br>ACCAAAAGAGAAAAAGAATGTTTAGCGTGGGCATGCGAAG<br>GAAAAAGCTCTTGGGATATTTCAAAAATATTAGGTTGCAG<br>TGAGCGTACTGTCACTTTCCATTTAACCAATGCGCAAATG<br>AAACTCAATACAACAAACCGCTGCCAAAGTATTTCTAAAG<br>CAATTTTAACAGGAGCAATTGATTGCCCATACTTTAAAAA<br>TTAATAAAACGCATGAGAAAGCCCCCGGAAGATCACCTTC<br>CGGGGGCTTTTTTATTGCGC | Modified from (Tabor *et al*, 2009) |
| *sfgfp* | insulated gene | AGCTGTCACCGGATGTGCTTTCCGGTCTGATGAGTCCGTG<br>AGGACGAAACAGCCTCTACAAATAATTTTGTTTAATAAGT<br>ATCCTCTAACCCTAAAGGGGCACAAAATCATGCGTAAAGG<br>CGAAGAGCTGTTCACTGGTGTCGTCCCTATTCTGGTGGAA<br>CTGGATGGTGATGTCAACGGTCATAAGTTTTCCGTGCGTG<br>GCGAGGGTGAAGGTGACGCAACTAATGGTAAACTGACGCT<br>GAAGTTCATCTGTACTACTGGTAAACTGCCGGTACCTTGG<br>CCGACTCTGGTAACGACGCTGACTTATGGTGTTCAGTGCT<br>TTGCTCGTTATCCGGACCATATGAAGCAGCATGACTTCTT | (Pédelacq *et al*, 2006; Lou *et al*, 2012) |

| | |
|---|---|
| | CAAGTCCGCCATGCCGGAAGGCTATGTGCAGGAACGCACG<br>ATTTCCTTTAAGGATGACGGCACGTACAAAACGCGTGCGG<br>AAGTGAAATTTGAAGGCGATACCCTGGTAAACCGCATTGA<br>GCTGAAAGGCATTGACTTTAAAGAAGACGGCAATATCCTG<br>GGCCATAAGCTGGAATACAATTTTAACAGCCACAATGTTT<br>ACATCACCGCCGATAAACAAAAAAATGGCATTAAAGCGAA<br>TTTTAAAATTCGCCACAACGTGGAGGATGGCAGCGTGCAG<br>CTGGCTGATCACTACCAGCAAAACACTCCAATCGGTGATG<br>GTCCTGTTCTGCTGCCAGACAATCACTATCTGAGCACGCA<br>AAGCGTTCTGTCTAAAGATCCGAACGAGAAACGCGATCAT<br>ATGGTTCTGCTGGAGTTCGTAACCGCAGCGGGCATCACGC<br>ATGGTATGGATGAACTGTACAAATGATGACCAGGCATCAA<br>ATAAAACGAAAGGCTCAGTCGAAAGACTGGGCCTTTCGTT<br>TTATCTGTTGTTTGTCGGTGAACGCTCTCTACTAGAGTCA<br>CACTGGCTCACCTTCGGGTGGGCCTTTCTGCGTTTATA |

**Appendix Table S5. Plasmids used in this study.** Annotated plasmid sequences are available online through the Benchling platform (https://www.benchling.com/) and as GenBank files in **Dataset EV2**. CmR = chloramphenicol, SpecR = spectinomycin, AmpR = ampicillin. Superscript u ($^u$) denotes uninsulated promoters.

| Label | Type | Description | Origin of replication | Resistance cassette |
|---|---|---|---|---|
| pJS0115 | circuit | $P_{LtetO\text{-}1}{}^u$:S1 | ColE1 | CmR |
| pJS0120 | circuit | $P_{LtetO\text{-}1}{}^u$:S2 | ColE1 | CmR |
| pJS0114 | circuit | $P_{LtetO\text{-}1}{}^u$:S3 | ColE1 | CmR |
| pJS0118 | circuit | $P_{LtetO\text{-}1}{}^u$:S4 | ColE1 | CmR |
| pJS0119 | circuit | $P_{LtetO\text{-}1}{}^u$:S5 | ColE1 | CmR |
| pJS0107 | circuit | $P_{LtetO\text{-}1}{}^u$:S6 | ColE1 | CmR |
| pJS0116 | circuit | $P_{LtetO\text{-}1}{}^u$:S7 | ColE1 | CmR |
| pJS0113 | circuit | $P_{LtetO\text{-}1}{}^u$:S8 | ColE1 | CmR |
| pJS0117 | circuit | $P_{LtetO\text{-}1}{}^u$:S9 | ColE1 | CmR |
| | | | | |
| pJS0101 | probe | $P1^u$:*sfgfp* | p15A | SpecR |
| pJS0106 | probe | $P2^u$:*sfgfp* | p15A | SpecR |
| pJS0100 | probe | $P3^u$:*sfgfp* | p15A | SpecR |
| pJS0104 | probe | $P4^u$:*sfgfp* | p15A | SpecR |
| pJS0105 | probe | $P5^u$:*sfgfp* | p15A | SpecR |
| pJS0087 | probe | $P6^u$:*sfgfp* | p15A | SpecR |
| pJS0102 | probe | $P7^u$:*sfgfp* | p15A | SpecR |
| pJS0099 | probe | $P8^u$:*sfgfp* | p15A | SpecR |
| pJS0103 | probe | $P9^u$:*sfgfp* | p15A | SpecR |
| | | | | |
| pSC31_1 | dCas9 | Weak constitutive dCas9 expression | pSC101* | AmpR |
| | | | | |
| pJS0344 | circuit | aTc-NOT1 | ColE1 | CmR |
| pJS0349 | circuit | aTc-NOT2 | ColE1 | CmR |
| pJS0343 | circuit | aTc-NOT3 | ColE1 | CmR |
| pJS0347 | circuit | aTc-NOT4 | ColE1 | CmR |
| pJS0348 | circuit | aTc-NOT5 | ColE1 | CmR |
| pJS0341 | circuit | aTc-NOT6 | ColE1 | CmR |
| pJS0345 | circuit | aTc-NOT7 | ColE1 | CmR |
| pJS0342 | circuit | aTc-NOT8 | ColE1 | CmR |
| pJS0346 | circuit | aTc-NOT9 | ColE1 | CmR |
| | | | | |
| pJS0275 | probe | $P_{tet}$:*sfgfp* | p15A | SpecR |
| pJS0307 | probe | P1:*sfgfp* | p15A | SpecR |
| pJS0312 | probe | P2:*sfgfp* | p15A | SpecR |
| pJS0306 | probe | P3:*sfgfp* | p15A | SpecR |
| pJS0310 | probe | P4:*sfgfp* | p15A | SpecR |
| pJS0311 | probe | P5:*sfgfp* | p15A | SpecR |
| pJS0337 | probe | P6:*sfgfp* | p15A | SpecR |
| pJS0308 | probe | P7:*sfgfp* | p15A | SpecR |
| pJS0305 | probe | P8:*sfgfp* | p15A | SpecR |
| pJS0309 | probe | P9:*sfgfp* | p15A | SpecR |
| | | | | |
| pSC31_3 | dCas9 | Strong constitutive dCas9 expression | pSC101* | AmpR |
| | | | | |
| pJS0143 | circuit | Empty circuit plasmid | ColE1 | CmR |
| pJS0130 | probe | Empty probe plasmid | p15A | SpecR |

| | | | | |
|---|---|---|---|---|
| pJS0122 | circuit | MUX (P1=0, P9=0, P4=0) | ColE1 | CmR |
| pJS0123 | circuit | MUX (P1=0, P9=1, P4=0) | ColE1 | CmR |
| pJS0156 | circuit | MUX (P1=1, P9=0, P4=0) | ColE1 | CmR |
| pJS0157 | circuit | MUX (P1=1, P9=1, P4=0) | ColE1 | CmR |
| pJS0126 | circuit | MUX (P1=0, P9=0, P4=1) | ColE1 | CmR |
| pJS0127 | circuit | MUX (P1=0, P9=1, P4=1) | ColE1 | CmR |
| pJS0158 | circuit | MUX (P1=1, P9=0, P4=1) | ColE1 | CmR |
| pJS0155 | circuit | MUX (P1=1, P9=1, P4=1) | ColE1 | CmR |
| | | | | |
| pJS0162 | circuit | DEMUX ($P_R$=0, P3=0) | ColE1 | CmR |
| pJS0133 | circuit | DEMUX ($P_R$=1, P3=0) | ColE1 | CmR |
| pJS0164 | circuit | DEMUX ($P_R$=0, P3=1) | ColE1 | CmR |
| pJS0134 | circuit | DEMUX ($P_R$=1, P3=1) | ColE1 | CmR |
| pJS0002 | probe | $P_R$:*sfgfp* | p15A | SpecR |
| | | | | |
| pJS0356 | circuit | SENSOR-MUX-AHL | ColE1 | CmR |
| | | | | |
| pJS0338 | circuit | aTc sensor | ColE1 | CmR |
| pJS0339 | circuit | IPTG sensor | ColE1 | CmR |
| pJS0355 | circuit | DAPG sensor | ColE1 | CmR |
| pJS0340 | circuit | DAPG sensor 2 | ColE1 | CmR |
| pJS0260 | probe | $P_{tac}$:*sfgfp* | p15A | SpecR |
| pJS0304 | probe | $P_{PhlF}$:*sfgfp* | p15A | SpecR |
| | | | | |
| pJS0318 | circuit | $SENDER_{BCD22}$ | ColE1 | CmR |
| pJS0317 | circuit | $SENDER_{B0031}$ | ColE1 | CmR |
| pJS0315 | circuit | $SENDER_{B0030}$ | ColE1 | CmR |
| pJS0286 | circuit | $SENDER_{B0034}$ | ColE1 | CmR |
| pJS0285 | circuit | $RECEIVER_{J23117}$ | ColE1 | CmR |
| pJS0284 | circuit | $RECEIVER_{J23115(*)}$ | ColE1 | CmR |
| pJS0283 | circuit | $RECEIVER_{J23105}$ | ColE1 | CmR |
| pJS0282 | circuit | $RECEIVER_{J23107}$ | ColE1 | CmR |
| pJS0205 | marker | P2:*mCherry* | p15A | SpecR |
| pJS0281 | probe | $P_{lux}$:*sfgfp* | p15A | SpecR |
| | | | | |
| pJS0277 | circuit | SENSOR-MUX | ColE1 | CmR |
| | | | | |
| pJS0350 | circuit | aTc-NOT6* | ColE1 | CmR |
| pJS0200 | probe | P6*:*sfgfp* | p15A | SpecR |
| | | | | |
| pJS0333 | circuit | AHL-DEMUX | ColE1 | CmR |
| | | | | |
| pJS0357 | circuit | AHL-DEMUX v0.1 | ColE1 | CmR |
| pJS0314 | circuit | AHL-DEMUX v0.2 | ColE1 | CmR |
| pJS0326 | probe | $P_{lux*}$:*sfgfp* | p15A | SpecR |

**Appendix Table S6. Bacterial strains used in this study.** All strains were derived from *E. coli* K-12 MG1655. Superscript u ($^{u}$) denotes uninsulated promoters.

| Label | DNA content | Description | Figure(s) |
|---|---|---|---|
| sJS0329 | pJS0115, pJS0101, pSC31_1 | $P_{LtetO\text{-}1}^{u}$:S1, $P1^{u}$:*sfgfp* | 2C |
| sJS0334 | pJS0120, pJS0101, pSC31_1 | $P_{LtetO\text{-}1}^{u}$:S2, $P1^{u}$:*sfgfp* | 2C |
| sJS0328 | pJS0114, pJS0101, pSC31_1 | $P_{LtetO\text{-}1}^{u}$:S3, $P1^{u}$:*sfgfp* | 2C |
| sJS0332 | pJS0118, pJS0101, pSC31_1 | $P_{LtetO\text{-}1}^{u}$:S4, $P1^{u}$:*sfgfp* | 2C |
| sJS0333 | pJS0119, pJS0101, pSC31_1 | $P_{LtetO\text{-}1}^{u}$:S5, $P1^{u}$:*sfgfp* | 2C |
| sJS0321 | pJS0107, pJS0101, pSC31_1 | $P_{LtetO\text{-}1}^{u}$:S6, $P1^{u}$:*sfgfp* | 2C |
| sJS0330 | pJS0116, pJS0101, pSC31_1 | $P_{LtetO\text{-}1}^{u}$:S7, $P1^{u}$:*sfgfp* | 2C |
| sJS0327 | pJS0113, pJS0101, pSC31_1 | $P_{LtetO\text{-}1}^{u}$:S8, $P1^{u}$:*sfgfp* | 2C |
| sJS0331 | pJS0117, pJS0101, pSC31_1 | $P_{LtetO\text{-}1}^{u}$:S9, $P1^{u}$:*sfgfp* | 2C |
| sJS0399 | pJS0115, pJS0106, pSC31_1 | $P_{LtetO\text{-}1}^{u}$:S1, $P2^{u}$:*sfgfp* | 2C |
| sJS0404 | pJS0120, pJS0106, pSC31_1 | $P_{LtetO\text{-}1}^{u}$:S2, $P2^{u}$:*sfgfp* | 2C |
| sJS0398 | pJS0114, pJS0106, pSC31_1 | $P_{LtetO\text{-}1}^{u}$:S3, $P2^{u}$:*sfgfp* | 2C |
| sJS0402 | pJS0118, pJS0106, pSC31_1 | $P_{LtetO\text{-}1}^{u}$:S4, $P2^{u}$:*sfgfp* | 2C |
| sJS0403 | pJS0119, pJS0106, pSC31_1 | $P_{LtetO\text{-}1}^{u}$:S5, $P2^{u}$:*sfgfp* | 2C |
| sJS0391 | pJS0107, pJS0106, pSC31_1 | $P_{LtetO\text{-}1}^{u}$:S6, $P2^{u}$:*sfgfp* | 2C |
| sJS0400 | pJS0116, pJS0106, pSC31_1 | $P_{LtetO\text{-}1}^{u}$:S7, $P2^{u}$:*sfgfp* | 2C |
| sJS0397 | pJS0113, pJS0106, pSC31_1 | $P_{LtetO\text{-}1}^{u}$:S8, $P2^{u}$:*sfgfp* | 2C |
| sJS0401 | pJS0117, pJS0106, pSC31_1 | $P_{LtetO\text{-}1}^{u}$:S9, $P2^{u}$:*sfgfp* | 2C |
| sJS0315 | pJS0115, pJS0100, pSC31_1 | $P_{LtetO\text{-}1}^{u}$:S1, $P3^{u}$:*sfgfp* | 2C |
| sJS0320 | pJS0120, pJS0100, pSC31_1 | $P_{LtetO\text{-}1}^{u}$:S2, $P3^{u}$:*sfgfp* | 2C |
| sJS0314 | pJS0114, pJS0100, pSC31_1 | $P_{LtetO\text{-}1}^{u}$:S3, $P3^{u}$:*sfgfp* | 2C |
| sJS0318 | pJS0118, pJS0100, pSC31_1 | $P_{LtetO\text{-}1}^{u}$:S4, $P3^{u}$:*sfgfp* | 2C |
| sJS0319 | pJS0119, pJS0100, pSC31_1 | $P_{LtetO\text{-}1}^{u}$:S5, $P3^{u}$:*sfgfp* | 2C |
| sJS0307 | pJS0107, pJS0100, pSC31_1 | $P_{LtetO\text{-}1}^{u}$:S6, $P3^{u}$:*sfgfp* | 2C |
| sJS0316 | pJS0116, pJS0100, pSC31_1 | $P_{LtetO\text{-}1}^{u}$:S7, $P3^{u}$:*sfgfp* | 2C |
| sJS0313 | pJS0113, pJS0100, pSC31_1 | $P_{LtetO\text{-}1}^{u}$:S8, $P3^{u}$:*sfgfp* | 2C |
| sJS0317 | pJS0117, pJS0100, pSC31_1 | $P_{LtetO\text{-}1}^{u}$:S9, $P3^{u}$:*sfgfp* | 2C |
| sJS0371 | pJS0115, pJS0104, pSC31_1 | $P_{LtetO\text{-}1}^{u}$:S1, $P4^{u}$:*sfgfp* | 2C |
| sJS0376 | pJS0120, pJS0104, pSC31_1 | $P_{LtetO\text{-}1}^{u}$:S2, $P4^{u}$:*sfgfp* | 2C |
| sJS0370 | pJS0114, pJS0104, pSC31_1 | $P_{LtetO\text{-}1}^{u}$:S3, $P4^{u}$:*sfgfp* | 2C |
| sJS0374 | pJS0118, pJS0104, pSC31_1 | $P_{LtetO\text{-}1}^{u}$:S4, $P4^{u}$:*sfgfp* | 2C |
| sJS0375 | pJS0119, pJS0104, pSC31_1 | $P_{LtetO\text{-}1}^{u}$:S5, $P4^{u}$:*sfgfp* | 2C |
| sJS0363 | pJS0107, pJS0104, pSC31_1 | $P_{LtetO\text{-}1}^{u}$:S6, $P4^{u}$:*sfgfp* | 2C |
| sJS0372 | pJS0116, pJS0104, pSC31_1 | $P_{LtetO\text{-}1}^{u}$:S7, $P4^{u}$:*sfgfp* | 2C |
| sJS0369 | pJS0113, pJS0104, pSC31_1 | $P_{LtetO\text{-}1}^{u}$:S8, $P4^{u}$:*sfgfp* | 2C |
| sJS0373 | pJS0117, pJS0104, pSC31_1 | $P_{LtetO\text{-}1}^{u}$:S9, $P4^{u}$:*sfgfp* | 2C |
| sJS0385 | pJS0115, pJS0105, pSC31_1 | $P_{LtetO\text{-}1}^{u}$:S1, $P5^{u}$:*sfgfp* | 2C |
| sJS0390 | pJS0120, pJS0105, pSC31_1 | $P_{LtetO\text{-}1}^{u}$:S2, $P5^{u}$:*sfgfp* | 2C |
| sJS0384 | pJS0114, pJS0105, pSC31_1 | $P_{LtetO\text{-}1}^{u}$:S3, $P5^{u}$:*sfgfp* | 2C |
| sJS0388 | pJS0118, pJS0105, pSC31_1 | $P_{LtetO\text{-}1}^{u}$:S4, $P5^{u}$:*sfgfp* | 2C |
| sJS0389 | pJS0119, pJS0105, pSC31_1 | $P_{LtetO\text{-}1}^{u}$:S5, $P5^{u}$:*sfgfp* | 2C |
| sJS0377 | pJS0107, pJS0105, pSC31_1 | $P_{LtetO\text{-}1}^{u}$:S6, $P5^{u}$:*sfgfp* | 2C |
| sJS0386 | pJS0116, pJS0105, pSC31_1 | $P_{LtetO\text{-}1}^{u}$:S7, $P5^{u}$:*sfgfp* | 2C |
| sJS0383 | pJS0113, pJS0105, pSC31_1 | $P_{LtetO\text{-}1}^{u}$:S8, $P5^{u}$:*sfgfp* | 2C |
| sJS0387 | pJS0117, pJS0105, pSC31_1 | $P_{LtetO\text{-}1}^{u}$:S9, $P5^{u}$:*sfgfp* | 2C |
| sJS0217 | pJS0115, pJS0087, pSC31_1 | $P_{LtetO\text{-}1}^{u}$:S1, $P6^{u}$:*sfgfp* | 2C |
| sJS0222 | pJS0120, pJS0087, pSC31_1 | $P_{LtetO\text{-}1}^{u}$:S2, $P6^{u}$:*sfgfp* | 2C |
| sJS0216 | pJS0114, pJS0087, pSC31_1 | $P_{LtetO\text{-}1}^{u}$:S3, $P6^{u}$:*sfgfp* | 2C |
| sJS0220 | pJS0118, pJS0087, pSC31_1 | $P_{LtetO\text{-}1}^{u}$:S4, $P6^{u}$:*sfgfp* | 2C |
| sJS0221 | pJS0119, pJS0087, pSC31_1 | $P_{LtetO\text{-}1}^{u}$:S5, $P6^{u}$:*sfgfp* | 2C |
| sJS0209 | pJS0107, pJS0087, pSC31_1 | $P_{LtetO\text{-}1}^{u}$:S6, $P6^{u}$:*sfgfp* | 2C |

| | | | |
|---|---|---|---|
| sJS0218 | pJS0116, pJS0087, pSC31_1 | $P_{LtetO-1}^{u}$:S7, $P6^{u}$:*sfgfp* | 2C |
| sJS0215 | pJS0113, pJS0087, pSC31_1 | $P_{LtetO-1}^{u}$:S8, $P6^{u}$:*sfgfp* | 2C |
| sJS0219 | pJS0117, pJS0087, pSC31_1 | $P_{LtetO-1}^{u}$:S9, $P6^{u}$:*sfgfp* | 2C |
| sJS0343 | pJS0115, pJS0102, pSC31_1 | $P_{LtetO-1}^{u}$:S1, $P7^{u}$:*sfgfp* | 2C |
| sJS0348 | pJS0120, pJS0102, pSC31_1 | $P_{LtetO-1}^{u}$:S2, $P7^{u}$:*sfgfp* | 2C |
| sJS0342 | pJS0114, pJS0102, pSC31_1 | $P_{LtetO-1}^{u}$:S3, $P7^{u}$:*sfgfp* | 2C |
| sJS0346 | pJS0118, pJS0102, pSC31_1 | $P_{LtetO-1}^{u}$:S4, $P7^{u}$:*sfgfp* | 2C |
| sJS0347 | pJS0119, pJS0102, pSC31_1 | $P_{LtetO-1}^{u}$:S5, $P7^{u}$:*sfgfp* | 2C |
| sJS0335 | pJS0107, pJS0102, pSC31_1 | $P_{LtetO-1}^{u}$:S6, $P7^{u}$:*sfgfp* | 2C |
| sJS0344 | pJS0116, pJS0102, pSC31_1 | $P_{LtetO-1}^{u}$:S7, $P7^{u}$:*sfgfp* | 2C |
| sJS0341 | pJS0113, pJS0102, pSC31_1 | $P_{LtetO-1}^{u}$:S8, $P7^{u}$:*sfgfp* | 2C |
| sJS0345 | pJS0117, pJS0102, pSC31_1 | $P_{LtetO-1}^{u}$:S9, $P7^{u}$:*sfgfp* | 2C |
| sJS0301 | pJS0115, pJS0099, pSC31_1 | $P_{LtetO-1}^{u}$:S1, $P8^{u}$:*sfgfp* | 2C |
| sJS0306 | pJS0120, pJS0099, pSC31_1 | $P_{LtetO-1}^{u}$:S2, $P8^{u}$:*sfgfp* | 2C |
| sJS0300 | pJS0114, pJS0099, pSC31_1 | $P_{LtetO-1}^{u}$:S3, $P8^{u}$:*sfgfp* | 2C |
| sJS0304 | pJS0118, pJS0099, pSC31_1 | $P_{LtetO-1}^{u}$:S4, $P8^{u}$:*sfgfp* | 2C |
| sJS0305 | pJS0119, pJS0099, pSC31_1 | $P_{LtetO-1}^{u}$:S5, $P8^{u}$:*sfgfp* | 2C |
| sJS0293 | pJS0107, pJS0099, pSC31_1 | $P_{LtetO-1}^{u}$:S6, $P8^{u}$:*sfgfp* | 2C |
| sJS0302 | pJS0116, pJS0099, pSC31_1 | $P_{LtetO-1}^{u}$:S7, $P8^{u}$:*sfgfp* | 2C |
| sJS0299 | pJS0113, pJS0099, pSC31_1 | $P_{LtetO-1}^{u}$:S8, $P8^{u}$:*sfgfp* | 2C |
| sJS0303 | pJS0117, pJS0099, pSC31_1 | $P_{LtetO-1}^{u}$:S9, $P8^{u}$:*sfgfp* | 2C |
| sJS0357 | pJS0115, pJS0103, pSC31_1 | $P_{LtetO-1}^{u}$:S1, $P9^{u}$:*sfgfp* | 2C |
| sJS0362 | pJS0120, pJS0103, pSC31_1 | $P_{LtetO-1}^{u}$:S2, $P9^{u}$:*sfgfp* | 2C |
| sJS0356 | pJS0114, pJS0103, pSC31_1 | $P_{LtetO-1}^{u}$:S3, $P9^{u}$:*sfgfp* | 2C |
| sJS0360 | pJS0118, pJS0103, pSC31_1 | $P_{LtetO-1}^{u}$:S4, $P9^{u}$:*sfgfp* | 2C |
| sJS0361 | pJS0119, pJS0103, pSC31_1 | $P_{LtetO-1}^{u}$:S5, $P9^{u}$:*sfgfp* | 2C |
| sJS0349 | pJS0107, pJS0103, pSC31_1 | $P_{LtetO-1}^{u}$:S6, $P9^{u}$:*sfgfp* | 2C |
| sJS0358 | pJS0116, pJS0103, pSC31_1 | $P_{LtetO-1}^{u}$:S7, $P9^{u}$:*sfgfp* | 2C |
| sJS0355 | pJS0113, pJS0103, pSC31_1 | $P_{LtetO-1}^{u}$:S8, $P9^{u}$:*sfgfp* | 2C |
| sJS0359 | pJS0117, pJS0103, pSC31_1 | $P_{LtetO-1}^{u}$:S9, $P9^{u}$:*sfgfp* | 2C |
| sJS0061 | pSC31_1 | Autofluorescence control | 2C |
| | | | |
| sJS1213 | pJS0344, pJS0275, pSC31_3 | aTc-NOT1, $P_{tet}$:*sfgfp* | 2E |
| sJS1106 | pJS0344, pJS0307, pSC31_3 | aTc-NOT1, P1:*sfgfp* | 2E |
| sJS1218 | pJS0349, pJS0275, pSC31_3 | aTc-NOT2, $P_{tet}$:*sfgfp* | 2E |
| sJS1111 | pJS0349, pJS0312, pSC31_3 | aTc-NOT2, P2:*sfgfp* | 2E |
| sJS1212 | pJS0343, pJS0275, pSC31_3 | aTc-NOT3, $P_{tet}$:*sfgfp* | 2E |
| sJS1105 | pJS0343, pJS0306, pSC31_3 | aTc-NOT3, P3:*sfgfp* | 2E |
| sJS1216 | pJS0347, pJS0275, pSC31_3 | aTc-NOT4, $P_{tet}$:*sfgfp* | 2E |
| sJS1109 | pJS0347, pJS0310, pSC31_3 | aTc-NOT4, P4:*sfgfp* | 2E |
| sJS1217 | pJS0348, pJS0275, pSC31_3 | aTc-NOT5, $P_{tet}$:*sfgfp* | 2E |
| sJS1110 | pJS0348, pJS0311, pSC31_3 | aTc-NOT5, P5:*sfgfp* | 2E |
| sJS1210 | pJS0341, pJS0275, pSC31_3 | aTc-NOT6, $P_{tet}$:*sfgfp* | 2E |
| sJS1103 | pJS0341, pJS0337, pSC31_3 | aTc-NOT6, P6:*sfgfp* | 2E |
| sJS1214 | pJS0345, pJS0275, pSC31_3 | aTc-NOT7, $P_{tet}$:*sfgfp* | 2E |
| sJS1107 | pJS0345, pJS0308, pSC31_3 | aTc-NOT7, P7:*sfgfp* | 2E |
| sJS1211 | pJS0342, pJS0275, pSC31_3 | aTc-NOT8, $P_{tet}$:*sfgfp* | 2E |
| sJS1104 | pJS0342, pJS0305, pSC31_3 | aTc-NOT8, P8:*sfgfp* | 2E |
| sJS1215 | pJS0346, pJS0275, pSC31_3 | aTc-NOT9, $P_{tet}$:*sfgfp* | 2E |
| sJS1108 | pJS0346, pJS0309, pSC31_3 | aTc-NOT9, P9:*sfgfp* | 2E |
| | | | |
| sJS1015 | pJS0143, pJS0307, pSC31_3 | P1:*sfgfp* | 2E, 4, 7, S7 |
| sJS1020 | pJS0143, pJS0312, pSC31_3 | P2:*sfgfp* | 2E, 3-7, S(7,9-11,13,15) |
| sJS1014 | pJS0143, pJS0306, pSC31_3 | P3:*sfgfp* | 2E, 3A, 4-6, S(7,9-11,13-15) |
| sJS1018 | pJS0143, pJS0310, pSC31_3 | P4:*sfgfp* | 2E, 4, S(7,15) |
| sJS1019 | pJS0143, pJS0311, pSC31_3 | P5:*sfgfp* | 2E, 3A, 4, S7 |

| | | | |
|---|---|---|---|
| sJS1092 | pJS0143, pJS0337, pSC31_3 | P6:*sfgfp* | 2E, 3A, S7 |
| sJS1016 | pJS0143, pJS0308, pSC31_3 | P7:*sfgfp* | 2E, 3B, 5-7, S(9-11,13,15) |
| sJS1013 | pJS0143, pJS0305, pSC31_3 | P8:*sfgfp* | 2E, 3B, 5, S(9-11,13,15) |
| sJS1017 | pJS0143, pJS0309, pSC31_3 | P9:*sfgfp* | 2E, 3B, 4, 5, 7, S(7,9-11,13) |
| sJS1007 | pJS0143, pJS0130, pSC31_3 | Autofluorescence control | 2E, 3-7, S(5-15) |
| sJS1132 | pJS0122, pJS0311, pSC31_3 | MUX (P1=0, P9=0, P4=0), P5:*sfgfp* | 3A |
| sJS1133 | pJS0123, pJS0311, pSC31_3 | MUX (P1=0, P9=1, P4=0), P5:*sfgfp* | 3A |
| sJS1137 | pJS0156, pJS0311, pSC31_3 | MUX (P1=1, P9=0, P4=0), P5:*sfgfp* | 3A |
| sJS1138 | pJS0157, pJS0311, pSC31_3 | MUX (P1=1, P9=1, P4=0), P5:*sfgfp* | 3A |
| sJS1134 | pJS0126, pJS0311, pSC31_3 | MUX (P1=0, P9=0, P4=1), P5:*sfgfp* | 3A |
| sJS1135 | pJS0127, pJS0311, pSC31_3 | MUX (P1=0, P9=1, P4=1), P5:*sfgfp* | 3A |
| sJS1139 | pJS0158, pJS0311, pSC31_3 | MUX (P1=1, P9=0, P4=1), P5:*sfgfp* | 3A |
| sJS1136 | pJS0155, pJS0311, pSC31_3 | MUX (P1=1, P9=1, P4=1), P5:*sfgfp* | 3A |
| sJS1124 | pJS0122, pJS0306, pSC31_3 | MUX (P1=0, P9=0, P4=0), P3:*sfgfp* | 3A |
| sJS1125 | pJS0123, pJS0306, pSC31_3 | MUX (P1=0, P9=1, P4=0), P3:*sfgfp* | 3A |
| sJS1129 | pJS0156, pJS0306, pSC31_3 | MUX (P1=1, P9=0, P4=0), P3:*sfgfp* | 3A |
| sJS1130 | pJS0157, pJS0306, pSC31_3 | MUX (P1=1, P9=1, P4=0), P3:*sfgfp* | 3A |
| sJS1126 | pJS0126, pJS0306, pSC31_3 | MUX (P1=0, P9=0, P4=1), P3:*sfgfp* | 3A |
| sJS1127 | pJS0127, pJS0306, pSC31_3 | MUX (P1=0, P9=1, P4=1), P3:*sfgfp* | 3A |
| sJS1131 | pJS0158, pJS0306, pSC31_3 | MUX (P1=1, P9=0, P4=1), P3:*sfgfp* | 3A |
| sJS1128 | pJS0155, pJS0306, pSC31_3 | MUX (P1=1, P9=1, P4=1), P3:*sfgfp* | 3A |
| sJS1148 | pJS0122, pJS0312, pSC31_3 | MUX (P1=0, P9=0, P4=0), P2:*sfgfp* | 3A |
| sJS1149 | pJS0123, pJS0312, pSC31_3 | MUX (P1=0, P9=1, P4=0), P2:*sfgfp* | 3A |
| sJS1153 | pJS0156, pJS0312, pSC31_3 | MUX (P1=1, P9=0, P4=0), P2:*sfgfp* | 3A |
| sJS1154 | pJS0157, pJS0312, pSC31_3 | MUX (P1=1, P9=1, P4=0), P2:*sfgfp* | 3A |
| sJS1150 | pJS0126, pJS0312, pSC31_3 | MUX (P1=0, P9=0, P4=1), P2:*sfgfp* | 3A |
| sJS1151 | pJS0127, pJS0312, pSC31_3 | MUX (P1=0, P9=1, P4=1), P2:*sfgfp* | 3A |
| sJS1155 | pJS0158, pJS0312, pSC31_3 | MUX (P1=1, P9=0, P4=1), P2:*sfgfp* | 3A |
| sJS1152 | pJS0155, pJS0312, pSC31_3 | MUX (P1=1, P9=1, P4=1), P2:*sfgfp* | 3A |
| sJS1140 | pJS0122, pJS0337, pSC31_3 | MUX (P1=0, P9=0, P4=0), P6:*sfgfp* | 3A |
| sJS1141 | pJS0123, pJS0337, pSC31_3 | MUX (P1=0, P9=1, P4=0), P6:*sfgfp* | 3A |
| sJS1145 | pJS0156, pJS0337, pSC31_3 | MUX (P1=1, P9=0, P4=0), P6:*sfgfp* | 3A |
| sJS1146 | pJS0157, pJS0337, pSC31_3 | MUX (P1=1, P9=1, P4=0), P6:*sfgfp* | 3A |
| sJS1142 | pJS0126, pJS0337, pSC31_3 | MUX (P1=0, P9=0, P4=1), P6:*sfgfp* | 3A |
| sJS1143 | pJS0127, pJS0337, pSC31_3 | MUX (P1=0, P9=1, P4=1), P6:*sfgfp* | 3A |
| sJS1147 | pJS0158, pJS0337, pSC31_3 | MUX (P1=1, P9=0, P4=1), P6:*sfgfp* | 3A |
| sJS1144 | pJS0155, pJS0337, pSC31_3 | MUX (P1=1, P9=1, P4=1), P6:*sfgfp* | 3A |
| sJS1161 | pJS0162, pJS0305, pSC31_3 | DEMUX ($P_R$=0, P3=0), P8:*sfgfp* | 3B |
| sJS1176 | pJS0133, pJS0305, pSC31_3 | DEMUX ($P_R$=1, P3=0), P8:*sfgfp* | 3B |
| sJS1163 | pJS0164, pJS0305, pSC31_3 | DEMUX ($P_R$=0, P3=1), P8:*sfgfp* | 3B |
| sJS1177 | pJS0134, pJS0305, pSC31_3 | DEMUX ($P_R$=1, P3=1), P8:*sfgfp* | 3B |
| sJS1169 | pJS0162, pJS0308, pSC31_3 | DEMUX ($P_R$=0, P3=0), P7:*sfgfp* | 3B |
| sJS1180 | pJS0133, pJS0308, pSC31_3 | DEMUX ($P_R$=1, P3=0), P7:*sfgfp* | 3B |
| sJS1171 | pJS0164, pJS0308, pSC31_3 | DEMUX ($P_R$=0, P3=1), P7:*sfgfp* | 3B |
| sJS1181 | pJS0134, pJS0308, pSC31_3 | DEMUX ($P_R$=1, P3=1), P7:*sfgfp* | 3B |
| sJS1165 | pJS0162, pJS0309, pSC31_3 | DEMUX ($P_R$=0, P3=0), P9:*sfgfp* | 3B |
| sJS1178 | pJS0133, pJS0309, pSC31_3 | DEMUX ($P_R$=1, P3=0), P9:*sfgfp* | 3B |
| sJS1167 | pJS0164, pJS0309, pSC31_3 | DEMUX ($P_R$=0, P3=1), P9:*sfgfp* | 3B |
| sJS1179 | pJS0134, pJS0309, pSC31_3 | DEMUX ($P_R$=1, P3=1), P9:*sfgfp* | 3B |
| sJS1173 | pJS0162, pJS0312, pSC31_3 | DEMUX ($P_R$=0, P3=0), P2:*sfgfp* | 3B |
| sJS1182 | pJS0133, pJS0312, pSC31_3 | DEMUX ($P_R$=1, P3=0), P2:*sfgfp* | 3B |
| sJS1175 | pJS0164, pJS0312, pSC31_3 | DEMUX ($P_R$=0, P3=1), P2:*sfgfp* | 3B |
| sJS1183 | pJS0134, pJS0312, pSC31_3 | DEMUX ($P_R$=1, P3=1), P2:*sfgfp* | 3B |

| | | | |
|---|---|---|---|
| sJS0595 | pJS0002, pSC31_3 | $P_{R}$:*sfgfp* | DEMUX model (3B) |
| | | | |
| sJS1232 | pJS0356, pJS0275, pSC31_3 | SENSOR-MUX-AHL, $P_{tet}$:*sfgfp* | 4 |
| sJS1233 | pJS0356, pJS0260, pSC31_3 | SENSOR-MUX-AHL, $P_{tac}$:*sfgfp* | 4 |
| sJS1234 | pJS0356, pJS0304, pSC31_3 | SENSOR-MUX-AHL, $P_{PhlF}$:*sfgfp* | 4, S15 |
| sJS1235 | pJS0356, pJS0307, pSC31_3 | SENSOR-MUX-AHL, P1:*sfgfp* | 4, 7 |
| sJS1238 | pJS0356, pJS0309, pSC31_3 | SENSOR-MUX-AHL, P9:*sfgfp* | 4, 7 |
| sJS1241 | pJS0356, pJS0310, pSC31_3 | SENSOR-MUX-AHL, P4:*sfgfp* | 4, S15 |
| sJS1236 | pJS0356, pJS0311, pSC31_3 | SENSOR-MUX-AHL, P5:*sfgfp* | 4 |
| sJS1237 | pJS0356, pJS0306, pSC31_3 | SENSOR-MUX-AHL, P3:*sfgfp* | 4, 6, S(14,15) |
| sJS1239 | pJS0356, pJS0312, pSC31_3 | SENSOR-MUX-AHL, P2:*sfgfp* | 4, S15 |
| sJS1240 | pJS0356, pJS0200, pSC31_3 | SENSOR-MUX-AHL, P6*:*sfgfp* | 4, S15 |
| | | | |
| sJS1094 | pJS0338, pJS0275, pSC31_3 | aTc sensor, $P_{tet}$:*sfgfp* | S5D |
| sJS1083 | pJS0339, pJS0260, pSC31_3 | IPTG sensor, $P_{tac}$:*sfgfp* | S5E |
| sJS1123 | pJS0355, pJS0304, pSC31_3 | DAPG sensor, $P_{PhlF}$:*sfgfp* | S5F |
| sJS1091 | pJS0340, pJS0304, pSC31_3 | DAPG sensor 2, $P_{PhlF}$:*sfgfp* | AHL-DEMUX model (5, S15) |
| sJS1009 | pJS0143, pJS0275, pSC31_3 | $P_{tet}$:*sfgfp* | 4, S(5D,7) |
| sJS1010 | pJS0143, pJS0260, pSC31_3 | $P_{tac}$:*sfgfp* | 4, S(5E,7) |
| sJS1012 | pJS0143, pJS0304, pSC31_3 | $P_{PhlF}$:*sfgfp* | 4, 5, S(5F,7,9-11,13,15) |
| | | | |
| sJS1051 | pJS0318, pJS0205, pSC31_3 | $SENDER_{BCD22}$, P2:*mCherry* | S6 |
| sJS1050 | pJS0317, pJS0205, pSC31_3 | $SENDER_{B0031}$, P2:*mCherry* | S6 |
| sJS1049 | pJS0315, pJS0205, pSC31_3 | $SENDER_{B0030}$, P2:*mCherry* | S6 |
| sJS0866 | pJS0286, pJS0205, pSC31_3 | $SENDER_{B0034}$, P2:*mCherry* | S6 |
| sJS0865 | pJS0285, pJS0281, pSC31_3 | $RECEIVER_{J23117}$, $P_{lux}$:*sfgfp* | S6 |
| sJS0864 | pJS0284, pJS0281, pSC31_3 | $RECEIVER_{J23115(*)}$, $P_{lux}$:*sfgfp* | S(6,9-11) |
| sJS0863 | pJS0283, pJS0281, pSC31_3 | $RECEIVER_{J23105}$, $P_{lux}$:*sfgfp* | S6 |
| sJS0862 | pJS0282, pJS0281, pSC31_3 | $RECEIVER_{J23107}$, $P_{lux}$:*sfgfp* | S6 |
| | | | |
| sJS1201 | pJS0277, pJS0275, pSC31_3 | SENSOR-MUX, $P_{tet}$:*sfgfp* | S7 |
| sJS0830 | pJS0277, pJS0260, pSC31_3 | SENSOR-MUX, $P_{tac}$:*sfgfp* | S7 |
| sJS1202 | pJS0277, pJS0304, pSC31_3 | SENSOR-MUX, $P_{PhlF}$:*sfgfp* | S7 |
| sJS1203 | pJS0277, pJS0307, pSC31_3 | SENSOR-MUX, P1:*sfgfp* | S7 |
| sJS1204 | pJS0277, pJS0309, pSC31_3 | SENSOR-MUX, P9:*sfgfp* | S7 |
| sJS1205 | pJS0277, pJS0310, pSC31_3 | SENSOR-MUX, P4:*sfgfp* | S7 |
| sJS1208 | pJS0277, pJS0311, pSC31_3 | SENSOR-MUX, P5:*sfgfp* | S7 |
| sJS1209 | pJS0277, pJS0306, pSC31_3 | SENSOR-MUX, P3:*sfgfp* | S7 |
| sJS1206 | pJS0277, pJS0312, pSC31_3 | SENSOR-MUX, P2:*sfgfp* | S7 |
| sJS1207 | pJS0277, pJS0337, pSC31_3 | SENSOR-MUX, P6:*sfgfp* | S7 |
| | | | |
| sJS1219 | pJS0350, pJS0275, pSC31_3 | aTc-NOT6*, $P_{tet}$:*sfgfp* | S8 |
| sJS1112 | pJS0350, pJS0200, pSC31_3 | aTc-NOT6*, P6*:*sfgfp* | S8 |
| sJS1011 | pJS0143, pJS0200, pSC31_3 | P6*:*sfgfp* | 4, S(8,15) |
| | | | |
| sJS1263 | pJS0333, pJS0326, pSC31_3 | AHL-DEMUX, $P_{lux*}$:*sfgfp* | 5, S(13,15) |
| sJS1262 | pJS0333, pJS0304, pSC31_3 | AHL-DEMUX, $P_{PhlF}$:*sfgfp* | 5, S13 |
| sJS1259 | pJS0333, pJS0306, pSC31_3 | AHL-DEMUX, P3:*sfgfp* | 5, S13 |
| sJS1260 | pJS0333, pJS0305, pSC31_3 | AHL-DEMUX, P8:*sfgfp* | 5, S(13,15) |
| sJS1251 | pJS0333, pJS0308, pSC31_3 | AHL-DEMUX, P7:*sfgfp* | 5-7, S(13,15) |
| sJS1261 | pJS0333, pJS0309, pSC31_3 | AHL-DEMUX, P9:*sfgfp* | 5, S13 |
| sJS1252 | pJS0333, pJS0312, pSC31_3 | AHL-DEMUX, P2:*sfgfp* | 5-7, S13 |
| | | | |
| sJS1243 | pJS0357, pJS0281, pSC31_3 | AHL-DEMUX v0.1, $P_{lux}$:*sfgfp* | S9 |
| sJS1244 | pJS0357, pJS0304, pSC31_3 | AHL-DEMUX v0.1, $P_{PhlF}$:*sfgfp* | S9 |
| sJS1246 | pJS0357, pJS0306, pSC31_3 | AHL-DEMUX v0.1, P3:*sfgfp* | S9 |

| | | | |
|---|---|---|---|
| sJS1245 | pJS0357, pJS0305, pSC31_3 | AHL-DEMUX v0.1, P8:*sfgfp* | S9 |
| sJS1248 | pJS0357, pJS0308, pSC31_3 | AHL-DEMUX v0.1, P7:*sfgfp* | S9 |
| sJS1247 | pJS0357, pJS0309, pSC31_3 | AHL-DEMUX v0.1, P9:*sfgfp* | S9 |
| sJS1249 | pJS0357, pJS0312, pSC31_3 | AHL-DEMUX v0.1, P2:*sfgfp* | S9 |
| | | | |
| sJS1073 | pJS0314, pJS0281, pSC31_3 | AHL-DEMUX v0.2, $P_{lux}$:*sfgfp* | S(10,11) |
| sJS1070 | pJS0314, pJS0304, pSC31_3 | AHL-DEMUX v0.2, $P_{PhlF}$:*sfgfp* | S(10,11) |
| sJS1071 | pJS0314, pJS0306, pSC31_3 | AHL-DEMUX v0.2, P3:*sfgfp* | S(10,11) |
| sJS1074 | pJS0314, pJS0305, pSC31_3 | AHL-DEMUX v0.2, P8:*sfgfp* | S(10,11) |
| sJS1037 | pJS0314, pJS0308, pSC31_3 | AHL-DEMUX v0.2, P7:*sfgfp* | S(10,11) |
| sJS1072 | pJS0314, pJS0309, pSC31_3 | AHL-DEMUX v0.2, P9:*sfgfp* | S(10,11) |
| sJS1038 | pJS0314, pJS0312, pSC31_3 | AHL-DEMUX v0.2, P2:*sfgfp* | S(10,11) |
| | | | |
| sJS1328 | pJS0284, pJS0326, pSC31_3 | $RECEIVER_{J23115(*)}$, $P_{lux*}$:*sfgfp* | 5, S(12-15) |

## Appendix References


Andersen JB, Sternberg C, Poulsen LK, Bjorn SP, Givskov M & Molin S (1998) New unstable variants of green fluorescent protein for studies of transient gene expression in bacteria. *Appl. Environ. Microbiol.* **64:** 2240–2246

Andrews LB, Nielsen AAK & Voigt CA (2018) Cellular checkpoint control using programmable sequential logic. *Science* **361:**

de Boer HA, Comstock LJ & Vasser M (1983) The tac promoter: a functional hybrid derived from the trp and lac promoters. *Proc. Natl. Acad. Sci. U.S.A.* **80:** 21–25

Chen Y-J, Liu P, Nielsen AAK, Brophy JAN, Clancy K, Peterson T & Voigt CA (2013) Characterization of 582 natural and synthetic terminators and quantification of their design constraints. *Nat. Methods* **10:** 659–664

Cox RS, Surette MG & Elowitz MB (2007) Programming gene expression with combinatorial promoters. *Mol. Syst. Biol.* **3:** 145

Flagan S, Ching W-K & Leadbetter JR (2003) Arthrobacter strain VAI-A utilizes acyl-homoserine lactone inactivation products and stimulates quorum signal biodegradation by Variovorax paradoxus. *Appl. Environ. Microbiol.* **69:** 909–916

Gander MW, Vrana JD, Voje WE, Carothers JM & Klavins E (2017) Digital logic circuits in yeast with CRISPR-dCas9 NOR gates. *Nat Commun* **8:** 15459

Lou C, Stanton B, Chen Y-J, Munsky B & Voigt CA (2012) Ribozyme-based insulator parts buffer synthetic circuits from genetic context. *Nat. Biotechnol.* **30:** 1137–1142

Lutz R & Bujard H (1997) Independent and tight regulation of transcriptional units in Escherichia coli via the LacR/O, the TetR/O and AraC/I1-I2 regulatory elements. *Nucleic Acids Res.* **25:** 1203–1210

Mutalik VK, Guimaraes JC, Cambray G, Lam C, Christoffersen MJ, Mai Q-A, Tran AB, Paull M, Keasling JD, Arkin AP & Endy D (2013) Precise and reliable gene expression via standard transcription and translation initiation elements. *Nat. Methods* **10:** 354–360

Newville M, Stensitzki T, Allen DB & Ingargiola A (2014) LMFIT: Non-linear least-square minimization and curve-fitting for Python Zenodo Available at: https://zenodo.org/record/11813 [Accessed March 16, 2019]

Nielsen AAK, Der BS, Shin J, Vaidyanathan P, Paralanov V, Strychalski EA, Ross D, Densmore D & Voigt CA (2016) Genetic circuit design automation. *Science* **352:** aac7341

Nielsen AAK & Voigt CA (2014) Multi-input CRISPR/Cas genetic circuits that interface host regulatory networks. *Mol. Syst. Biol.* **10:** 763

Olson EJ, Hartsough LA, Landry BP, Shroff R & Tabor JJ (2014) Characterizing bacterial gene circuit dynamics with optically programmed gene expression signals. *Nat. Methods* **11:** 449–455

Pédelacq J-D, Cabantous S, Tran T, Terwilliger TC & Waldo GS (2006) Engineering and characterization of a superfolder green fluorescent protein. *Nat. Biotechnol.* **24:** 79–88

Ptashne M (2004) A genetic switch: phage lambda revisited 3rd ed. Cold Spring Harbor, N.Y: Cold Spring Harbor Laboratory Press

Qi LS, Larson MH, Gilbert LA, Doudna JA, Weissman JS, Arkin AP & Lim WA (2013) Repurposing CRISPR as an RNA-guided platform for sequence-specific control of gene expression. *Cell* **152:** 1173–1183

Ramakrishnan P & Tabor JJ (2016) Repurposing Synechocystis PCC6803 UirS-UirR as a UV-violet/green photoreversible transcriptional regulatory tool in E. coli. *ACS Synth Biol* **5:** 733–740

Schaefer AL, Hanzelka BL, Parsek MR & Greenberg EP (2000) Detection, purification, and structural elucidation of the acylhomoserine lactone inducer of Vibrio fischeri luminescence and other related molecules. *Meth. Enzymol.* **305:** 288–301

Shin J, Zhang S, Der BS, Nielsen AA & Voigt CA (2020) Programming Escherichia coli to function as a digital display. *Mol. Syst. Biol.* **16:** e9401

Stanton BC, Nielsen AAK, Tamsir A, Clancy K, Peterson T & Voigt CA (2014) Genomic mining of prokaryotic repressors for orthogonal logic gates. *Nat. Chem. Biol.* **10:** 99–105

Tabor JJ, Salis HM, Simpson ZB, Chevalier AA, Levskaya A, Marcotte EM, Voigt CA & Ellington AD (2009) A synthetic genetic edge detection program. *Cell* **137:** 1272–1281

Zhang S & Voigt CA (2018) Engineered dCas9 with reduced toxicity in bacteria: implications for genetic circuit design. *Nucleic Acids Res.* **46:** 11115–11125
