# Supplementary figures and images for: Multiplexing cell‐cell communication
Source: Mol Syst Biol. 2020 Jul 16;16(7):e9618. doi: 10.15252/msb.20209618 (PMC7365139; doi:10.15252/msb.20209618)

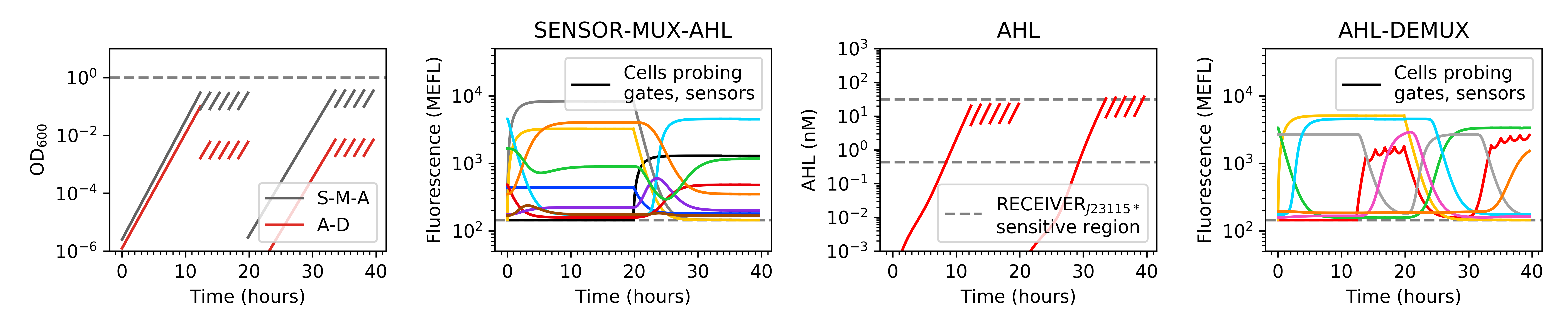

Supplement: Supplementary file 5 — Code EV1 [file MSB-16-e9618-s005.zip › fig7_multiplexing_dynamics_simulation.png]

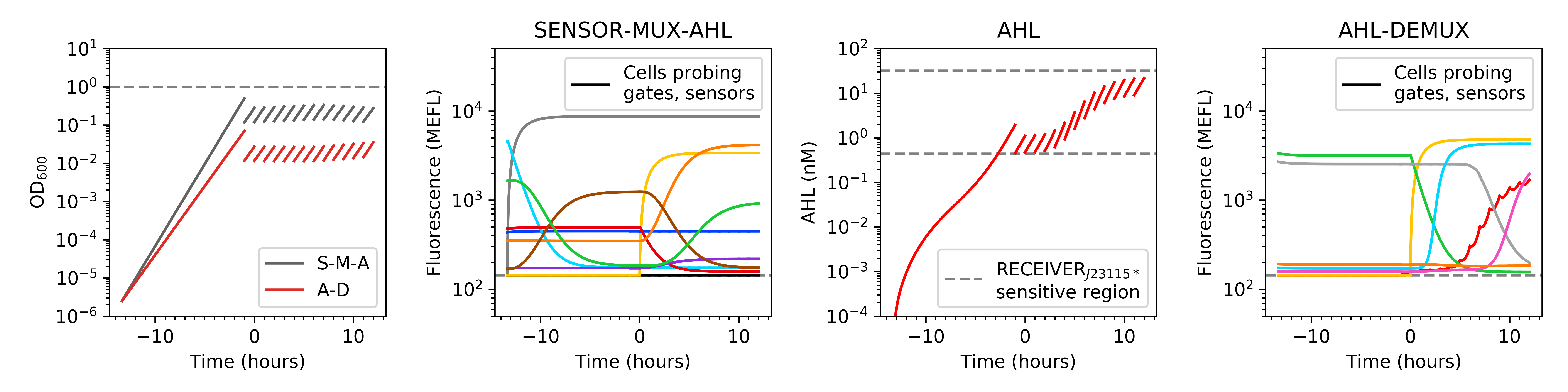

Supplement: Supplementary file 5 — Code EV1 [file MSB-16-e9618-s005.zip › figS15_dapg_response_dynamics_simulation.png]

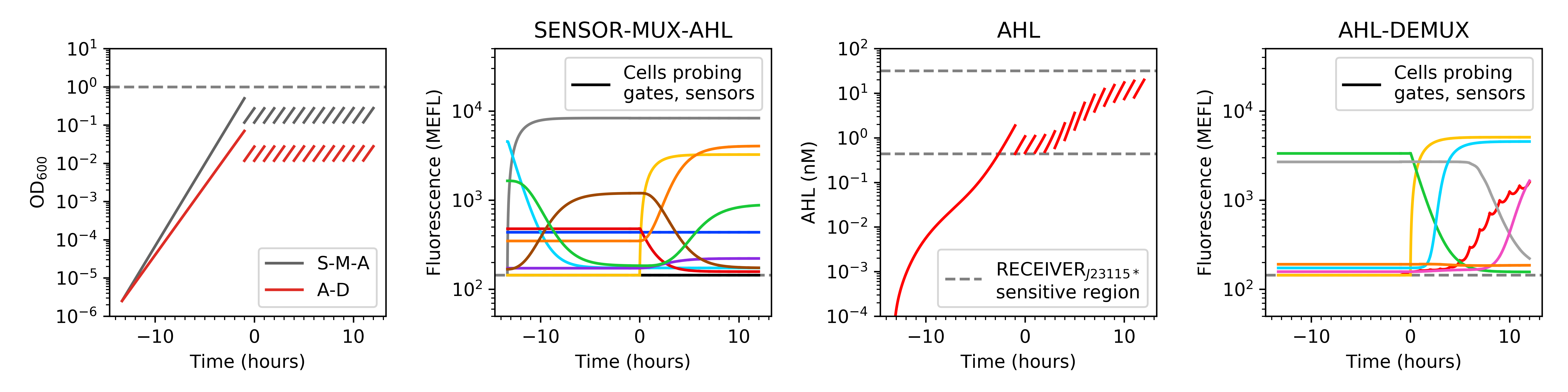

Supplement: Supplementary file 5 — Code EV1 [file MSB-16-e9618-s005.zip › figS15_dapg_response_dynamics_simulation_naive.png]
